# Implementation of a Central Sensorimotor Integration Test for Characterization of Human Balance Control During Stance

**DOI:** 10.3389/fneur.2018.01045

**Published:** 2018-12-13

**Authors:** Robert J. Peterka, Charles F. Murchison, Lucy Parrington, Peter C. Fino, Laurie A. King

**Affiliations:** ^1^Department of Neurology, Oregon Health and Science University, Portland, OR, United States; ^2^National Center for Rehabilitative Auditory Research, VA Portland Health Care System, Portland, OR, United States

**Keywords:** balance, balance control, orientation, sensory integration, sensorimotor, system identification, stance

## Abstract

Balance during stance is regulated by active control mechanisms that continuously estimate body motion, via a “sensory integration” mechanism, and generate corrective actions, via a “sensory-to-motor transformation” mechanism. The balance control system can be modeled as a closed-loop feedback control system for which appropriate system identification methods are available to separately quantify the sensory integration and sensory-to-motor components of the system. A detailed, functionally meaningful characterization of balance control mechanisms has potential to improve clinical assessment and to provide useful tools for answering clinical research questions. However, many researchers and clinicians do not have the background to develop systems and methods appropriate for performing identification of balance control mechanisms. The purpose of this report is to provide detailed information on how to perform what we refer to as “central sensorimotor integration” (CSMI) tests on a commercially available balance test device (SMART EquiTest CRS, Natus Medical Inc, Seattle WA) and then to appropriately analyze and interpret results obtained from these tests. We describe methods to (1) generate pseudorandom stimuli that apply cyclically-repeated rotations of the stance surface and/or visual surround (2) measure and calibrate center-of-mass (CoM) body sway, (3) calculate frequency response functions (FRFs) that quantify the dynamic characteristics of stimulus-evoked CoM sway, (4) estimate balance control parameters that quantify sensory integration by measuring the relative contribution of different sensory systems to balance control (i.e., sensory weights), and (5) estimate balance control parameters that quantify sensory-to-motor transformation properties (i.e., feedback time delay and neural controller stiffness and damping parameters). Additionally, we present CSMI test results from 40 subjects (age range 21–59 years) with normal sensory function, 2 subjects with results illustrating deviations from normal balance function, and we summarize results from previous studies in subjects with vestibular deficits. A bootstrap analysis was used to characterize confidence limits on parameters from CSMI tests and to determine how test duration affected the confidence with which parameters can be measured. Finally, example results are presented that illustrate how various sensory and central balance deficits are revealed by CSMI testing.

## Introduction

Human standing balance control is widely understood to be organized as a closed-loop feedback-control system. In a closed loop control system, different subsystems contribute to behavior of the entire system. The various subsystems interact with one another such that it can be problematic to attribute abnormal behavior to a particular subsystem. For balance control, these subsystems include (1) sensory systems (mainly proprioception, vision, and vestibular) that encode body orientation, (2) a sensory integration mechanism that combines sensory cues, (3) a motor activation mechanism that generates joint torques that correct for deviations from a desired orientation, and (4) body and muscle/tendon mechanics. A full appreciation of the feedback control nature of the system has motivated the application of system identification methods that are appropriate for measuring the dynamic properties of a closed-loop system and characterizing subsystems involved in balance control.

Traditional assessment of standing balance monitors spontaneous sway in different conditions that alter the available sensory cues or change the difficulty of making effective control actions [for review see (1)]. Commonly, stance is tested with eyes open, eyes closed, on firm or compliant (foam) surfaces, in different foot placement configurations (e.g., tandem, single leg), or in conditions that are specifically designed to limit the availability of proprioceptive or visual cues (e.g., sway-referencing methods used on EquiTest sensory organization tests, SOT) (2). Performance is monitored using pass/fail criteria or instrumentation is used to record signals related to body sway using, for example, force plate measures of center-of-pressure (CoP) displacements, inertial measurement sensors, or motion capture systems. For instrumented systems, the recorded signals are processed to obtained measures of variability and magnitude of the signals (3). Then the values of sway measures in specific test conditions or changes in sway measures across different test conditions give an indication of standing balance performance in relation to normative measures and provide an indirect indication about the integrity of sensory systems contributing to balance.

There are, however, limitations to assessments based on spontaneous sway measures because sensory and motor system properties cannot be separately evaluated. For example, excessive sway can be due to inadequate motor control (e.g., too little corrective torque generated per unit of body sway) or due to poor quality (low gain or high variability) of sensory systems contributing to balance (4). Another example would be a fall on an eyes closed SOT test condition with surface sway referencing. A fall could occur if (1) subjects have reduced or absent vestibular function, (2) central processing of vestibular information is inadequate (e.g., faulty central integration of semicircular canal and otolith signals), (3) the subject did not transition quickly enough to full reliance on vestibular information for balance from the sensory utilization configuration prior to the start of sway referencing (where subjects use primarily proprioceptive cues for balance control), or (4) the subject did not generate enough corrective torque due to motor control deficiency.

Application of appropriate system identification methods can overcome some of the limitations of balance assessment based on spontaneous sway measures. To disambiguate the cause/effect relationships between sensory processing, motor action, and body sway in a closed-loop control system, an external balance perturbation must be applied and then proper methods must be used to evaluate the relationship between the external perturbation and body sway or other measures (CoP, muscle activations, joint torques) (5). To date, application of these methods has remained primarily in research environments that have specialized equipment needed to apply controlled balance perturbations. Additionally, not all researchers are necessarily familiar with the mathematical methods needed for system identification. To increase access to these methods this report gives a detailed description of a methodology that is becoming more widely used (6–16). Additionally, the test equipment that we used for data collected in this study is commercially available (SMART EquiTest CRS, Natus Medical Inc., Seattle WA). This device includes research module software that allows for the delivery of custom balance perturbations that are needed for application of the methods we describe.

This report describes the methods we have employed in developing and implementing a test battery we refer to as the central sensorimotor integration (CSMI) test. These aspects include (1) modifications of an EquiTest device to obtain calibrated measures of center-of-mass (CoM) body sway, (2) description of our test protocol and the wide-bandwidth stimulus used for balance perturbations, (3) description of the frequency domain analysis methods used to obtain measures of frequency response function (FRFs) that provide a non-parametric representation of dynamic characteristics of the balance control system, and (4) description of two versions of a simple mathematical model of the balance control system. For both models, parameters were adjusted using an optimal estimation procedure to obtain a set of functionally meaningful parameters that separately identify sensory integration and motor control mechanisms.

A primary goal of this report is to encourage wider application of CSMI test methods to facilitate potential clinical applications for improved diagnosis of balance disorders. To this end we (1) describe the methodology for the CSMI test with accompanying normative data, (2) determine the reliability of parameter estimates as a function of test duration, (3) compare parameters obtained from two versions of balance control models, (4) evaluate whether parameter estimates were significantly affected using measures of CoM sway based on filtering of CoP compared to a more direct measure, and (5) provide supplementary material that includes computer programs to define stimuli and analyze CSMI test results. Finally, we present example CSMI test results from patient populations illustrating applications of these methods to populations with mild traumatic brain injury (mTBI), vestibular deficits, and other balance deficits.

## Materials and Methods

### Participants

This report utilizes a subset of data from a broader investigation into CSMI problems in patients with chronic mTBI (17). Participants included 40 healthy individuals: 13 males, 27 females, age range 21–59 years, 33.7 ± 11.5 years, height 1.69 ± 0.09 m, weight 69.8 ± 15.8 kg (mean ± sd) with no known musculoskeletal or neurological deficits. Data from one subject with mTBI and one additional control subject with unexpected balance behavior were also included to illustrate capabilities of the CSMI test. Further, data from two previous investigations were included to illustrate the effects of vestibular loss. This study was carried out in accordance with a protocol approved by the Joint Institutional Review Board Committee of Oregon Health and Science University and Veterans Administration Portland Health Care System. Additional results illustrating the effects of vestibular deficits were from studies carried out in accordance with a protocol approved by the Institutional Review Board Committee of Oregon Health and Science University. All subjects gave written informed consent prior to the start of experimental procedures in accordance with the Declaration of Helsinki.

### Equipment

Standing balance was tested on a modified SMART EquiTest CRS device (Natus Medical Inc, Seattle WA) running software version 8.6.0. This device has motorized drives that allow forward/backward translations or toe up/toe down rotations of the stance surface and sagittal plane rotations of the visual surround. Subjects stand on dual force plates that record 3D forces and moments. Maximum specified surface and visual surround rotational velocities are 50°/s and 15°/s, respectively, which are well above the largest velocites used in this study (2°/s). Maximum accelerations were not specified, but were found to be sufficient to deliver stimuli used in this study.

The EquiTest CRS device includes a Research Module that allows for creation of user-defined tests. We used the Research Module to define a custom protocol that used sampled stimulus waveforms created in Matlab (The Mathworks Inc., Natick MA, USA) to generate continuous surface and/or visual surround rotations that evoke anterior-posterior (AP) body sway in test subjects (see Supplementary Materials for Matlab programs that create our stimuli).

The EquiTest device was modified in two ways: (1), a floor and wall-mounted external frame was placed near the EquiTest that supported two “sway rod” devices (described below) that were used to directly measure AP body displacements at hip and shoulder levels, and (2), a plaid-patterned poster (112 cm high × 106 cm wide) with pseudorandomly placed vertical and horizontal black, white, and gray stripes lined the visual surround to provide a rich visual stimulus to enhance responses to visual stimuli (see Supplementary Figure 1).

### Stimulus Generation and Test Conditions

Subjects were tested in 8 test conditions that included 4 test types (surface-tilt with eyes closed, surface tilt with eyes open viewing a fixed visual surround, visual surround tilt with eyes open with stance on a level surface, and combined surface-tilt and visual-tilt with eyes open) with each test type performed at 2 amplitudes (2 and 4° peak-to-peak). Tests were presented in randomized order.

The surface and visual tilt stimuli were based on a pseudorandom maximal length ternary number sequence consisting of 80 numbers with +1, 0, or −1 values [generated using a 4-stage shift register with feedback; (18)]. The number sequence was transformed into a time series waveform by substituting each number in the sequence with a set of 25 time samples of equal value to create a waveform consisting of 2,000 samples for one stimulus cycle corresponding to a cycle period of 20 s for 100/s sampling. This time series was mathematically integrated and the integrated waveform was scaled to have peak-to-peak values of 2 and 4°. Additionally, the starting point in the number sequence was selected so that the integrated waveform had a non-zero mean such that about 80% of the integrated waveform had positive values giving stimuli that were biased to favor toe-down surface rotations and forward visual surround tilts since subjects can tolerate greater forward than backward sway without loosing balance.

A Fourier transform of the stimulus waveform demonstrates that a waveform created by a maximal length ternary sequence has the property that stimulus energy is only present at the fundamental frequency (fundamental frequency in Hz is 1/cycle duration = 0.05 Hz) and odd harmonic multiples of the fundamental frequency. Additionally, the magnitude of frequency spectral components of the waveform based directly on a maximal length ternary number sequence is approximately flat out to a frequency of about 2 Hz = 1/(2^*^25 samples per sequence number/100 samples/s) and then diminishes. Since we use the mathematically integrated waveform to control the angular tilt position of the surface or visual surround, the magnitude of spectral components of the integrated stimulus declines in proportion to inverse frequency [see Figure 3 in (19) to see power spectrum representation of a stimulus nearly identical to our stimulus].

Twelve single-cycle waveforms were concatenated to give a final stimulus waveform with a total duration of 246 s that included 2 s no-movement segments at the beginning and 4 s at the end. The stimulus waveform was low-pass filtered at 4.5 Hz to reduced higher frequency components that the EquiTest device could not faithfully deliver. The sample rate for stimulus delivery and data collection was 100/s, the maximum rate allowed by the EquiTest research module software. The stimuli were created in Matlab and were saved as ASCII text files that were imported by the EquiTest Research Module software to define experimental tests.

Subjects feet were placed on the stance surface with ankles aligned with the surface rotation axis and at height-dependent stance widths according to EquiTest instructions for performing the clinical SOT. Subjects wore ear protection to mask room and motor sounds.

Following a calibration trial (see below), a warmup test was performed to familiarize subjects with the balance perturbations. The warmup trial was a 4° surface-tilt test that was performed eyes open for the first 120 s and then eyes closed for the remainder of the trial. Then the 8 different tests were performed in randomized order with 3 min breaks given after every 3 trials.

### Sway Measurements

Body sway measurements were obtained from measures of whole body CoP displacements from the surface force plates and from measurements of AP body displacements at hip and shoulder level made using a custom “sway rod” system. Each sway rod system consisted of a potentiometer (Midori model CP-2UTN, Midori America Corporation, Irvine CA) attached to an earth-fixed frame located behind and to the subject's left. The potentiometer rotation axis was vertically aligned. The locations of the potentiometers were adjustable in height and in AP depth on the frame and were placed at hip and shoulder heights. A thin 61 cm length aluminum shaft was flexibly attached to the frame-mounted potentiometers to allow free vertical plane rotation of the sway rods without causing potentiometer rotation. The sway rod shafts extended behind the subject with the distal end of the sway rod resting in hip and shoulder hooks mounted on the subject's back at midline. The hip hook was approximately at the hip joint level and the shoulder hook was just below shoulder joint height. Sway rod height above the stance surface and length of the sway rod from the potentiometer to the hook were measured for both hip and shoulder sway rods for use in off-line calculation of body displacements. To facilitate accurate AP placement of the sway rod potentiometer on the frame, each potentiometer module included electronics that lit an LED to signal when the sway rod was parallel to the subject's frontal plane when the subject was standing upright. In this position, the potentiometer registered zero volts. As subjects swayed forward or backward, the sway rod shaft could slide freely in its hook and rotate the potentiometer producing a voltage change proportional to the potentiometer rotation angle and related, by trigonometry, to AP body displacement at hook levels. The potentiometer electronics module included a scaling amplifier with scaling set to 1 Volt/3 degrees. The potentiometer signals were recorded on auxiliary channels in the EquiTest system that could accept +/−10 V signals that were digitized by a 12-bit A/D converter. Additional description of the sway rod system is included in Supplementary Figure 1.

The EquiTest Research Module records various force plate signals, signals encoding surface rotation and translation, and visual surround rotation. Of relevance to the current study are AP CoP displacement, vertical force measures used to measure subject weight, surface rotation angle, visual surround rotation angle, and sway rod potentiometer angles. The EquiTest software encodes data in a proprietary binary format with access to the data provided by exporting test results to ASCII files (from EquiTest software version 8.6.0) or Unicode files (from software version 9.3). One file is created for each test trial.

### Estimation of CoM Sway

#### CoM From Sway Rod Measures

A calibration trial was performed to obtain data used to derive coefficients of an equation that relates hip and shoulder potentiometer signals to a subject's CoM AP displacements and CoM AP rotation angles over the time course of each experimental test. The principle that allows this derivation is that the CoP displacement is equal to the body's horizontal projection of CoM displacement in the case of a static, unmoving body. We approximate this static case by asking the subject to move very slowly in the AP direction over the 120 s duration of a calibration trial while recording the CoP and potentiometer signals. The calibration trial was performed with eyes open on a fixed surface while viewing a stationary visual surround. The subject was directed to assume a variety of upper and lower body orientations (e.g., keeping the body straight while swaying and then with hips slightly flexed or extended while swaying slowly forward and backward).

The potentiometer signals were processed using trigonometric relations to calculate AP displacements at hip and shoulder levels from the sway rod angles measured from the potentiometers:

(1) $$\begin{array}{l} x_{h}\left(t\right)=L_{h}\tan\theta_{h}\left(t\right) \end{array}$$

(2) $$\begin{array}{l} x_{s}\left(t\right)=L_{s}\tan\theta_{s}\left(t\right) \end{array}$$

where *L* is the length of the sway rod from the potentiometer to the hook when subjects were in an upright stance position, θ(*t*) is the sway rod angle over time measured by appropriately scaling the voltage recorded by the potentiometer, *x*(*t*) is the calculated AP displacement of the body, and *h* and *s* subscripts indicate hip and shoulder.

A least squared error fit was made to estimate parameters *A*_*h*_, *A*_s_, and *B* of an equation relating AP body displacement at hip and shoulder levels to the measured AP CoP displacement, *x*_*cop*_(*t*), to minimize the squared difference between *x*_*cop*_ and *x*_*cop*_*fit*_ defined as:

(3) $$\begin{array}{l} x_{cop\text{\_}fit}\left(t\right)=A_{h}\cdot x_{h}\left(t\right)+A_{s}\cdot x_{s}\left(t\right)+B \end{array}$$

On subsequent experimental tests, the *A*_*h*_, *A*_s_, and *B* parameters derived from the calibration test, was applied to hip and shoulder displacements recorded on experimental tests (*x*_*h*_exp_(*t*) and *x*_*s*_exp_(*t*)) to obtain a CoM displacement time series *x*_*com*_(*t*) = *A*_*h*_ · *x*_*h*_exp_(*t*) + *A*_*s*_ · *x*_*s*_exp_(*t*) + *B*.

The CoM displacement time series was then used to calculate the CoM tilt angle with respect to vertical using the equation:

(4) $$\begin{array}{l} \theta_{com}\left(t\right)={\text{sin}}^{-1}\left(\frac{x_{com}\left(t\right)}{h}\right) \end{array}$$

where *h* is the CoM height above the ankle joint. The CoM height estimate was obtained following (20) using measures of leg length (medial malleolus to femoral condyles), thigh length (femoral condyles to greater trochanter), and HAT length (head, arms, trunk segment measured from greater trochanter to glenohumeral joint). Additionally, these body segment length measures along with a body mass measure (obtained from vertical forces measured by the EquiTest device) provided an estimate of the body moment of inertial, *J* (units: kg m^2^), of the legs, thighs, and HAT segments about the ankle joint axis. Along with *J*, subject mass *m*, (in kg) and *h* (in m) were parameters needed in the balance control model that was used to calculate sensory integration and neural control parameters representing each subject's balance performance characteristics.

#### CoM From Filtered Cop

While the direct measurements of hip and shoulder displacements provide a relatively simple method for measuring CoM displacement using the methods described above, an even simpler method, based on lowpass filtering of CoP, may provide sufficiently accurate CoM displacement measures. An approximate relationship between CoP and CoM displacement is given by Winter et al. (21):

(5) $$\begin{array}{l} x_{cop}\left(t\right)=x_{com}\left(t\right)-\frac{J}{W\cdot h}\cdot {\ddot{x}}_{com}\left(t\right) \end{array}$$

Where ẍ_*com*_ is CoM acceleration, *J* is body moment of inertia about the ankle joint, and *W* is body weight excluding the feet. At any given frequency of body motion, *x*_*com*_ and −ẍ_*com*_ are in phase with one another so *x*_*cop*_ will also be in phase with *x*_*com*_. Furthermore, the amplitude of ẍ_*com*_ increases as the square of the frequency of *x*_*com*_ and thus makes an increasing contribution to *x*_*cop*_ as frequency increases. Because the CSMI methods for quantifying balance control are focused on frequencies below about 1.5 Hz, it may be possible to apply a lowpass filter to the recorded CoP to greatly diminish the CoM acceleration contribution to CoP and obtain a CoM displacement measure (22).

We explored the use of a lowpass filtered CoP to estimate CoM displacement and characterized the extent to which use of this simpler CoM measure affected results in comparison to use of CoM from our sway rod measurement method. We defined filter coefficients of a 1st order Butterworth filter and applied it using the Matlab “filtfilt” function to provide phaseless 2nd order filtering of the CoP data for each trial in each test condition for each subject. The cutoff frequency was varied in 0.005 Hz increments from 0.25 to 0.65 Hz, the mean squared error (MSE) between the filtered CoP and the CoM from sway rod measures, and the cutoff frequency with the lowest MSE was identified. These best MSE cutoff frequencies were compared across subjects and test conditions. Then a single average best frequency across all subjects and test conditions was calculated and used to process CoP data to obtained CoM displacement, and then CoM sway angles. The CoM data from sway rod and filtered CoP were analyzed as described in the following sections with results compared to determine the extent to which a simpler CoM sway measure could provide comparable results.

### Stimulus/Response Analysis

A frequency domain analysis, following the methods of Pintelon and Schoukens (23), was applied to the recorded stimulus tilt angle and the estimated CoM body sway angle of each experimental test to calculate a frequency response function (FRF). An FRF provides a non-parametric description of the dynamic characteristics of the balance control system. The first cycle is ignored to avoid transient responses. Then an FRF is calculated by taking the discrete Fourier transform (using Matlab fft function) of each of the last 11 cycles of the stimulus and CoM sway response time series when the response is assumed to have attained steady state behavior. The assumption of steady state behavior is supported by previous results using similar stimuli that did not reveal evidence for adaptation or habituation over successive cycles on a given trial (6). The experimental FRF, *H*_*e*_, calculation is:

(6) $$\begin{array}{l} H_{e}\left(j\omega_{k}\;\right)=\frac{\sum_{i=1}^{M}X_{com}^{i}\left(j\omega_{k}\right)}{\sum_{i=1}^{M}X_{stim}^{i}\left(j\omega_{k}\right)} \end{array}$$

where $X_{com}^{i}\left(j\omega_{k}\right)$ and $X_{stim}^{i}\left(j\omega_{k}\right)$ are the Fourier transforms of CoM sway response and the stimulus of the *ith* cycle of a total of *M* = 11 cycles, *j* is the imaginary number $\sqrt{-1}$, and ω_*k*_ is radian frequency at the *kth* frequency. Note that a stimulus created by a maximal length ternary sequence has the unusual property that stimulus energy is only present at the fundamental frequency (fundamental frequency in Hz is 1/cycle duration—in this case 0.05 Hz) and odd harmonic multiples of the fundamental frequency. Therefore, all even harmonics of $X_{com}^{i}\left(j\omega_{k}\right)$ and $X_{stim}^{i}\left(j\omega_{k}\right)$ were removed prior to the above FRF calculation.

The variability of frequency components of FRFs generally increases with increasing frequency because both the relative responsiveness to the stimulus and the energy of our stimulus declines with increasing frequency. Averaging across stimulus cycles was used to reduce the variability of FRFs, and to further reduce variability, an increasing number of adjacent spectral components were averaged across frequency giving a final distribution of 12 FRF values at frequencies ranging from 0.05 to 1.75 Hz that were approximately equally spaced on a logarithmic frequency scale. The final set of 12 frequencies were at 0.05, 0.1, 0.15, 0.2, 0.3, 0.4, 0.55, 0.7, 0.9, 1.1, 1.35, 1.75 Hz. Higher frequencies were not included since the stimulus energy diminishes rapidly at higher frequencies and body sway behavior becomes increasingly influenced by multi-segment body motions (24) that are not represented by the balance control model used to parameterize the FRFs (see below),

An FRF is a set of complex numbers that vary with frequency but is commonly represented as a gain function, |*H*_*e*_(*jω*_*k*_)|, and phase function, ∠*H*_*e*_(*jω*_*k*_), given by:

(7) $$\begin{array}{l} |H_{e}\left(j\omega_{k}\right)|=\sqrt{H\left(j\omega_{k}\right)\cdot conj\left(H\left(j\omega_{k}\right)\right)} \end{array}$$

(8) $$\begin{array}{l} ∠H_{e}\left(j\omega_{k}\right)=\text{ta}{\text{n}}^{-1}\frac{Im\left(H_{e}\left(j\omega_{k}\right)\right)}{Re\left(H_{e}\left(j\omega_{k}\right)\right)} \end{array}$$

where *conj* is the complex conjugate operator, *Im* and *Re* are imaginary and real parts of the complex values of *H*_*e*_(*jω*_*k*_), and the subscript *e* refers to the experimental FRF.

Our frequency domain analysis also calculated a coherence function:

(9) $$\begin{array}{l} \gamma^{2}\left(\omega_{k}\;\right)=\frac{|\sum_{i=1}^{M}X_{com}^{i}\left(j\omega_{k}\right)\cdot conj\left(X_{stim}^{i}\left(j\omega_{k}\right)\right){|}^{2}}{\left(\sum_{i=1}^{M}|X_{stim}^{i}\left(j\omega_{k}\right){|}^{2}\right)\cdot \left(\sum_{i=1}^{M}|X_{com}^{i}\left(j\omega_{k}\right){|}^{2}\right)} \end{array}$$

where |^*^| indicates calculation of the magnitude of the complex numbers representing the Fourier components of the stimulus and response spectra, and *conj* is the complex conjugate operation. Coherence function values range from 0 to 1 with higher values indicating larger signal-to-noise conditions in the analysis relating the response to the stimulus. Note that when periodic stimuli are used for system identification, coherence function values only provide an indication of signal-to-noise conditions and do not indicate the presence of non-linearities in the system (23).

### Balance Control Model

**Figure 1:**
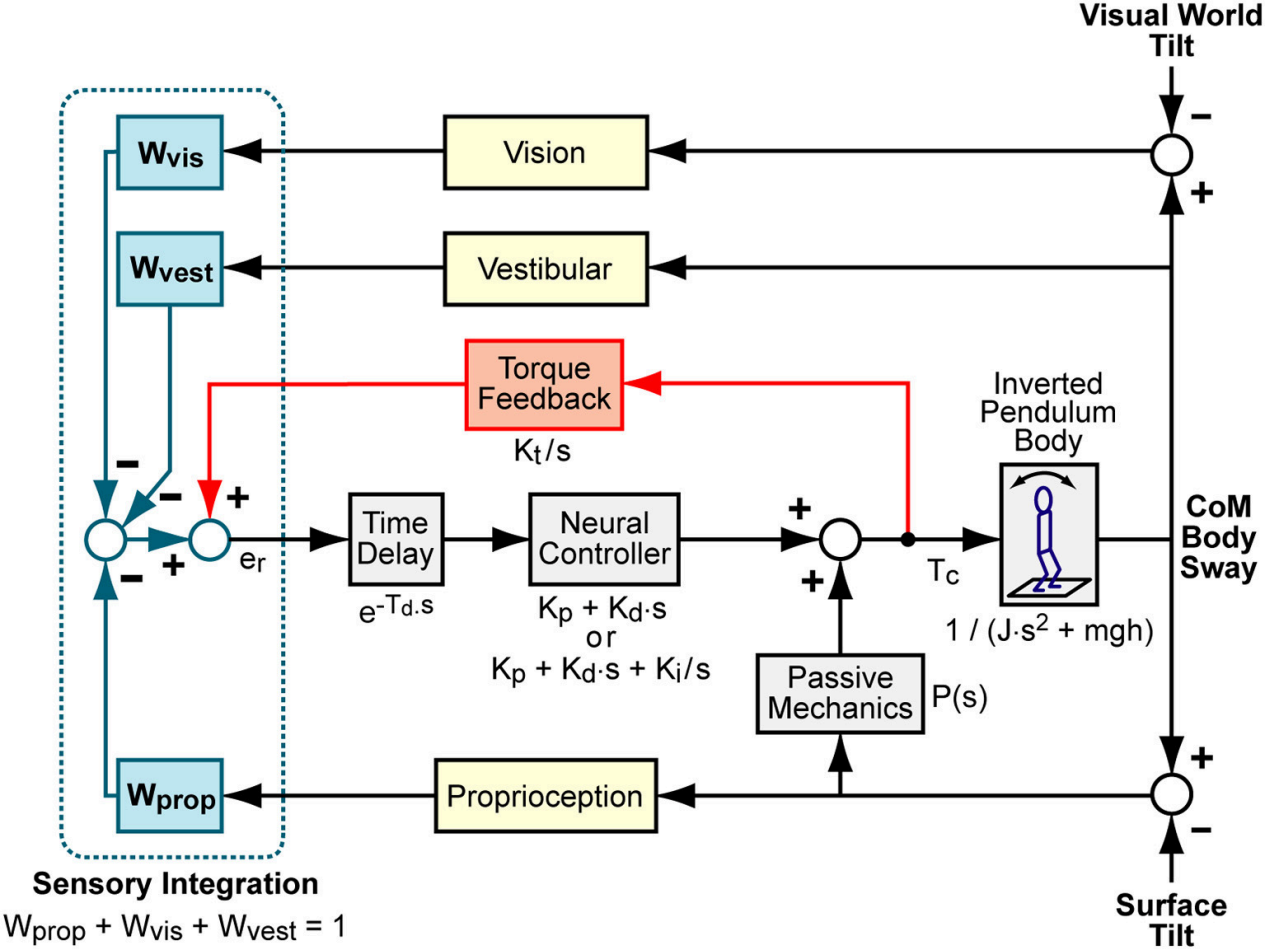
Balance control model block diagram. Visual, vestibular, and proprioception systems provide accurate measures of body orientation relative to the visual scene, earth vertical, and the stance surface, respectively. A weighted combination of these sensory sources provides an internal orientation estimate. This orientation estimate is supplemented with information regarding the mathematical integral of overall corrective ankle torque, *T*_*c*_, via a positive “Torque Feedback” loop. The signs on the summations within the “Sensory Integration” subsystem indicate whether the sensory information provides negative feedback control (from visual, vestibular, and proprioceptive systems) or positive feedback control (from torque sensors). The sensory information is used to generate time-delayed corrective torque via a “Neural Controller” and corrective torque from the neural controller is supplemented by torque due to passive muscle/tendon mechanics. The overall corrective ankle torque causes a single-segment inverted pendulum body to change orientation. Laplace transform representations of the dynamic properties of various components are shown.

We represented the balance control as a feedback control system as shown in block diagram form in Figure 1. The model represents a system regulated by a continuous, linear, time-invariant control mechanism. Previous work found no evidence for non-linear control mechanisms regulating balance in response to sustained, steady-state stimuli (25). The Figure 1 model has five major components that include (1) body mechanics of an inverted pendulum, (2) sensory integration provided by a weighted summation of orientation information from proprioceptive, visual, and vestibular systems, (3) time delayed neural controller that transforms the weighted sensory information into corrective ankle torque, (4) a torque positive feedback component that contributes to control by feeding back information related to the time integral of the corrective torque applied at the ankles, and (5) a passive component that generates ankle torque as a function ankle angle and/or angular velocity with no time delay (26). As others have demonstrated (27, 28), it is problematic to obtain reliable estimates of parameters associated with the passive component since other neural controller parameters have a very similar influence over the shape of FRFs predicted by this model. We also found it difficult to obtain reliable estimates of passive parameters. Therefore, we do not present results that include estimates of the passive component contribution, but the passive component is given in the model equations given below to illustrate its potential influence on FRFs.

The model, *H*_*m*_, can be expressed as a differential equation in the Laplace domain that predicts the CoM response, *X*_*com*_(*s*), for a given surface stimulus, *X*_*surf*_(*s*), visual stimulus, *X*_*vis*_(*s*), or combined surface plus visual stimuli. The Laplace equations for surface, visual, or combined stimuli are:

(10) $$\begin{array}{l} H_{surf}\left(s\right)=\frac{X_{com}\left(s\right)}{X_{surf}\left(s\right)}\\ \;=\frac{W_{prop}\cdot NC\cdot TD\cdot B+P\cdot B}{1-TF\cdot NC\cdot TD+P\cdot B+NC\cdot TD\cdot B} \end{array}$$

(11) $$\begin{array}{l} H_{vis}\left(s\right)=\frac{X_{com}\left(s\right)}{X_{vis}\left(s\right)}\\ \;=\frac{W_{vis}\cdot NC\cdot TD\cdot B}{1-TF\cdot NC\cdot TD+P\cdot B+NC\cdot TD\cdot B} \end{array}$$

(12) $$\begin{array}{l} H_{surf+vis}\left(s\right)=\frac{X_{com}\left(s\right)}{X_{surf+vis}\left(s\right)}\\ \;=\frac{\left(W_{prop}+W_{vis}\right)\cdot NC\cdot TD\cdot B+P\cdot B}{1-TF\cdot NC\cdot TD+P\cdot B+NC\cdot TD\cdot B} \end{array}$$

where *s* is the Laplace variable, *NC* = *K*_*p*_ + *K*_*d*_ · *s* is the neural controller (a proportional-derivative, PD, neural controller)$,\;TD=e^{-T_{d}\cdot s}$ is the time delay component, *B* is a linearized equation representing inverted pendulum body mechanics given by $\frac{1}{\left(J\cdot s^{2}-mgh\right)}$ with *m* equal to body mass minus mass of the feet and *g* the gravity constant, *TF* is the torque feedback component given by $\frac{K_{t}}{s}$, and *P*(*s*) is the passive component. Simple forms of *P* can include only a simple stiffness factor, *P* = *K*_*pas*_ or a combination of stiffness and damping *P* = *K*_*pas*_ + *B*_*pas*_ · *s*.

The torque feedback mechanism assumes that the balance control system has available to it a measure of corrective ankle torque derived from sensory sources. This torque signal is processed to eliminate higher frequency components and is added (positive feedback) to the sensory error signals derived from the other sensory systems. The combined sensory error signal is, in turn, processed to generate additional ankle torque (29). Functionally, torque feedback influences low frequency sway behavior such that the body moves toward an orientation where corrective torque is minimal (typically the upright orientation, but also can be toward orientation aligned with the gravito-inertial vector in an accelerating environment such as an accelerating train).

An alternative neural control structure used a neural controller with an integration factor *NC* = *K*_*p*_ + *K*_*d*_ · *s* + *K*_*i*_/*s* (a proportional-integral-derivative, PID, neural controller) rather than PD control with torque feedback (6, 13, 15). A model with PD control plus torque feedback has a similar, but not identical, ability to account for features of experimental FRFs as a model with PID control and no torque feedback. Because both neural control structures have been used to describe experimental results, it is of interest to understand whether the estimate of parameters shared between the two models depends on which of these two neural control structures are used in the model.

By substituting *s* = *j*ω into the above equations, model predicted FRFs can be calculated for a given set of parameter values at the same set of *k* frequencies as the experimental FRFs. Model parameters can be adjusted to optimally match the experimentally determined FRFs, thereby providing a parametric representation of the non-parametric experimental FRFs.

### Model Parameter Estimation

For each subject's FRF for each of the 8 test conditions, model parameters were estimated by adjusting the parameters to minimize an error function. This minimization was performed using the Matlab “fmincon” function from the Optimization toolbox. This function requires definition of an error function that calculates a value with each call to the error function. Our error function was:

(13) $$\begin{array}{l} E=\sum_{k=1}^{N}\frac{\left|H_{m}\left(j\omega_{k}\;\right)-H_{e}\left(j\omega_{k}\;\right)\right|}{\left|H_{m}\left(j\omega_{k}\;\right)\right|} \end{array}$$

where *N* = 12 was the number of frequency components in the FRFs and the subscripts *e* and *m* indicate the experimental and model-predicted FRF, respectively. The “fmincon” function adjusts parameters beginning with initial values to minimize the error using search criteria constraints that limit the parameters to specified ranges. The optimization procedure is not guaranteed to find a parameter set associated with a global minimum error. To overcome this, the optimization can be repeated multiple times beginning with different initial values. This is necessary when fitting more complex models (28). For the simple model applied in this study, we have found reliable convergence to the same parameter values independent of initial values. In practice and for results presented in this report, five repeated optimizations were performed and parameters associated with the lowest error were selected to represent the best fit.

On tests with very low signal-to-noise as indicated by low coherence values, the identified parameter set can be invalid in that the parameters define a system that is unstable. For example, the neural control stiffness parameter *K*_*p*_ must be greater than *mgh* (with *g* the gravity constant) for the system to be stable. Therefore, the *K*_*p*_ lower constraint is set to *mgh*. If the optimization finds a *K*_*p*_ value equal to *mgh*, the identified parameter set is obviously invalid.

For the 2 test conditions that simultaneously presented surface-tilt and visual-tilt stimuli, experimental FRFs were calculated separately relating the individual stimuli to the recorded CoM sway, model parameter estimates were obtained for each experimental FRF, and the model parameters were averaged to give a final set of parameters characterizing system properties.

### Model Quality

The ability of the model and identified parameters to account for the stimulus-evoked body sway was assessed by a variance accounted for (VAF) measure:

(14) $$\begin{array}{l} VAF=100\cdot \left(1-\frac{\sum_{i=1}^{N}{\left(\bar{\theta_{com}}\left(t_{i}\right)-\bar{\theta_{sim}}\left(t_{i}\right)\right)}^{2}}{\sum_{i=1}^{N}{\bar{\theta_{sim}}\left(t_{i}\right)}^{2}}\right) \end{array}$$

where $\bar{\theta_{com}}$ is the experimental CoM body sway averaged across the last 11 stimulus cycles, $\bar{\theta_{sim}}$ is the corresponding mean CoM body sway obtained from simulations of the Figure 1 model using Matlab Simulink (version 8.6). VAF values were calculated for each subject and on each of the 8 test conditions using the identified parameters. VAF values for both the PID and PD plus torque feedback models were calculated.

### Bootstrap Analysis

A bootstrap analysis was used (1) to characterize the distribution and range of parameter values associated with normal sensorimotor control and (2) to investigate the extent to which the accuracy and reliability of parameter estimates are influenced by reducing the number of stimulus/response cycles included in the analysis.

For each subject and each test condition the Fourier transformed stimulus/response data of M cycles were randomly selected (with replacement) from the 11 available cycles of experimental data. An FRF was calculated from these M cycles of data and model parameters were obtained. This random selection and fitting process was repeated 10,000 times and parameter sets from each of these analyses were saved for subsequent analysis. Five different bootstrap selections were made with different numbers of sampled cycles with M = 3, 6, 9, 11, 15, and 20. Thus, for each subject and each test condition we obtained 10,000 parameter sets at each of the 5 different cycle counts.

Then a second bootstrap was performed by randomly sampling (with replacement) parameters sets from the 10,000 parameters sets of the 40 subjects from the previously saved bootstrap samples for each of the 5 different cycle counts. This yielded for each model parameter a set of 10,000 samples for the 5 different cycle counts that were then statistically summarized by calculating mean and median values, and 90 and 95 percentile confidence ranges.

## Results

### Calibration Procedure

**Figure 2:**
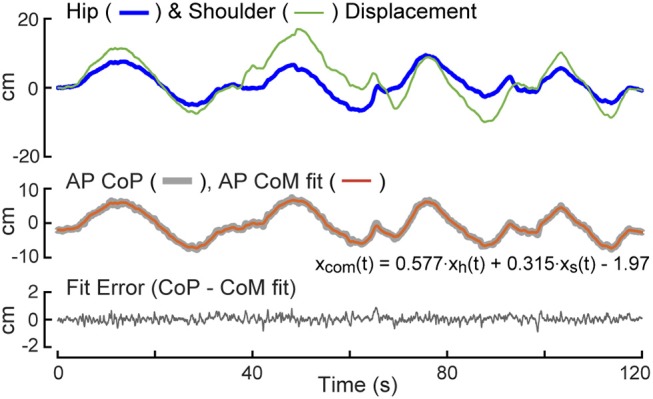
Calibration example. The test subject is instructed to sway slowly forward and backward while recording anterior-posterior (AP) center-of-pressure (CoP) displacement, and body displacements at hip, *x*_*h*_(*t*), and shoulder, *x*_*s*_(*t*), levels. A least squared error fit of a linear combination of hip and shoulder displacements to CoP provides coefficients for use in measuring center-of-mass (CoM) displacement on subsequent tests.

Data from a 2 min calibration test were used to estimate linear regression coefficients needed to transform measures of AP body displacements at hip and shoulder levels to measures of CoM displacement (Equation 3). An example of data from a calibration trial showing hip and shoulder level displacements (Equations 1, 2), the regression fit of these displacements to the AP CoP, and the fit error are provided in Figure 2. Because the subject begins the trial in an upright position, the value of the first data point in the potentiometer signal is subtracted from remainder of the points so that the calculated AP displacement represent deviations from the upright position. The regression fit accounts well for slowly varying CoP changes that are indicative of the displacement of the CoM as a function of sway-rod measured hip and shoulder displacements but not the rapid oscillations of CoP that reflect the torque corrections used by the subject to maintain the displaced CoM position. The fit error shows no obvious bias and only small rapid oscillations about a constant offset value accounted for by the *B* term in Equation 3. These small oscillations are indicative of the transient corrective torques generated to maintain the slowly moving CoM displacements. The particular values of the *A*_*h*_ and *A*_*s*_ regression coefficients depend on subject body mass distributions and the specific heights of the sway rods and *B* depends on foot placement on the surface. Across all 40 subjects, the values of the Equation 3 coefficients were *A*_*h*_ = 0.581 ± 0.056 (Mean ± SD) and *A*_*s*_ = 0.345 ± 0.035. The value of *B* is not relevant to FRF analysis since *B* only affects the mean value CoM displacement which is not used in the FRF analysis.

### Example Stimulus-Evoked Sway Analysis

**Figure 3:**
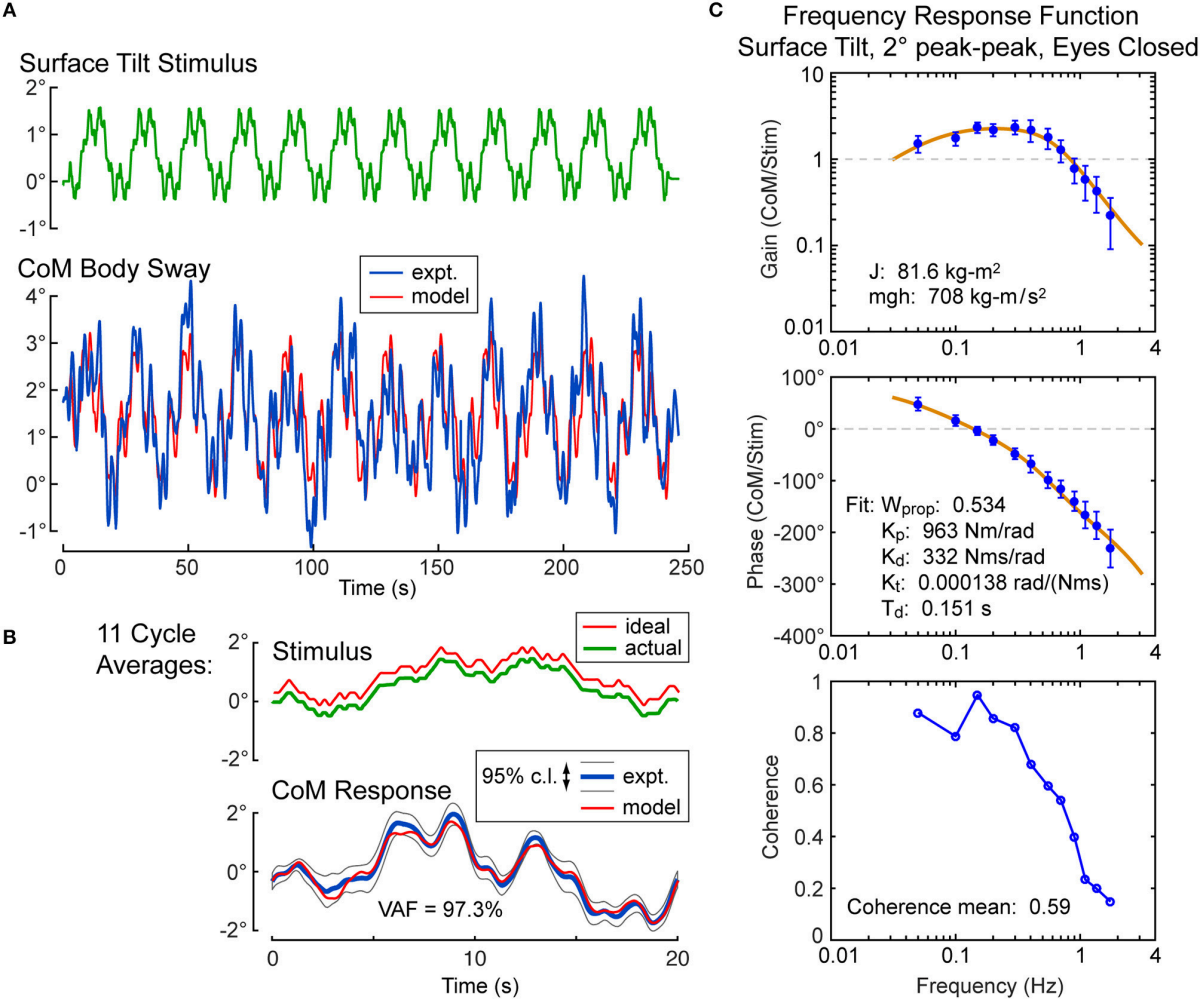
Example single-subject data and data analysis of center-of-mass (CoM) sway evoked by a surface-tilt stimulus with 2° peak-to-peak amplitude during eyes closed stance. Twelve cycles of the surface-tilt stimulus and the corresponding evoked CoM sway are shown **(A)**. Averaging across the last 11 cycles of the stimulus and sway response reveal the close correspondence between stimulus and response **(B)**. A frequency response function represented by gain and phase functions [mean values ±95% confidence limits; see (30)] characterize the balance control dynamics of this individual on this particular test with a coherence function providing information on signal-to-noise quality **(C)**. The solid lines through the frequency response data are based on model parameter estimates. Model-predicted CoM sway based on identified parameters is shown in **(A,B)** with a variance accounted for (VAF) measure showing that the model accounts for most of the experimental stimulus evoked sway. Comparison of the actual and ideal stimulus (ideal is offset from the actual for comparison) in **(B)** demonstrates the accurate delivery of the desired stimulus.

For each subject, the calibration coefficients for that subject are applied to the hip and shoulder displacement data on each stimulus trial to calculated AP CoM displacement, and then, using the estimate of the subject's CoM height above the ankle joint axis, Equation 4 was applied to calculate the AP CoM sway angle. An example of CoM sway data from a single subject and the corresponding 12-cycle surface-tilt stimulus that evoked this sway is shown in Figure 3A. There is cycle-to-cycle variability that partially obscures the relationship between the stimulus and CoM response. Averaging of CoM across the last 11 cycles clarifies the stimulus-response relationship and shows that the subject's CoM sway angle tends to track the surface tilt angle (Figure 3B) and often the sway is greater than the stimulus. For reference, if a subject was able to fully compensate for the balance perturbation caused by the rotating surface, CoM sway would not deviate from upright and the sway trace in Figures 3A,B would be flat. The focus of this paper is on the frequency domain analysis of sway responses, but time domain analyses are also performed in the analysis programs included in the Supplemental Material.

An FRF derived by application of Equation 6 to the CoM sway data is shown in Figure 3C along with the associated coherence function derived using Equation 9. The FRF is represented by gain and phase functions (Equations 7, 8) with each gain value indicating the ratio of CoM sway amplitude to the stimulus amplitude at individual frequencies and the phase indicating the relative timing between the stimulus and response. If the test subject had been a rigid mannequin whose feet were glued to the tilting surface, the mannequin's body would remain perpendicular to the surface throughout testing. The FRF analysis of the mannequin's CoM response would show gains of unity and phases of zero across all frequency components of the surface-tilt stimulus and the coherence function values would be unity (assuming no measurement noise in recording of body sway and surface tilt) indicating perfect correlation between stimulus and response. Actual human FRFs differ from the ideal mannequin behavior in several ways. CoM response gains vary with frequency. Typically gains are highest at mid-frequencies (~0.1–0.8 Hz) with values often greater than one, indicating subject sway amplitude is greater than the stimulus amplitude, and decrease for both lower and higher frequencies. Phase values cross zero at ~0.1 Hz and typically show phase leads at low frequencies and increasing phase lags at higher frequencies. Coherence values are less than one and tend to decrease with increasing frequency consistent with overall reduced signal-to-noise as sway response magnitude relative to stimulus declines with increasing frequency.

Also shown in Figure 3C is the optimization fit to the experimental FRF obtained by adjusting model parameters to minimize the Equation 13 error criterion. The model accounts well for the experimental FRF and provides a set of parameters that characterize the dynamic properties of the balance control system for each individual subject in each test condition.

### Effect of Stimulus Duration on Parameter Estimates

**Figure 4:**
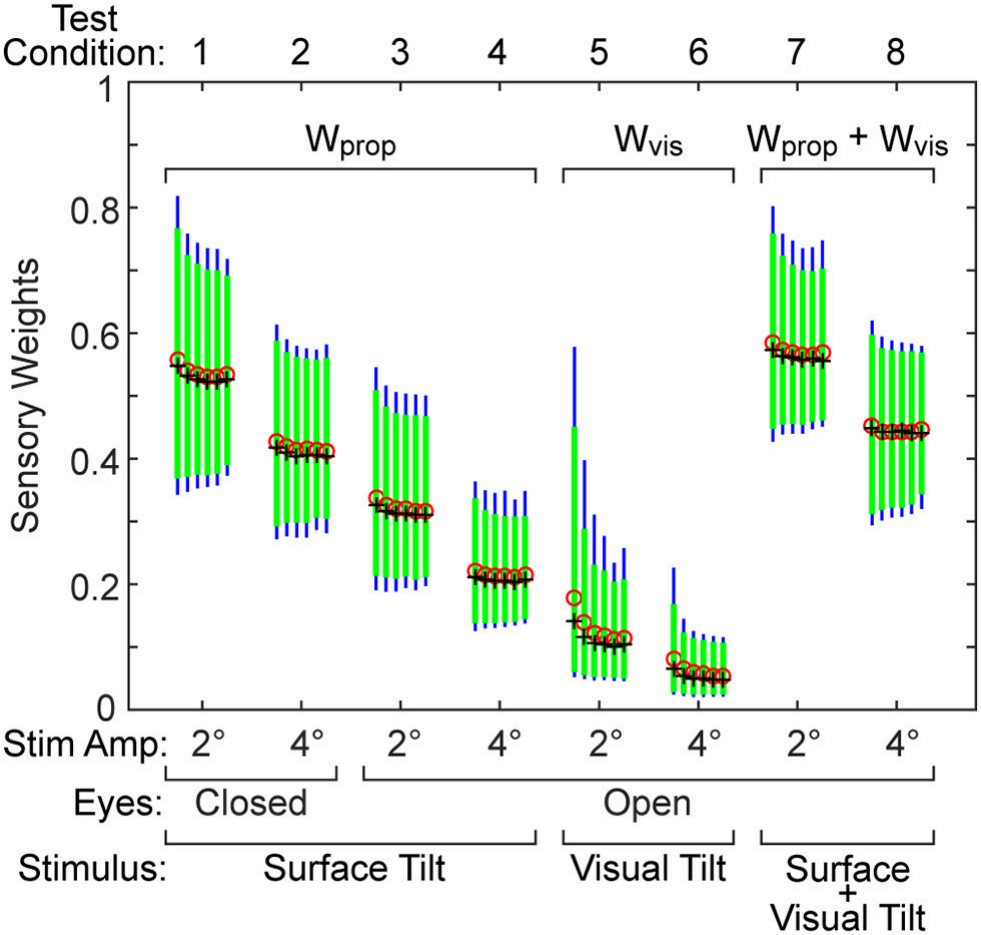
Results from a bootstrap analysis used to investigate how test duration affects the distribution of the sensory weight parameter in the eight test conditions. The five vertical bars for each test condition represent the 90th percentile (thick green bar) and 95th percentile (thin blue bar) range of the parameter when results were derived from tests that included either 3, 6, 9, 11, 15, and 20 stimulus-response cycles (arranged left to right for each condition). Mean (red o) and median (black +) values of each distribution are shown.

Shorter test durations are desirable in clinical applications, but too short a test duration likely will compromise the accuracy of parameter estimates and increase their variance. A bootstrap analysis was used to investigate the tradeoff between test duration and accuracy of parameter estimation. The results of the analysis for the sensory weight parameter on the 8 test conditions are provided in Figure 4. For each test condition there are five vertical bars showing 95th (thin bar) and 90th (thick bar) percentile confidence limits, and mean and median values corresponding to the five bootstrap analyses that included 3, 6, 9, 11, 15, and 20 cycles of data (arranged in left to right order). The percentile bars show that the distributions generally become narrower with increasing cycle counts but the narrowing diminishes with increasing cycle counts. The mean and median parameter values were greatest at the lowest cycle count suggesting bias in the parameter estimate at the lowest cycle count and indicating that 3 cycles are not sufficient to accurately estimate parameter values. The bias was largest for the visual stimulus conditions (5 and 6), which are also the conditions where the sensory weights were lowest and coherence values were lowest (see below) indicating low signal-to-noise in response to visual stimuli. Trends in narrowing distributions and reduced bias with increasing cycles counts were similar for the other model parameters.

Our conclusion is that for most test conditions, 6 stimulus cycles are adequate for the purpose of obtaining accurate parameter estimates. This judgment is based on the observation that, for most test conditions, 6 cycles were sufficient to achieve a stable variance of the parameter distribution and mean and median values showed minimal changes when cycle counts were further increased (Figure 4). However, an exception applied to the visual test conditions where the responsiveness to the stimulus and signal-to-noise were low. For these low response conditions, 11 cycles should be considered as a lower limit of test cycles. Additionally, our conclusions specifically apply to the 2 and 4° stimulus amplitudes we used. It is evident in Figure 2 that the parameter bias was greater and parameter distributions became wider for all 2° compared to 4° stimulus amplitudes suggesting that studies that use even lower stimulus amplitudes will likely need to use a greater number of stimulus cycles to avoid bias and reduce variability of parameter measures.

### Identified Balance Model Parameters

Parameters of the balance control model that included torque feedback were obtained for each of the 40 subjects on each of the 8 test conditions (Table 1). Only one subject's parameters on 1 test condition were incompatible with stability and were not include in summary statistics. Specifically, on the 2° visual stimulus condition the neural controller stiffness parameter *K*_*p*_ for this subject converged to the lower bound of *mgh* set for this parameter. The *K*_*p*_ value must be greater than *mgh*, the gravity stiffness constant, for the system to be stable. The FRF data on this individual test showed very low and variable gains and phases consistent with the low measured coherence values (mean = 0.087).

**Table 1:** Parameters derived using balance control model with PD neural control plus torque feedback.

**Condition**	**Parameter**	**Mean (SD)**	**5%tile**	**25%tile**	**50%tile**	**75%tile**	**95%tile**
1	*W_*prop*_*	0.509 (0.079)	0.367	0.461	0.512	0.557	0.676
	*K_*p*_/mgh*	1.51 (0.133)	1.31	1.42	1.50	1.56	1.79
	*K_*d*_/mgh*	0.517 (0.067)	0.421	0.465	0.521	0.565	0.627
	*K_*f*_* × 10,000	1.27 (0.46)	0.64	0.92	1.21	1.51	2.11
	*T_*d*_*	0.144 (0.015)	0.119	0.133	0.143	0.157	0.168
2	*W_*prop*_*	0.396 (0.070)	0.298	0.351	0.390	0.442	0.534
	*K_*p*_/mgh*	1.61 (0.144)	1.37	1.52	1.59	1.70	1.90
	*K_*d*_/mgh*	0.542 (0.085)	0.406	0.473	0.554	0.598	0.670
	*K_*f*_* × 10,000	1.17 (0.50)	0.48	0.77	1.13	1.52	2.21
	*T_*d*_*	0.120 (0.013)	0.098	0.111	0.117	0.128	0.145
3	*W_*prop*_*	0.299 (0.057)	0.209	0.265	0.294	0.327	0.426
	*K_*p*_/mgh*	1.61 (0.272)	1.34	1.41	1.56	1.71	2.51
	*K_*d*_/mgh*	0.523 (0.072)	0.397	0.476	0.521	0.570	0.640
	*K_*f*_* × 10,000	1.23 (0.56)	0.52	0.76	1.11	1.56	2.45
	*T_*d*_*	0.126 (0.023)	0.091	0.110	0.126	0.139	0.164
4	*W_*prop*_*	0.207 (0.046)	0.137	0.168	0.204	0.238	0.297
	*K_*p*_/mgh*	1.74 (0.341)	1.41	1.53	1.64	1.84	2.53
	*K_*d*_/mgh*	0.543 (0.095)	0.334	0.475	0.548	0.612	0.705
	*K_*f*_* × 10,000	1.35 (0.79)	0.32	0.68	1.29	1.83	3.06
	*T_*d*_*	0.092 (0.021)	0.056	0.076	0.094	0.104	0.129
5	*W_*vis*_*	0.117 (0.050)	0.051	0.086	0.107	0.135	0.216
	*K_*p*_/mgh*	1.24 (0.103)	1.12	1.19	1.22	1.28	1.53
	*K_*d*_/mgh*	0.496 (0.045)	0.412	0.467	0.501	0.524	0.596
	*K_*f*_* × 10,000	0.802 (0.894)	0.000	0.010	0.062	0.116	0.167
	*T_*d*_*	0.210 (0.025)	0.173	0.187	0.212	0.229	0.256
6	*W_*vis*_*	0.055 (0.026)	0.025	0.038	0.048	0.069	0.121
	*K_*p*_/mgh*	1,24 (0.113)	1.11	1.17	1.23	1.30	1.51
	*K_*d*_/mgh*	0.494 (0.066)	0.397	0.444	0.496	0.526	0.606
	*K_*f*_* × 10,000	0.88 (1.32)	0.00	0.11	0.47	1.06	5.11
	*T_*d*_*	0.202 (0.029)	0.146	0.178	0.211	0.226	0.240
7	*W_*prop*_ + W_*vis*_*	0.556 (0.071)	0.435	0.511	0.553	0.590	0.689
	*K_*p*_/mgh*	1.50 (0.137)	1.30	1.40	1.49	1.56	1.81
	*K_*d*_/mgh*	0.482 (0.077)	0.354	0.423	0.468	0.546	0.610
	*K_*f*_* × 10,000	1.19 (0.43)	0.46	0.92	1.17	1.38	1.98
	*T_*d*_*	0.132 (0.021)	0.090	0.122	0.133	0.148	0.160
8	*W_*prop*_ + W_*vis*_*	0.431 (0.064)	0.345	0.377	0.424	0.497	0.531
	*K_*p*_/mgh*	1.55 (0.120)	1.39	1.45	1.52	1.62	1.82
	*K_*d*_/mgh*	0.492 (0.085)	0.342	0.435	0.486	0.563	0.623
	*K_*f*_* × 10,000	1.05 (0.47)	0.34	0.73	1.00	1.47	1.92
	*T_*d*_*	0.104 (0.018)	0.072	0.093	0.104	0.121	0.127

*Mean, standard deviation (SD) and distribution percentile values are based on 40 subjects except for Condition five where one subject's parameters were excluded. Units on K_p_, K_d_, K_f_, and T_d_ are Nm/rad, Nms/rad, rad/(Nms), and s, respectively. K_p_ and K_d_ values for each subject were normalized by the subject's mgh (mass × gravity × center of mass height; units Nm/rad) value*.

**Figure 5:**
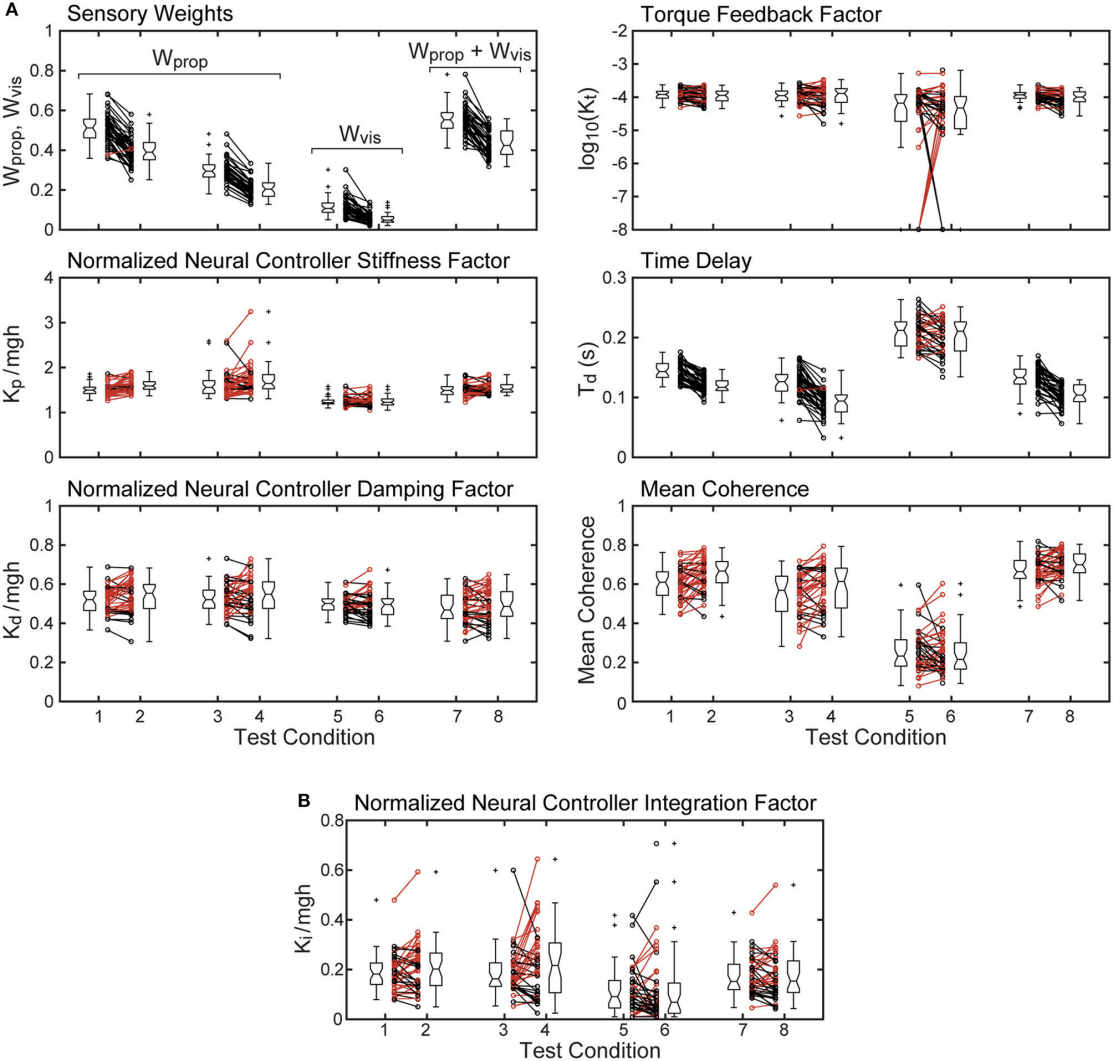
Mean coherence and identified parameter values for the 8 test conditions from 40 subjects whose stimulus-response behavior was modeled using a proportional-derivative neural controller plus torque feedback are shown in **(A)**. Subject data points from 2 and 4° stimulus-amplitude tests for the 4 test types are connected by lines with black lines and points indicating smaller parameter values on the 4° tests compared to the 2° tests and red lines indicating larger parameter values on the 4° tests. Boxplots next to the individual points show median values (center horizontal line), lower and upper 25th and 75th percentile values (lower and upper edges of the box), approximate 95% confidence limits on the median values (notches on the box), error bars (spanning smallest to largest individual values that are not considered to be outliers), and outlying data points (+'s). **(B)** shows values of the normalized integration control factor derived from model fits using a proportional-integral-derivative neural controller. Parameters from the single subject whose model fit for test condition five was not compatible with stability are not included.

Parameters and mean coherence values for all subjects on each test condition are shown in Figure 5. The figure includes boxplots that show median parameter values for each test condition and summarize the parameters distributions. The figure also shows parameter values of individual subjects.

The sensory weight measures showed consistent changes across test amplitude. In each of the 4 test types, the sensory weights on 4° trials were, on average, lower than on 2° trials of corresponding test types. This was the case for all but one individual in 1 test type. Because the model assumed that a sensory weight represents the relative contribution of a particular sensory system to overall balance control, a decrease in one weight must be associated with an increase in the contribution of a different sensory system. Specifically, for eyes closed surface-tilt tests (conditions 1 and 2 where only proprioception and vestibular cues contribute to balance), *W*_*prop*_ is the identified parameter and then the vestibular contribution is given by *W*_*vest*_ = 1−*W*_*prop*_. Vision also contributes to balance on conditions 3 and 4 so the combined contribution of visual and vestibular to balance is given by *W*_*vis*_ + *W*_*vest*_ = 1−*W*_*prop*_. The identified sensory weight in the visual stimulus conditions (conditions 5 and 6) is *W*_*vis*_ so the combined contribution of vestibular and proprioception is *W*_*vest*_ + *W*_*prop*_ = 1−*W*_*vis*_. Finally, in combined surface and visual stimulus conditions (conditions 7 and 8), the identified sensory weight is the combined visual and proprioceptive contribution *W*_*vis*_ + *W*_*prop*_, so the vestibular contribution is *W*_*vest*_ = 1−(*W*_*vis*_ + *W*_*prop*_).

The time delay parameter was consistently smaller on 4° vs. 2° amplitude tests on 3 of the 4 test types (surface-tilt eyes open and closed, and combined surface + visual tilt stimuli). On these same 3 test types, the time delays were notably smaller (mean = 0.120 s across the 3 test types) than on the visual stimulus conditions (mean = 0.206 s).

**Figure 6:**
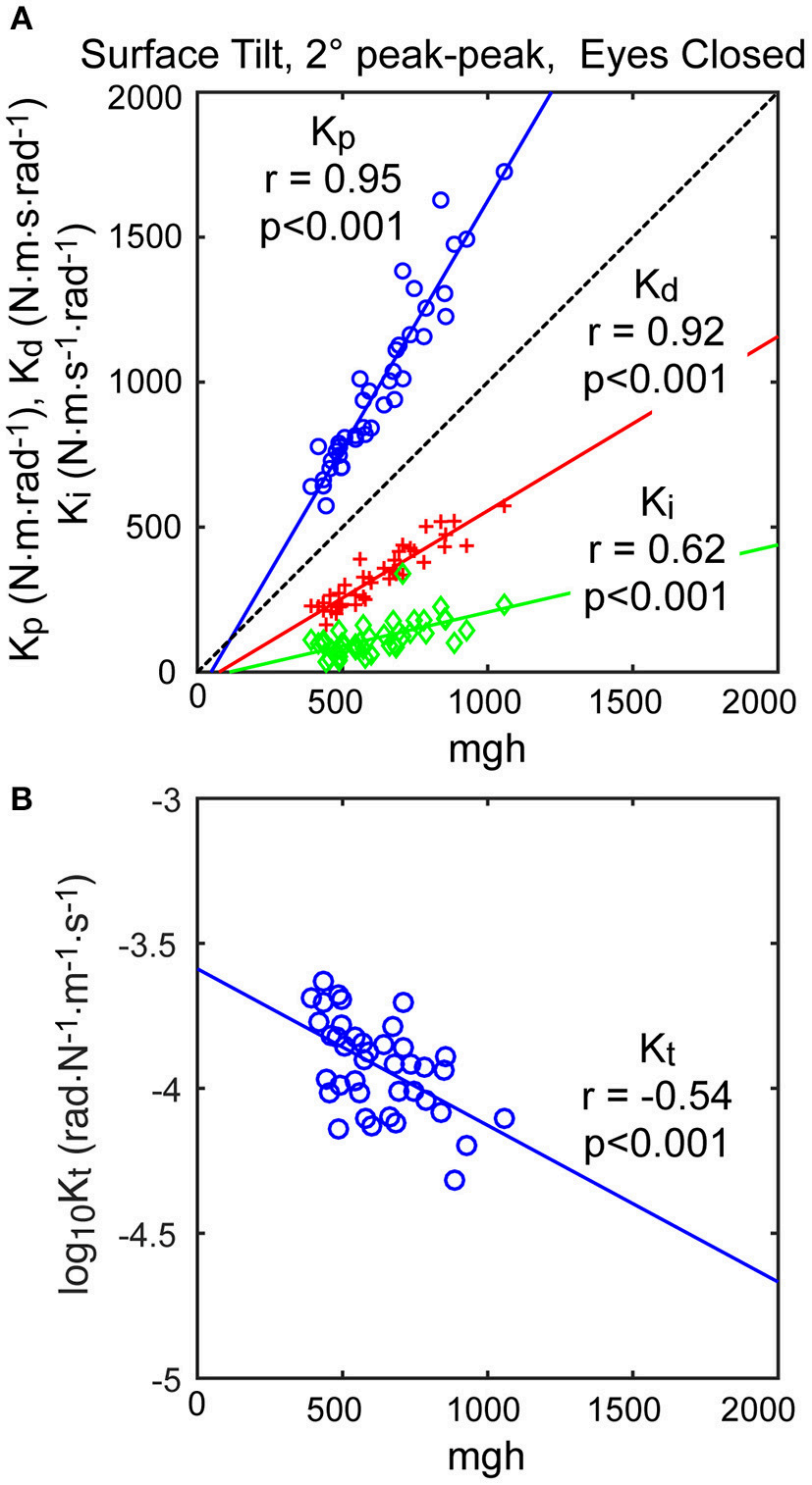
Correlation of neural controller and torque feedback parameters with *mgh* (body mass excluding feet x gravity constant × center of mass height above ankle joints). Individual values are shown for 40 subjects from the surface-tilt, eyes closed, 2° tests. In **(A)**, the *K*_*p*_ and *K*_*d*_ parameters are from the model with proportional-derivative control plus torque feedback and *K*_*i*_ is from the model with proportional-integral-derivative control. **(B)** shows a negative correlation between the torque feedback parameter *K*_*t*_ and *mgh*.

The neural controller parameters *K*_*p*_ and *K*_*d*_ shown in Figure 5 were normalized by dividing by *mgh* to account for the high correlation of these neural controller parameters with body mechanics parameters (Figure 6A). The high correlation was expected since larger subjects must generate a larger corrective torque to compensate for the larger balance disturbance caused by gravity with *mgh* being the disturbance torque due to gravity. Similarly, the damping parameter *K*_*d*_ was also highly correlated with *mgh*. The normalized *K*_*p*_ values were generally larger for the 3 test types that include surface-tilt simulation (mean = 1.58 across conditions 1–4, 7, 8) than on the visual stimulus conditions (mean = 1.24 across conditions 5, 6). Both normalized *K*_*p*_ and *K*_*d*_ values were slightly larger on the 4° tests than the 2° tests for the 3 test types that include surface-tilt stimulation.

Notably there were several outlying values of normalized *K*_*p*_ on the surface-tilt eyes open tests (conditions 3 and 4). There was no indication that these outlying values were due to poor quality parameter estimates or that these subjects had abnormal balance control systems. A possible explanation is that a few subjects under these test conditions used a strategy that maintained a stiffer balance control system by using co-contraction to increase a passive contribution to overall corrective torque production. Because our model did not separately represent a passive component, the model fit attributed the increased overall stiffness to a higher value of *K*_*p*_. Consistent with this explanation, the shortest time delay parameters identified on test conditions 3 and 4 were associated with the same subjects who had the largest normalized *K*_*p*_ values. This is consistent because torque generated by passive stiffness acts without time delay whereas, the sensorimotor contribution to torque generation has a finite time delay. Therefore, time delay values from subjects who made greater use of co-contraction would be expected to have shorter values since there was only one time-delay parameter in the model that represents an overall effective time delay. Additionally, the generally shorter time delay values on 4° vs. 2° tests across all test conditions could also be attributed a greater contribution of passive torque. However, other explanations are also plausible, such as there being different time delays associated with the different sensory contributions to balance. For example, if the time delay of the vestibular contribution to balance was shorter than other sensory systems, then an up-weighting of the vestibular contribution at the higher stimulus amplitude could also cause an apparent overall reduction in time delay.

The torque feedback parameter, *K*_*t*_, modifies the contribution of the neural controller to the generation of corrective torque. Because of the association of torque feedback with overall torque generation, one might expect that *K*_*t*_ would also scale with increasing *mgh*. *K*_*t*_ did show a weak correlation with *mgh*, however it was a negative correlation (Figure 6B). The reason for this becomes evident if the frequency dependent relationship between sensory error and overall corrective torque generation is considered. Specifically, torque feedback only affects the magnitude of torque generation at frequencies below about 0.1 Hz such that larger values of *K*_*t*_ result in a greater reduction in torque. If *K*_*p*_ and *K*_*d*_ are increased without changing *K*_*t*_, the relative influence of *K*_*t*_ increases and there is relatively less corrective torque generated below 0.1 Hz. This effect can be countered if *K*_*t*_ is decreased when *K*_*p*_ and *K*_*d*_ are increased. The net effect is that dynamic characteristics of the balance control system can remain invariant across subjects with different values of *mgh* if *K*_*t*_ is lower in subjects with larger *mgh*.

**Figure 7:**
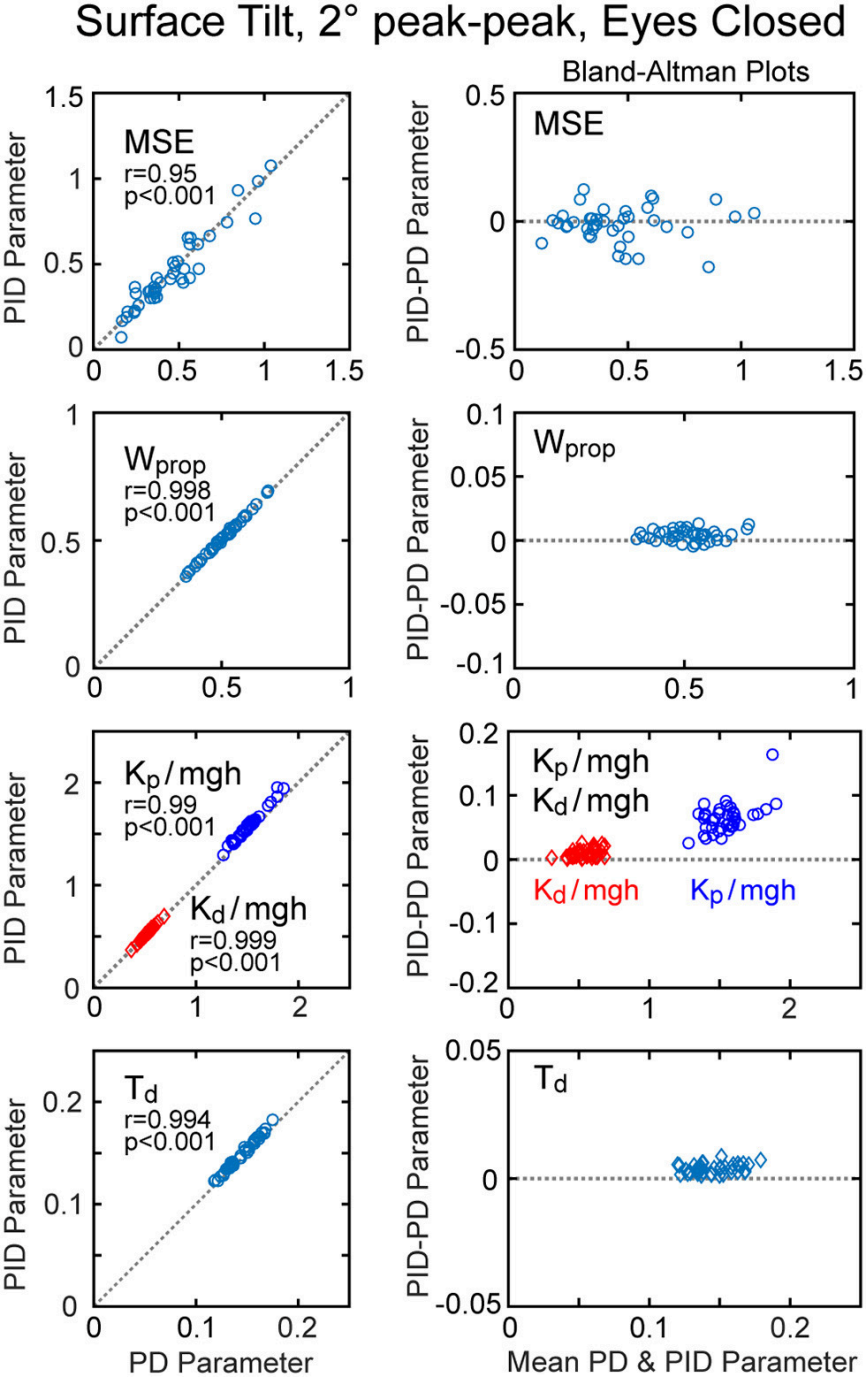
Correlation and Bland-Altman plots comparing model fit mean square error (MSE) and parameters from the model with PD (proportional-derivative control) plus torque feedback with parameters from a PID (proportional-integral-derivative) model. Comparisons are shown for results from 40 subjects on the surface-tilt, eyes closed, 2° amplitude test.

An alternative version of the balance control model with a PID neural controller rather than a PD controller with torque feedback also provided a good representation of the balance control system (parameters summarized in Supplementary Table 1). The estimates of parameters that are common to the two models were very similar and the overall MSE was nearly identical. Parameters common to the two model versions are compared in Figure 7 by showing correlation plots (left column) and Bland-Altman plots (right column). These parameter comparisons include sensory weights, neural controller parameters *K*_*p*_ and *K*_*d*_, and time delay for the two model versions for the eyes closed 2° surface-tilt condition. Although correlations were uniformly high, the Bland Altman plots reveal small biases between parameter measures from the two different models. Across all test conditions the mean differences between parameters from the model with PID control and the model with PD plus torque feedback were 0.0025, 0.059, 0.0085, and 0.0034 for sensory weight, normalized *K*_*p*_, normalized *K*_*d*_, and time delay, respectively, corresponding to percent differences of 0.77, 3.8, 1.7, and 2.4%. Positive differences and percentages indicate that the parameters from PID model were greater than from the PD plus torque feedback model. Unlike the *K*_*t*_ parameter, the integral control parameter *K*_*i*_ of the PID controller does scale with *mgh* (Figure 6A). Figure 6A plots the normalized *K*_*i*_ values of the 40 subjects for test condition 1.

Table 2 summarizes the VAF measures from the 8 test conditions and the 2 model configurations (PID and PD plus torque feedback). Both model configurations were equally effective in accounting for the experimental evoked sway. The VAF values were notably smaller on the visual stimulus conditions compared to all other conditions consistent with low sensitivity to visual stimulation (i.e., low *W*_*vis*_ values) and low coherence.

**Table 2:** Variance accounted for (VAF) measures expressed as percentages obtained from comparisons of experimental and model simulated responses to balance perturbations in the 8 test conditions.

**Test condition**	**VAF PD + torque feedback model**	**VAF PID model**
1: Surf Stim 2°, Eyes Closed	95.1 (2.9)	94.9 (3.6)
2: Surf Stim 4°, Eyes Closed	94.7 (3.2)	95.1 (4.3)
3: Surf Stim 2°, Eyes Open	94.2 (5.6)	92.4 (11.0)
4: Surf Stim 4°, Eyes Open	94.0 (5.8)	89.2 (14.1)
5: Vis Stim 2°, Eyes Open	73.2 (22.3)	74.9 (23.2)
6: Vis Stim 4°, Eyes Open	75.1 (19.9)	76.3 (20.9)
7: Surf+Vis Stim 2°, Eyes Open	97.3 (1.3)	96.2 (2.6)
8: Surf+Vis Stim 4°, Eyes Open	96.2 (2.3)	96.7 (2.2)

*Mean and standard deviation (SD) values are based on 40 subjects except for Condition five where one subject's parameters were not consistent with a stable system and were excluded*.

### Results From Lowpass Filter Estimates of CoM

**Figure 8:**
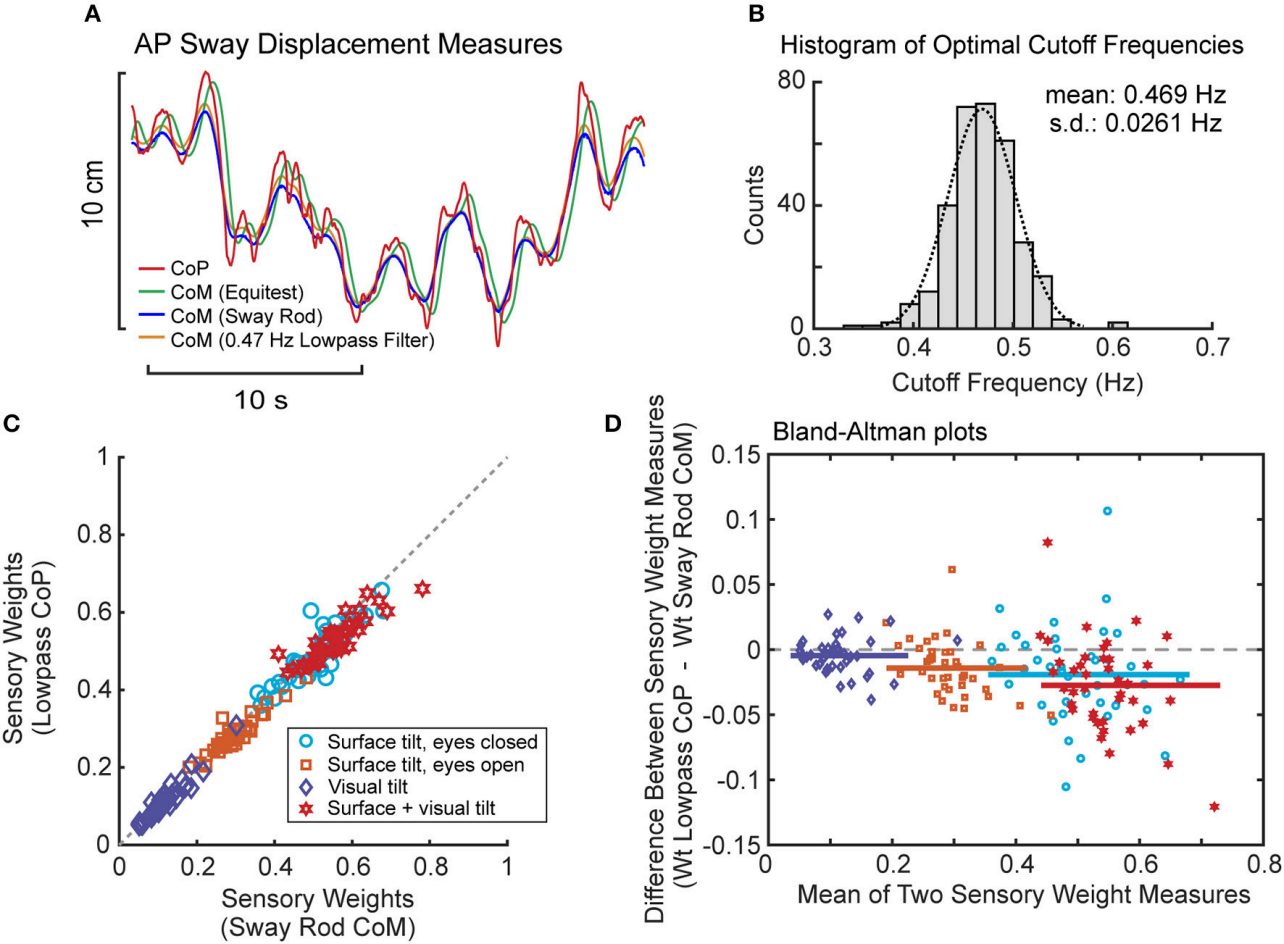
Results associated with phaseless lowpass filtering of center-of-pressure (CoP) to estimate center-of-mass (CoM) displacement. **(A)** Example traces of CoP and CoM displacement measures from EquiTest recordings, sway rod estimates, and phaseless lowpass filtering. **(B)** Distribution of cutoff frequencies that minimized errors between CoM derived from phaseless lowpass filtering and sway rod estimates. **(C)** Comparison of sensory weights based on sway rod and lowpass filtered CoP measures of CoM from 40 subjects on the 4 test conditions with 2° amplitudes. Gray dashed line shows 1:1 slope. Significance values on all comparisons in **(C)** are *p* < 0.001. **(D)** Bland Altman plots comparing the sensory weight measures shown in **(C)**.

Lowpass filtering of the recorded CoP provided a measure of CoM displacement that corresponded closely to sway rod measures of CoM displacement (Figure 8A). Across all subjects and tests, the cutoff frequency that provided the best fit to sway rod CoM was tightly distributed with mean 0.469 Hz ± 0.0261 SD (Figure 8B). This mean cutoff frequency was used to filter CoP across all tests. Then this lowpass filter derived CoM sway was used to calculate FRFs and the parameters were estimated for the model using PD plus torque feedback. There was a close correspondence between model parameters derived using CoM sway rod measures and lowpass filtering (Figures 8C,D). Across all test conditions, the mean difference between model parameters from sway rod CoM vs. lowpass CoM was −0.015, −0.048, −0.0062, 0.005, and −7.9 × 10^−6^ for sensory weight, normalized *K*_*p*_, normalized *K*_*d*_, time delay, and *K*_*t*_, respectively, corresponding to percent differences of −4.7, −3.2, −1.2, 3.5, and −7.3%. Negative differences and percentages indicate that the parameters from sway rod CoM results were greater than from lowpass CoM. Descriptive statistics of parameters derived using CoM from lowpass filtered CoP are given in the Supplemental Table S2.

### Examples From Subjects With Balance Deficits

The methods related to those described in this paper have been used to investigate how various disorders affect balance control. Here we present some examples from previous and ongoing studies that illustrate how application of CSMI testing can be used to better understand how balance control is influenced by specific deficits and to characterize mechanisms that compensate (or not) for deficits. The examples include results from subjects with bilateral vestibular loss, unilateral vestibular loss, and mTBI. An addition example demonstrates the ability of CSMI tests to identify normal balance function in an individual apparently intent on disrupting the test procedure.

**Figure 9:**
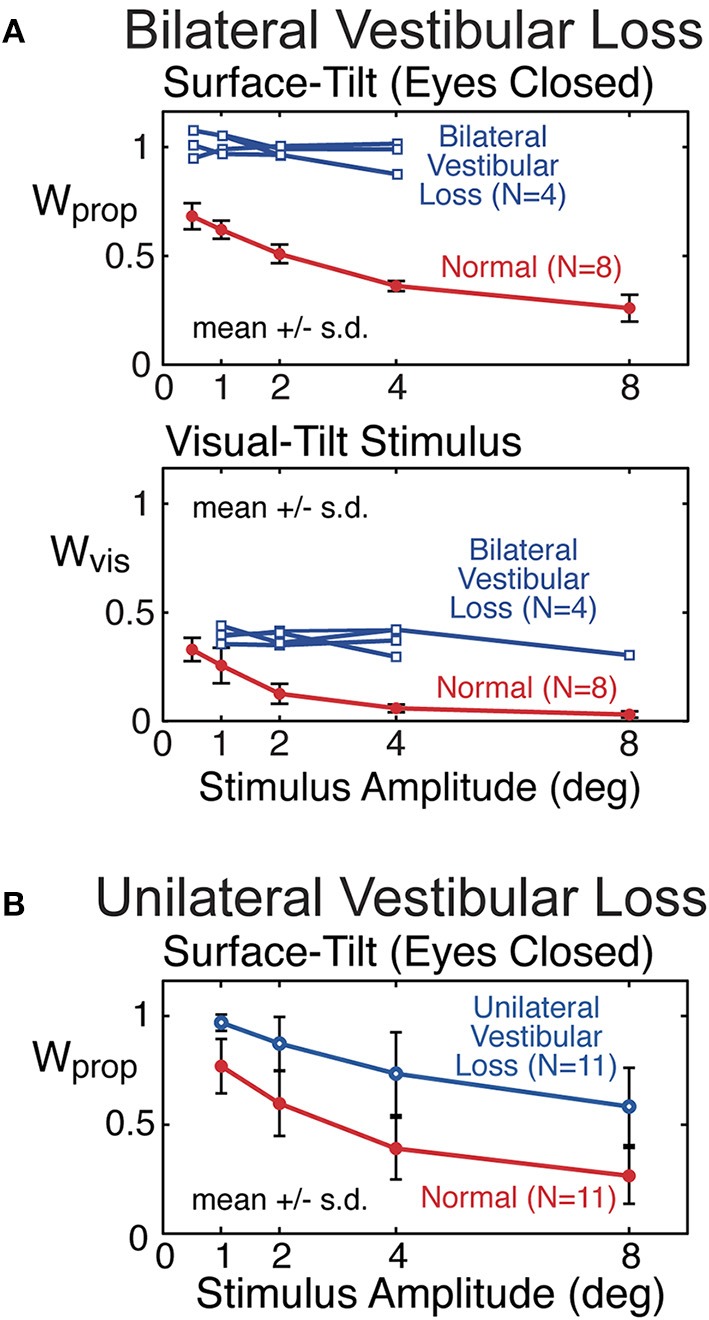
Example results from previous studies showing sensory weight measures in subjects with bilateral vestibular loss **(A)** and well compensated unilateral vestibular loss **(B)** in comparison to subjects with normal vestibular function. Bilateral vestibular loss results from Peterka (6) and unilateral loss results from Peterka (11).

Proprioceptive sensory weights from 4 subjects with severe bilateral vestibular loss tested with eyes closed using a pseudorandom surface-tilt stimulus (peak-to-peak amplitudes ranging from 0.5 to 4°) that evoked AP sway are shown in Figure 9A and are compared to mean results from 8 subjects with normal sensory function from the same study (6). The results confirm the expectation that orientation information from proprioception and the vestibular system are the primary contributors to balance control when visual cues are not available. The vestibular loss subjects compensate for the loss by becoming 100% reliant of proprioceptive information as indicated by the identified proprioceptive weights equal to unity across all stimulus amplitudes. With increasing stimulus amplitude, subjects with normal vestibular function decrease their reliance on proprioceptive information as indicated by the decrease in proprioceptive weights. The model-based interpretation of this decreasing reliance on proprioception is that subjects are increasing their reliance on vestibular cues (*W*_*vest*_ = 1−*W*_*prop*_) with increasing stimulus amplitude. The inability of vestibular loss subjects to modify their proprioceptive sensory weight with increasing stimulus amplitude confirms the model-based interpretation of the sensory integration constraint that the sum of the sensory weights of systems contributing to balance control equals unity, and confirms the ability of the CSMI methods to identify sensory weights. Additional confirmation of sensory integration assumptions has been obtained from experiments the independently perturbed the vestibular system using galvanic vestibular stimuli during eyes-closed surface-tilt stimuli (7).

Mean proprioceptive sensory weights as a function of stimulus amplitude from 11 subjects with well-compensated complete unilateral vestibular loss in comparison to results from age matched controls are shown in Figure 9B (11). Tests were performed eyes closed using a surface-tilt stimulus that evoked medial-lateral body sway. Head movements evoked by CSMI tests are of rather small magnitude compared to the range over which vestibular receptors can encode head motion. One might assume that subjects with unilateral vestibular loss could fully compensate for their loss by relying on accurate vestibular information from their functioning ear and, therefore, would give CSMI test results that are indistinguishable from controls. However, this was not the case since unilateral vestibular loss subjects showed a consistent bias toward increased reliance of proprioceptive cues. In particular, at the lowest stimulus amplitude the unilateral vestibular loss subjects resembled bilateral loss subjects in their essentially 100% reliance on proprioception. However, larger stimulus amplitudes could distinguish between unilateral and bilateral loss subjects since unilateral loss subjects were able to utilize their remaining vestibular function, although results from individual subjects showed wide variations in this ability.

**Figure 10:**
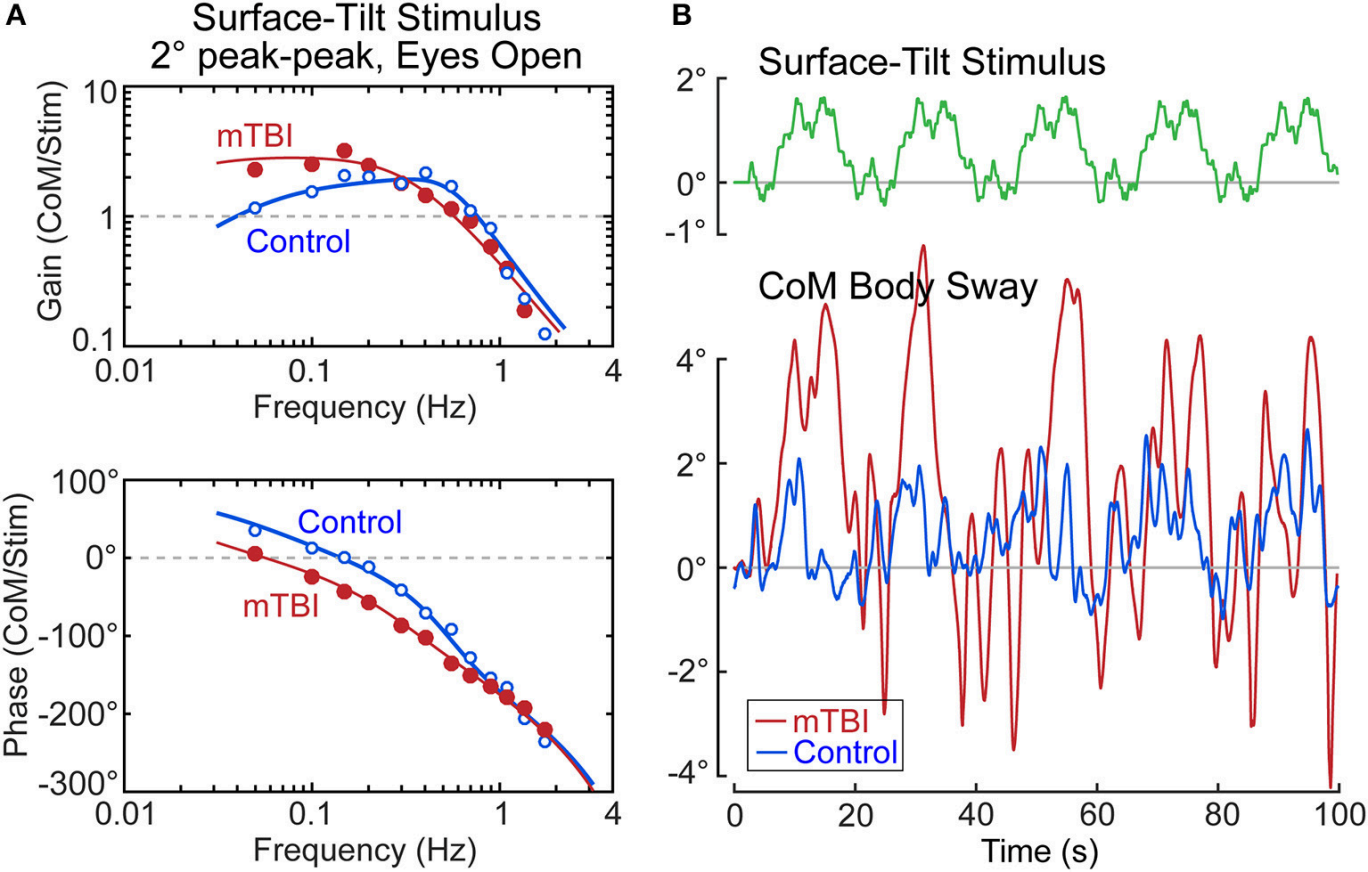
Example results from a subject with chronic balance complaints following mild traumatic brain injury (mTBI) in comparison to a control subject. Model fits (solid lines) to the frequency response function data in **(A)** identified equal sensory weight measures of *W*_*prop*_ = 0.42 for both subjects. The divergence between the mTBI and control results are accounted for by the mTBI subject having a lower value of the neural controller proportional gain parameter, *K*_*p*_, and lower value of the torque feedback parameter, *K*_*t*_. The functional consequences of these differences are that the surface-tilt stimulus evoked much larger sway in the mTBI subject **(B)**.

Our ongoing study of mTBI subjects with chronic balance complaints has identified deficits primarily in the sensory-to-motor mechanism of balance control (i.e., neural controller) in a few subjects. An example from one mTBI subject is shown in Figure 10 together with a control subject whose identified proprioceptive sensory weight on an example test (eyes open, 2° surface-tilt; condition 3) was the same as the mTBI subject (both had *W*_*prop*_ = 0.42 which were near the high end of the range for control subjects in condition 3). The FRFs of the mTBI and control subject were quite different with the mTBI subject having ~2 times larger gains in the low frequency region below ~0.15 Hz, and greater phase lags at low to mid frequencies (Figure 10A). The solid lines show that the model fits accounted well for the experimental FRF data.

The mTBI subject's increased sensitivity to the surface-tilt stimulus in comparison to the control subject was obviously not attributed to differences in sensory weights since the mTBI subject and control subject were selected to have the same sensory weight. The higher sensitivity in the mTBI subject was largely due to low neural controller stiffness (normalized *K*_*p*_ = 1.18—a value below that of all control subjects). While this value was only 13% lower than the normalized stiffness of the control subject (normalized *K*_*p*_ = 1.36), a low stiffness control has an exaggerated influence on overall response sensitivity due to the feedback nature of the balance control system. Specifically, the equations that define dynamic characteristics of the balance control system predict that the peak mid-frequency FRF gain is approximately:

(15) $$\begin{array}{l} Peak\;Gain=W_{prop}\frac{\frac{K_{p}}{mgh}}{\frac{K_{p}}{mgh}-1} \end{array}$$

Substituting the values for the mTBI subject and the control subject into this equation give peak gain values of 2.8 and 1.6, respectively, which correspond well to the peak FRF gains shown in Figure 10A. The 1.75 times greater sensitivity of the mTBI subject to the stimulus is largely attributed to the mTBI subject's reduced stiffness, but a second factor also contributed to the increasing divergence between the mTBI and control subject's FRF gain values at the lowest frequencies. Specifically, the mTBI subject's torque feedback factor (*K*_*t*_ = 2.7 × 10^−5^) was 3.8 times smaller than that of the control subject, and was smaller than all but one *K*_*t*_ value of the 40 control subjects in this study in test condition 3. The torque feedback mechanism contributes to balance control by moving the body toward an upright position (even if the surface is tilted) to reduce the overall magnitude of corrective torque generation. But this torque feedback mechanism has an influence on sway behavior only at frequencies below about 0.1 Hz and it is the mechanism that accounts for the low frequency decline in FRF gains. If *K*_*t*_ is very small, the subject's low frequency sway response to the tilting surface is determined by Equation 15. The combination of low stiffness, relatively high sensory weight, and greatly reduced torque feedback left this mTBI subject with overall poor balance control—the functional consequences of which were evident in the sway responses to the stimulus (Figure 10B). The control subject's sway was similar in magnitude to the surface-tilt stimulus, but the mTBI subject's sway was much larger with peak sway amplitudes very close to the limits of stance stability. In fact, only the first 5 cycles are shown because the mTBI subject fell later in the test.

**Figure 11:**
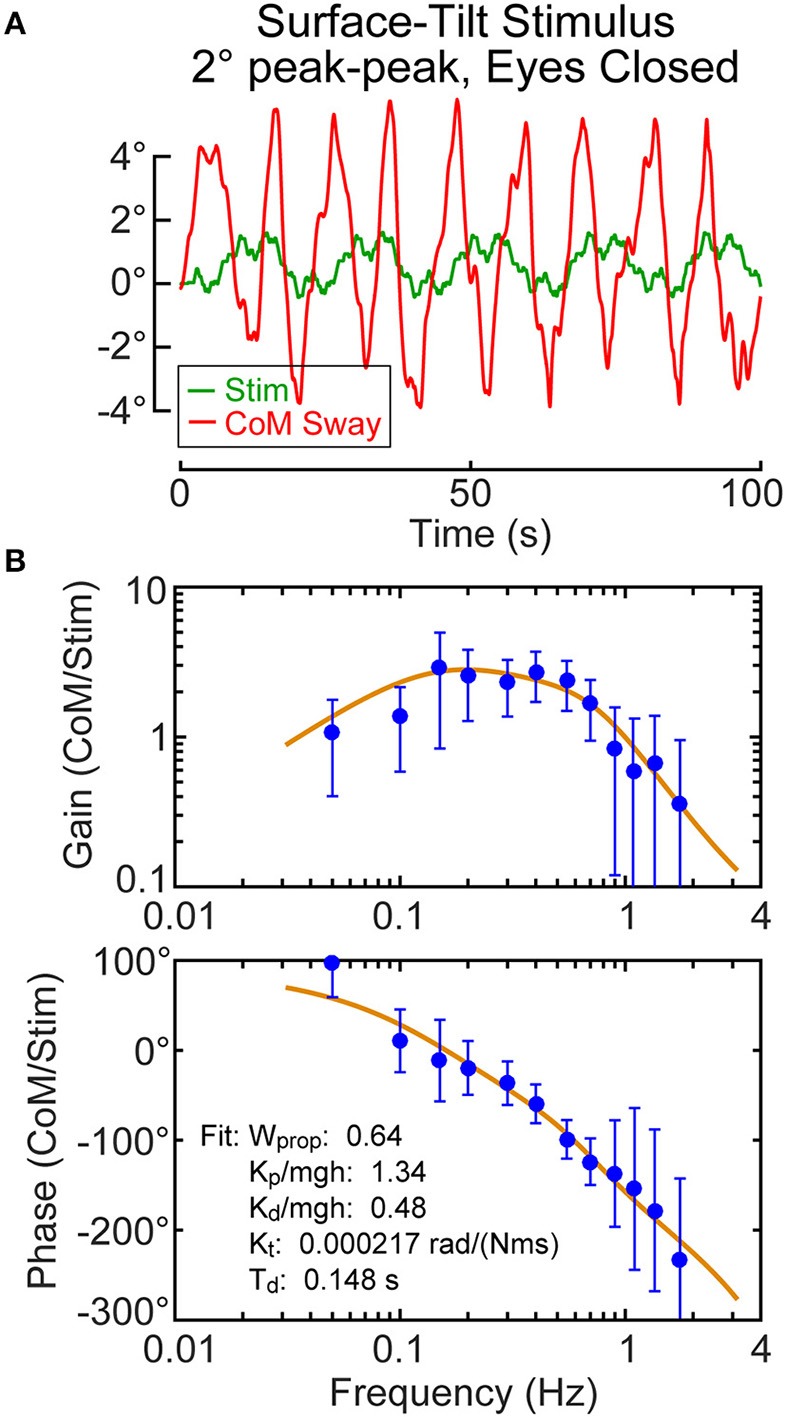
Example results from a subject with no known physiological deficits but who showed large oscillatory sway patterns **(A)**. The frequency response function data and parameters identified from the model fit **(B)** are compatible with normal balance control suggesting the possibility that the subject was purposely trying to disrupt testing.

A final example shown in Figure 11 is from a nominal control subject whose body sway showed very large oscillatory motions throughout all trials. But note that the large sway oscillations are not correlated with the stimulus. Thus, the CSMI analysis was not greatly affected by the large sway and was able to calculate FRFs from the stimulus/response data (but with reduced coherence). Parameter estimates were consistent with parameters from other control subjects. Although we cannot rule out some organic dysfunction causing this highly usual sway pattern, a plausible interpretation, based on the normal parameter measures, is that this subject was purposely interfering with the testing procedure. This subject's results were not included with the other control subjects.

## Discussion

This report provides detailed information on how to implement and interpret results from a CSMI test of balance control. Test results from 40 subjects provided sufficient information to establish preliminary normative values for parameters that characterize the normal performance of sensory integration and sensory-to-motor mechanisms contributing to balance control under a variety of test conditions. The CSMI test relies on a model-based approach to interpret body sway responses to sustained rotations of the stance surface and/or visual surround. Many practical decisions were made to successfully implement CSMI testing, but the decisions imposed limitations that are discussed below.

### Considerations and Limitations

#### Simplification of Body Mechanics

The CSMI analysis relates CoM body sway angle to a rotational stimulus that evokes that sway with the body mechanics represented by a one-segment inverted pendulum while, in reality, the body is a multi-segment system. Although methods exist to experimentally identify dynamic properties of multi-segment body systems (14, 31–33), the complexity of the identified system escalates rapidly with each added segment. If a model-based approach is used to estimate system parameters, reliability of parameter measures can suffer, and the proliferation of parameters makes the interpretation of results more complex (28).

Despite the complexity of multi-segment body motions, physics dictates that CoM must remain within the base of support for stability during sustained stance. Therefore, an analysis that focuses on CoM motion is justified. Additionally, upper and lower body segments tend to move in phase with one another at frequencies below about 1 Hz, further justifying the relevance of a one-segment body representation and focus on CoM motion if perturbing stimuli remain below about 1.5 Hz. However, if measurements of body motion are made at different segmental levels (e.g., measures of lower and upper body motion), FRFs can still be calculated relating the stimulus to body segment motion with interpretations made that mainly consider features of the FRFs, such as peak gains (13), thus bypassing a model-based interpretation.

#### Choice of Balance Control Model

We investigated two versions of the model shown in Figure 1. One used a neural controller with PD properties and torque feedback, and the other used a PID controller. An early study employed PID control (34) and a later study using methods very similar to the current study also choose PID control (6). However, this later study demonstrated that the PID control model did not account well for FRF data below about 0.05 Hz because the predicted phase of the model with PID control converges to zero degrees at very low frequencies, while actual FRF data shows low frequency phase leads. Thus, later studies favored a model based on PD control with torque feedback that does predict low frequency phase leads (7, 26). Additionally, the earlier models that used torque feedback assumed first order lowpass filter properties (i.e., a leaky integrator) for the torque feedback instead of the current model that assumes pure integration. However, the time constant of the leaky integrator was fairly long (about 8 s or more) meaning that quite low frequency FRF data is needed to obtain estimates of this time constant. Because the current study used stimulus periods of 20 s, the lowest FRF frequency of 0.05 Hz was not low enough to accurately estimate the torque feedback time constant, so a simpler pure integrator was used instead.

Neither version of the model included any parameter that represents the passive/intrinsic mechanical contribution to corrective torque generation due to muscle/tendon properties [P(s) in Figure 1]. A previous study identified passive stiffness and damping parameters that contributed 10–15% of the overall corrective torque (6) consistent with a recent study showing a relatively low contribution of passive properties under similar conditions (35). The previous study by Peterka (6) used a backboard to constrain the body to have one-segment inverted pendulum mechanics and used wider bandwidth pseudorandom stimuli. These test conditions forced a close correspondence between the model and the actual body mechanics, possibly facilitating reliable estimates of passive properties. In the current study, we investigated applying model structures that included passive stiffness and/or damping parameters but found that we were not able to reliably estimate passive properties and therefore chose to not include passive components in the final models. In particular, in models that included passive and active stiffness, the passive and active stiffness parameters could vary widely across subjects, but the sum of passive plus active stiffness parameters was typically equal to the value of the active stiffness identified using a model without passive stiffness. A recent sensitivity analysis of this type of model supports the notion that there is considerable interaction among parameters, making unique identification difficult among parameters, such as passive and active stiffness, that have similar effects on the FRFs (27). The addition of EMG recordings of leg muscles can apparently enhance the capabilities of parameter identification by making identification of passive parameters more reliable as well as allowing for identification of additional parameters related to muscle activation (36). However, for clinical applications, the additional complexity of EMG recording may not be justified unless there are specific patient populations were it would be beneficial to distinguish between passive and active contributions.

The effect of the choice to exclude a passive component is that other parameters, mainly neural controller stiffness and damping, and the time delay parameter, could be affected. That is, a simple model of passive properties could include a stiffness factor representing length-tension properties of muscles and tendons, and a damping factor representing force-velocity properties. Because these passive properties have very similar dynamic characteristics as the active neural controller parameters, except that the actively generated corrective torque is delayed in time, it is likely that parameter identification procedure would effectively include the passive contributions in the neural controller parameters. Because there is no time delay between muscle stretch and generation of passive torque, a subject whose system had a relatively large contribution from passive mechanical properties (possibly modulated by co-contraction) would likely bias the overall time delay estimate toward lower values. Indeed, a few subjects on eyes open surface tilt test (conditions 3 and 4) had large, outlying stiffness (*K*_*p*_) values (Figure 5). These were the same subjects with corresponding short time delays (*T*_*d*_) on these test conditions.

Although we were not able to reliably identify passive muscle/tendon contributions, a recent study (13) using very similar methods reported identifying passive stiffness and damping values similar to those reported in Peterka (6).

Additional motivations for investigating the two model versions were that the PID model continues to be used (13, 15) and that the PID model may be entirely adequate for quantifying and parameterizing balance control properties. Practical stimuli for clinical applications favor shorter tests. To maintain enough cycles to allow for adequate across cycle averaging, the cycle durations of the pseudorandom stimuli need to be shorter. The shorter cycle durations limit the lowest frequencies of the FRFs, and it is only at low frequencies where torque feedback provides a better accounting for FRF data than PID control. The VAF results (Table 2) indicate that both model versions can accurately represent the available data.

Results shown in Figure 7 illustrate that parameters shared by the two model versions (sensory weights, *K*_*p*_, *K*_*d*_, and *T*_*d*_) gave very similar results. However, there were some differences between parameters from the two model versions. The largest difference was in normalized *K*_*p*_ where the *K*_*p*_ values from PID model were on average 3.8% larger across all test conditions than from the PD plus torque feedback model. Differences were smaller in the other parameters but with values from the PID model always being slightly larger.

The time delay parameter was consistently identified as having a larger value in the visual stimulus conditions compared to the surface stimulus conditions (Figure 6). This could reflect an inadequacy of the Figure 1 model where only one time delay parameter is included. Effectively this assumes that time delays associated with the sensing and processing of proprioceptive, vestibular, and visual systems are all the same. The longer overall time delay identified with visual stimuli could be consistent with there being a longer time delay in visual contribution to balance control compared to other sensory systems.

#### Choice of Stimuli

For a clinical test, there is a tradeoff between test duration and accuracy/bias of estimated parameters. Bootstrap analysis was used to investigate this tradeoff using data available from the 40 participants to estimate the changes in parameter distributions and mean and median values of parameters assuming tests had included cycle averages ranging from 3 to 20 cycles (Figure 4). Parameter distributions narrowed with increasing cycles with results showing that results based on 3 cycle averages were undesirable due to larger biases in parameters estimates. In most test conditions, measures based on 6 cycle averages were likely adequate for clinical application based on reduced bias and shorter test times. However, tests based on an 11 cycle averages are a better choice for the visual tests (conditions 5 and 6) where sway responses were lower and signal-to-noise of the data were lower (see coherence results in Figure 5A).

The 4 different test types were each performed at 2 different stimulus amplitudes. The motivation for performed the same type of test at 2 amplitudes was to have a basis for identifying sensory re-weighting abnormalities as seen in previous results from bilateral vestibular loss subjects (Figure 9A). Our choice of 2 and 4° peak-to-peak stimulus amplitudes was based on concern over mechanical limitations of the EquiTest device that showed gear backlash problems in the surface rotation motor that affected stimulus repeatability, with the repeatability being poorer at lower stimulus amplitudes. In retrospect, a stimulus with an amplitude smaller than 2° would have been a better choice since a 2° stimulus can cause falls in subjects with abnormally low neural controller stiffness and torque feedback (Figure 10). Other similar recent studies have used lower amplitudes (0.5 and 1°) (13, 15, 19). Lower amplitudes have the additional potential benefit that subjects may not even perceive that their balance is being perturbed, and yet they respond reliably even to a 0.5° stimulus (6). However, longer stimulus durations should be used to avoid measurement bias due to low signal-to-noise (Figure 4).

The desire to limit the total duration of clinical testing also impacts the decision about which tests to include in a test battery. Of the 4 test types we investigated, one might argue that little additional information was gained by including both eyes-closed surface stimulation and dual surface and visual stimulation since parameters from these tests were quite similar. One might also question the utility of visual stimulus tests since results were less reliable compared to other tests. However, before deciding on a final test battery, more results are needed from patients with a broad range of pathologies to determine which tests are best able to distinguish normal from abnormal balance function.

#### Simplified Measures of Body Sway for CSMI Analysis

We investigated the possible use of a simple method for measuring AP CoM sway based on offline lowpass filtering of CoP. Brenière (22) suggested that CoM could be recovered from CoP by what amounts to appropriate symmetric (phaseless) lowpass filtering. When 0.47 Hz lowpass filter estimates of CoM were used for FRF calculations and then for model parameter estimation, there was good correspondence between parameters obtained using sway rod and lowpass filtered CoM measures. The close correspondence suggests that clinical tests can be based on the lowpass filter method for measuring CoM. However, differences were large enough that parameter norms should not be considered to be fully equivalent to those using more direct estimates of CoM motion. Nonetheless, implementation of the lowpass filter method is easier to perform, potentially making it a more practical tool for clinical use. This method reduces the overall test duration and requires less expertise in setting up and performing tests.

Also of note is that the EquiTest system provides CoM displacement measures. As shown in Figure 8A, this measure lags CoM sway rod measures and is larger in amplitude indicating that it is derived by filtering the CoP using a conventional (not a phaseless) filter with a cutoff frequency higher than our optimal 0.47 Hz cutoff frequency. Thus, FRFs derived using the EquiTest CoM measures would not provide comparable results.

### Implementation

One goal of this study was to make available a detailed explanation of the methods to perform CSMI testing. To this end, Matlab programs used to create stimuli, analyze data to obtain FRFs, and identify model parameters that optimally account for the FRF data are provided in the Supplementary Material.

## Data Availability Statement

Datasets are available on request. The raw data supporting the conclusions of this manuscript will be made available by the authors, without undue reservation, to any qualified researcher.

## Author Contributions

RP contributed to the conception and design of the study, analysis, interpretation, drafted and revised manuscript. CM contributed to analysis and interpretation of data. LP contributed to analysis, interpretation of data, and revised manuscript. PF contributed to the design of the study, data acquisition, and revised manuscript. LK contributed to the conception and design of the study, and revised manuscript. All authors approved the final manuscript.

### Conflict of Interest Statement

The authors declare that the research was conducted in the absence of any commercial or financial relationships that could be construed as a potential conflict of interest. The reviewer RK and handling Editor declared their shared affiliation at the time of the review.
