# Supplementary material for: Implementation of a Central Sensorimotor Integration Test for Characterization of Human Balance Control During Stance
Source: Front Neurol. 2018 Dec 13;9:1045. doi: 10.3389/fneur.2018.01045 (PMC6300494; doi:10.3389/fneur.2018.01045)
Supplement: Supplementary file 1 [file Data_Sheet_1.PDF]

Supplementary Material

Implementation of a Central Sensorimotor Integration Test for Characterization of Human Balance Control During Stance

Robert J. Peterka*, Charles F. Murchison, Lucy Parrington, Peter C. Fino, Laurie A. King

*** Correspondence:**

Robert Peterka: peterkar@ohsu.edu

Supplementary material includes 1) Figure 1 showing the modified EquiTest system, 2) Tables giving parameter summary statistics for models using PID control from sway-rod based measures of body sway and using PD plus torque feedback control from body sway derived from lowpass filtered center-of-pressure, and 3) Matlab programs for creation of the modified visual scene, pseudorandom stimuli used in the study, analysis of calibration tests, and analysis of sway responses to the pseudorandom stimuli.

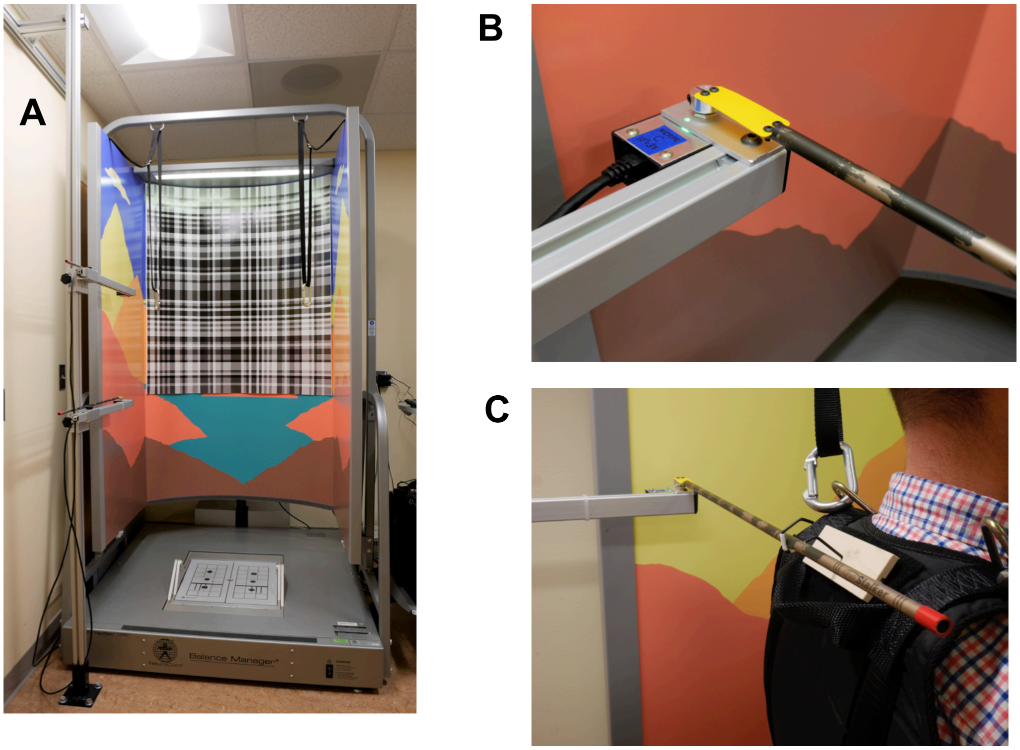

Supplementary Figure 1. Modified SMART EquiTest CRS showing floor and wall mounted frame for sway rods and visual surround insert **(A)**, detail showing flexible coupling of sway rod to a box containing a potentiometer **(B)**, and detail of one of the sway-rod hooks attached at subject's midline at shoulder height **(C)**. The sway-rod hook at hip level is attached to a Velcro belt placed at the approximate height of the greater trochanter.

Supplementary Table 1. Parameters derived using balance control model with PID neural control. Mean, standard deviation (SD) and distribution percentile values are based on 40 subjects except for Condition 5 where one subject's parameters were excluded. Units on K_p, K_d, K_i, and T_d are Nm/rad, Nms/rad, Nm/(rad s), and s, respectively. K_p, K_d, and K_i values for each subject were normalized by the subject's *mgh* (mass x gravity x center of mass height; units Nm/rad) value.

Condition	Parameter	Mean (SD)	5%tile	25%tile	50%tile	75%tile	95%tile
1	W_{prop}	0.514 (0.80)	0.373	0.465	0.518	0.557	0.685
	K_p/mgh	1.57 (0.145)	1.39	1.47	1.56	1.63	1.94
	K_d/mgh	0.524 (0.069)	0.421	0.469	0.529	0.576	0.637
	K_i/mgh	0.188 (0.075)	0.082	0.140	0.182	0.228	0.292
	T_d	0.148 (0.015)	0.123	0.137	0.145	0.161	0.173
2	W_{prop}	0.399 (0.070)	0.301	0.353	0.392	0.439	0.542
	K_p/mgh	1.67 (0.160)	1.43	1.57	1.66	1.79	1.98
	K_d/mgh	0.553 (0.088)	0.411	0.481	0.559	0.619	0.687
	K_i/mgh	0.208 (0.100)	0.081	0.134	0.202	0.270	0.349
	T_d	0.123 (0.014)	0.100	0.113	0.121	0.134	0.150
3	W_{prop}	0.304 (0.057)	0.211	0.268	0.298	0.332	0.434
	K_p/mgh	1.69 (0.313)	1.37	1.47	1.61	1.80	2.62
	K_d/mgh	0.529 (0.072)	0.403	0.482	0.525	0.570	0.642
	K_i/mgh	0.188 (0.095)	0.054	0.131	0.163	0.227	0.322
	T_d	0.129 (0.023)	0.092	0.113	0.128	0.143	0.168
4	W_{prop}	0.211 (0.047)	0.139	0.173	0.208	0.242	0.303
	K_p/mgh	1.83 (0.371)	1.48	1.59	1.78	1.96	2.84
	K_d/mgh	0.553 (0.099)	0.339	0.482	0.565	0.622	0.723
	K_i/mgh	0.233 (0.141)	0.062	0.104	0.217	0.312	0.469
	T_d	0.094 (0.022)	0.062	0.080	0.096	0.107	0.133
5	W_{vis}	0.114 (0.048)	0.051	0.082	0.104	0.130	0.215
	K_p/mgh	1.27 (0.102)	1.12	1.21	1.25	1.32	1.53
	K_d/mgh	0.503 (0.047)	0.417	0.469	0.509	0.536	0.597
	K_i/mgh	0.109 (0.096)	0.010	0.041	0.090	0.149	0.378
	T_d	0.213 (0.024)	0.172	0.191	0.214	0.230	0.260
6	W_{vis}	0.054 (0.025)	0.024	0.037	0.047	0.067	0.118
	K_p/mgh	1.27 (0.117)	1.12	1.20	1.24	1.35	1.52
	K_d/mgh	0.503 (0.072)	0.400	0.448	0.506	0.540	0.645
	K_i/mgh	0.121 (0.153)	0.010	0.023	0.069	0.148	0.544
	T_d	0.206 (0.026)	0.169	0.182	0.211	0.228	0.241
7	$W_{prop}+W_{vis}$	0.562 (0.072)	0.441	0.517	0.558	0.597	0.700
	K_p/mgh	1.55 (0.153)	1.35	1.44	1.55	1.65	1.90
	K_d/mgh	0.488 (0.080)	0.354	0.426	0.473	0.567	0.620
	K_i/mgh	0.175 (0.076)	0.086	0.118	0.152	0.227	0.311
	T_d	0.136 (0.021)	0.093	0.124	0.136	0.152	0.162
8	$W_{prop}+W_{vis}$	0.434 (0.065)	0.350	0.377	0.426	0.501	0.534
	K_p/mgh	1.60 (0.132)	1.43	1.51	1.56	1.69	1.89
	K_d/mgh	0.502 (0.089)	0.345	0.437	0.501	0.578	0.642
	K_i/mgh	0.174 (0.095)	0.048	0.107	0.153	0.236	0.312
	T_d	0.107 (0.019)	0.074	0.094	0.106	0.125	0.130

Supplementary Table 2. Parameters derived using balance control model with PD neural control plus torque feedback and using center-of-mass derived from lowpass filtering of center-of-pressure measures. Mean, standard deviation (SD) and distribution percentile values are based on 40 subjects except for Condition 5 where one subject's parameters were excluded. Units on K_p, K_d, K_f, and T_d are Nm/rad, Nms/rad, rad/(Nms), and s, respectively. K_p and K_d values for each subject were normalized by the subject's *mgh* (mass x gravity x center of mass height; units Nm/rad) value.

Condition	Parameter	Mean (SD)	5%tile	25%tile	50%tile	75%tile	95%tile
1	W_{prop}	0.490 (0.073)	0.376	0.432	0.471	0.549	0.602
	K_p/mgh	1.46 (0.117))	1.30	1.37	1.45	1.52	1.70
	K_d/mgh	0.519 (0.066)	0.430	0.468	0.515	0.562	0.619
	K_f x 10000	1.19 (0.424)	0.671	0.891	1.12	1.46	2.07
	T_d	0.153 (0.015)	0.128	0.140	0.152	0.167	0.181
2	W_{prop}	0.374 (0.070)	0.274	0.327	0.372	0.425	0.530
	K_p/mgh	1.54 (0.127)	1.35	1.45	1.51	1.62	1.78
	K_d/mgh	0.533 (0.083)	0.388	0.465	0.549	0.589	0.659
	K_f x 10000	1.10 (0.450)	0.460	0.794	0.997	1.34	2.06
	T_d	0.129 (0.013)	0.111	0.119	0.127	0.139	0.153
3	W_{prop}	0.285 (0.047)	0.204	0.257	0.278	0.302	0.384
	K_p/mgh	1.53 (0.238)	1.32	1.39	1.47	1.57	2.40
	K_d/mgh	0.524 (0.075)	0.498	0.472	0.519	0.573	0.653
	K_f x 10000	1.14 (0.422)	0.470	0.778	1.15	1.44	1.92
	T_d	0.136 (0.023)	0.096	0.120	0.136	0.150	0.173
4	W_{prop}	0.204 (0.037)	0.157	0.170	0.200	0.223	0.276
	K_p/mgh	1.65 (0.273)	1.39	1.49	1.60	1.76	2.20
	K_d/mgh	0.519 (0.094)	0.324	0.455	0.514	0.589	0.685
	K_f x 10000	1.17 (0.543)	0.329	0.724	1.16	1.55	2.15
	T_d	0.094 (0.023)	0.053	0.080	0.095	0.108	0.135
5	W_{vis}	0.113 (0.050)	0.052	0.084	0.110	0.130	0.208
	K_p/mgh	1.25 (0.099)	1.14	1.18	1.24	1.30	1.51
	K_d/mgh	0.486 (0.043)	0.408	0.454	0.491	0.513	0.562
	K_f x 10000	0.756 (0.941)	0.00	0.250	0.561	1.11	1.71
	T_d	0.204 (0.022)	0.170	0.190	0.204	0.220	0.250
6	W_{vis}	0.054 (0.027)	0.022	0.037	0.049	0.062	0.120
	K_p/mgh	1.25 (0.106)	1.11	1.19	1.23	1.31	1.48
	K_d/mgh	0.487 (0.067)	0.385	0.430	0.483	0.529	0.620
	K_f x 10000	0.862 (1.33)	0.00	0.065	0.525	0.965	05.21
	T_d	1.94 (0.025)	0.147	0.175	0.197	0.212	0.232
7	$W_{prop}+W_{vis}$	0.529 (0.054)	0.4518	0.493	0.512	0.554	0.648
	K_p/mgh	1.45 (0.119)	1.25	1.36	1.42	1.53	1.71
	K_d/mgh	0.485 (0.071)	0.357	0.437	0.478	0.530	0.617
	K_f x 10000	1.11 (0.376)	0.483	0.856	1.09	1.30	1.86
	T_d	0.143 (0.018)	0.102	0.132	0.144	0.159	0.168
8	$W_{prop}+W_{vis}$	0.403 (0.061)	0.319	0.348	0.395	0.449	0.522
	K_p/mgh	1.48 (0.105)	1.34	1.40	1.45	1.54	1.73
	K_d/mgh	0.487 (0.081)	0.343	0.430	0.487	0.545	0.615
	K_f x 10000	0.993 (0.412)	0.388	0.660	0.979	1.27	1.72
	T_d	0.117 (0.017)	0.085	0.105	0.116	0.132	0.138

Matlab programs

Example data files from calibration and pseudorandom tests are available upon request to the corresponding author.

Program for analysis of calibration trials.

```
% AnaEquiTest_Calibration_v1.m              08-Jul-2018
%
% Matlab program to analyze data collected on a calibration trial
% where a subject sways slowly forward and backward to generate a variety
% of ankle and hip joint angles. Calibration data used for subsequent
% analysis of other test trials are saved to 'Calib.mat'. The program
% reads calibration data from an ascii text file created by an export
% from EquiTest software version 8.6. A later software version 9.3
% exported unicode. Code for reading unicode and converting to ascii
% is included, but is commented out.
%
%-------------------------------------------------------------------------
% Created by Robert J. Peterka
% Email: peterkar@ohsu.edu
% Please refer to the paper: "Implementation of a Central Sensorimotor
% Integration Test for Characterization of Human Balance Control
% During Stance", R.J. Peterka, C.F. Murchison, L. Parrington, P.C. Fino, L.A. King
%
% The authors take no responsibility for use of this Matlab code.
%-------------------------------------------------------------------------
%
clear
close all

ttl='EquiTest (software v8.6) Calibration';
%
[filename, pathname] = uigetfile('*.txt','Select EquiTest Calibration file');
%
% For analysis of data collected using EquiTest software version 9.3,
% convert exported EquiTest unicode file to conventional text ascii text file
% and saved in 'temp.txt'. Then use import functions to read header data and
% test data, and later delete 'temp.txt' when done importing.
%
% fid1=fopen(filename,'rt');
% alldata=fread(fid1,'uint16');
% fclose(fid1);
% fid2=fopen('temp.txt','w');
% alldata2 = unicode2native(char(alldata), 'US-ASCII');
% fwrite(fid2, alldata2, 'uint8');
% fclose(fid2);
% [HeaderName,HeaderData] = ImportEquiTestHeader([pathname,'temp.txt']); % for EquiTest
software version 9.3

[HeaderName,HeaderData] = ImportEquiTestHeader([pathname,filename]); % for EquiTest
software version 8.6

TestDate=char(HeaderData{15});
TestTime=char(HeaderData{16});
SubjID=char(HeaderData{8});
%
% 'Comment' variable contains text that includes subject anatomic dimensions:
%       glenulo humeral joint height (inches)
%       greater trochanter height (inches)
%       knee height (inches)
%       ankle height (inches)
% in the format: 'GH52.1GT33.7KN17.5AN2.5'
```

```
% These measures, along with subject mass are used for estimate of subject
% moment-of-inertia and center-of-mass height above the ankle joint
Comment=char(HeaderData{13});
TestName=char(HeaderData{18});
ConditionName=char(HeaderData{19});
ConditionNum=str2double(char(HeaderData{20}));
TrialNum=str2double(char(HeaderData{21}));
samprate=str2double(char(HeaderData{22}));
NumPts=str2double(char(HeaderData{25}));
% 'TestComment' variable contains text that includes sway rod measures:
%       Hip sway rod height above surface (inches)
%       Shoulder sway rod height above surface (inches)
%       Hip sway rod potentiometer-to-hook length (inches)
%       Shoulder sway rod potentiometer-to-hook length (inches)
% in the format: 'HH31.8SH53.9HR18.0SR18.1'
TestComment=char(HeaderData{26});

if (ConditionNum==1)  % Calibration trial
    ETData = ImportEquiTestCalibrationData([pathname,filename]); % for EquiTest software
version 8.6
%     ETData = ImportEquiTestCalibrationData([pathname,'temp.txt']); % for EquiTest
software version 9.3
    SS=zeros(NumPts,1);
    VS=zeros(NumPts,1);
    FPLz=ETData(:,4); % N - Left force-plate total vertical force
    FPRz=ETData(:,10); % N - Right force-plate total vertical force
    CoP=ETData(:,19); % cm
    % For potentiometer angle calc scale factor is 30deg/10volts,
    %  - subtract first point (assumed to be upright position)
    hip_pot_angle=DeSpike_EquiTest((ETData(:,22)-ETData(1,22))*30/10,10,samprate);
    shoulder_pot_angle=DeSpike_EquiTest((ETData(:,23)-ETData(1,23))*30/10,10,samprate);
    t=(1:NumPts)/samprate;
    p=[strfind(Comment,'GH') strfind(Comment,'GT') strfind(Comment,'KN')
strfind(Comment,'AN')];
    GH_ht=str2double(Comment((p(1)+2):p(2)-1))*2.54; % in cm
    GT_ht=str2double(Comment((p(2)+2):p(3)-1))*2.54;
    Knee_ht=str2double(Comment((p(3)+2):p(4)-1))*2.54;
    Ankle_ht=str2double(Comment((p(4)+2):length(Comment)))*2.54;
    Mass=mean(FPLz+FPRz)/9.807   % mass in kg - assumming platform records forces in N

    [J,COM]= BodyCalc(Mass,(Knee_ht-Ankle_ht)/100,(GT_ht-Knee_ht)/100,(GH_ht-GT_ht)/100);

    p=[strfind(TestComment,'HH') strfind(TestComment,'SH') strfind(TestComment,'HR')
strfind(TestComment,'SR')];
    HipSwayRod_ht=str2double(TestComment((p(1)+2):p(2)-1))*2.54; % in cm
    ShoulderSwayRod_ht=str2double(TestComment((p(2)+2):p(3)-1))*2.54;
    HipSwayRod_length=str2double(TestComment((p(3)+2):p(4)-1))*2.54;
    ShoulderSwayRod_length=str2double(TestComment((p(4)+2):length(TestComment)))*2.54;
    %
    % Calculate hip and shoulder AP displacements
    %
    Hip_Disp=HipSwayRod_length*tand(hip_pot_angle); % AP displacement at hip pot height
    LB_angle=asind(Hip_Disp/(HipSwayRod_ht-Ankle_ht)); % lower body AP angle w.r.t.
vertical
    x_LB=Hip_Disp*(GT_ht-Ankle_ht)/(HipSwayRod_ht-Ankle_ht); % lower body AP displacement
at GT height

    Shoulder_Disp=ShoulderSwayRod_length*tand(shoulder_pot_angle); % AP displacement at
shoulder pot height
    xsp=ShoulderSwayRod_length*tand(shoulder_pot_angle); % AP displacement at shoulder pot
height
    UB_angle=asind((Shoulder_Disp-x_LB)/(ShoulderSwayRod_ht-GT_ht)); % upper body AP angle
w.r.t. vertical
    x_UB = (GH_ht-GT_ht).*sind(UB_angle) + x_LB; % UB x-displacement at GH height

    ttl=['EquiTest Calibration: ',SubjID,', ',TestDate,', ',TestTime];
```

```
    [A1,A2,OFF]=COM_CalibrationFit(t,CoP,x_LB,x_UB,LB_angle,UB_angle,ttl); % AP
calibration

    save([pathname,'/CaliData.mat'], 'A1', 'A2',
'OFF','GH_ht','GT_ht','Knee_ht','Ankle_ht','Mass','J','COM',...

'HipSwayRod_ht','ShoulderSwayRod_ht','HipSwayRod_length','ShoulderSwayRod_length');
    ['Calibration data saved to: ' pathname '/CaliData.mat']
%     delete([pathname,'temp.txt']); % delete temporary file if analyzing EquiTest v9.3
data
else
    'Not a calibration trial'
end
%%
%***************************************************************************
%
% Support functions for analysis of a calibration trial
%
%***************************************************************************
function [Source_File,ETHeader] = ImportEquiTestHeader(filename, startRow, endRow)
%
%   [SOURCE_FILE,NCHeader] =
%   ImportEquiTestHeader(FILENAME, STARTROW, ENDROW) Reads data from rows STARTROW
%   through ENDROW of text file FILENAME.
%
% Example:
%   [Source_File,NCHeader] =
%   ImportEquiTestHeader('FDf3e50178_Word_CR.txt',1, 30);
%
% Auto-generated by MATLAB on 2016/03/28 15:44:54
%
% Initialize variables.
delimiter = ' ';
if nargin<=2
    startRow = 1;
    endRow = 30;
end
% Format string for each line of text:
%   column1: text (%s)
%   column2: text (%s)
% For more information, see the TEXTSCAN documentation.
formatSpec = '%s%s%*s%*s%*s%*s%*s%*s%*s%*s%*s%*s%*s%*s%*s%*s%*s%*s%*s%*s%[^\n\r]';
% Open the text file.
fileID = fopen(filename,'r');
% Read columns of data according to format string.
% This call is based on the structure of the file used to generate this
% code. If an error occurs for a different file, try regenerating the code
% from the Import Tool.
textscan(fileID, '%[^\n\r]', startRow(1)-1, 'ReturnOnError', false);
dataArray = textscan(fileID, formatSpec, endRow(1)-startRow(1)+1, 'Delimiter', delimiter,
'MultipleDelimsAsOne', true, 'ReturnOnError', false);
for block=2:length(startRow)
    frewind(fileID);
    textscan(fileID, '%[^\n\r]', startRow(block)-1, 'ReturnOnError', false);
    dataArrayBlock = textscan(fileID, formatSpec, endRow(block)-startRow(block)+1,
'Delimiter', delimiter, 'MultipleDelimsAsOne', true, 'ReturnOnError', false);
    for col=1:length(dataArray)
        dataArray{col} = [dataArray{col};dataArrayBlock{col}];
    end
end
% Close the text file.
fclose(fileID);
% Allocate imported array to column variable names
Source_File = dataArray{:, 1};
ETHeader = dataArray{:, 2};
end
```

```
function ETData = ImportEquiTestCalibrationData(filename, startRow, endRow)
%Import numeric data from a text file as a matrix.
%   ETData = ImportEquiTestDataCalibration(FILENAME, STARTROW, ENDROW) Reads data
%   from rows STARTROW through ENDROW of text file FILENAME.
%
% Example:
%   ETData = ImportEquiTestCalibrationData('FDf3e50178MacSave.txt', 33, 24632);
%
% Auto-generated by MATLAB on 2016/03/28 14:17:06
%
% Initialize variables.
delimiter = ' ';
if nargin<=2
    startRow = 32;
    endRow = inf;
end
formatSpec = '%f%f%f%f%f%f%f%f%f%f%f%f%f%f%f%f%f%f%f%f%f%f%f%f%[^\n\r]';
% Open the text file.
fileID = fopen(filename,'r');
% Read columns of data according to format string.
% This call is based on the structure of the file used to generate this
% code. If an error occurs for a different file, try regenerating the code
% from the Import Tool.
textscan(fileID, '%[^\n\r]', startRow(1)-1, 'ReturnOnError', false);
dataArray = textscan(fileID, formatSpec, endRow(1)-startRow(1)+1, 'Delimiter', delimiter,
'MultipleDelimsAsOne', true, 'EmptyValue' ,NaN,'ReturnOnError', false);
for block=2:length(startRow)
    frewind(fileID);
    textscan(fileID, '%[^\n\r]', startRow(block)-1, 'ReturnOnError', false);
    dataArrayBlock = textscan(fileID, formatSpec, endRow(block)-startRow(block)+1,
'Delimiter', delimiter, 'MultipleDelimsAsOne', true, 'EmptyValue' ,NaN,'ReturnOnError',
false);
    for col=1:length(dataArray)
        dataArray{col} = [dataArray{col};dataArrayBlock{col}];
    end
end
% Close the text file.
fclose(fileID);
% Create output variable
ETData = [dataArray{1:end-1}];
end

function [A1,A2,OFF]=COM_CalibrationFit(t,CoP,xn_lb,xn_ub,LBangle,UBangle,ttl)
%
% Function used to determine calibration factors that relate
% AP body displacement at the hip and shoulder levels (as measured
% by sway rod potentiometers) to COM displacement (as infered by
% measures of AP COP displacement during slow body motions).
%
% Inputs:
%   t       =   time (s) - used for plotting only
%   CoP     =   AP center-of-pressure displacement data (cm)
%   xn_lb   =   AP lower body (LB) displacement at hip level (cm)
%   xn_ub   =   AP upper body (UB) displacement at shoulder level (cm)
%   LBangle =   Lower body angle w.r.t. vertical (deg) - used for plotting only
%   UBangle =   Upper body angle w.r.t. vertical (deg) - used for plotting only
%   ttl     =   title for plots
%
% Outputs:
%   A1,A2,OFF = fit parameters
%
%       COM (cm) = A1*xn_lb + A2*xn_ub + OFF
%
% LSE fit of lower and upper body displacements to CoP
AA=[sum(xn_lb.*xn_lb) sum(xn_lb.*xn_ub) sum(xn_lb)
    sum(xn_lb.*xn_ub) sum(xn_ub.*xn_ub) sum(xn_ub)
```

```
    sum(xn_lb) sum(xn_ub) length(xn_lb)];
BB=[sum(CoP.*xn_lb)
    sum(CoP.*xn_ub)
    sum(CoP)];
CC=linsolve(AA,BB);
A1=CC(1);
A2=CC(2);
OFF=CC(3);

fitCoM=A1*xn_lb+A2*xn_ub+OFF;          % best CoM fit to CoP

axis('normal')
subplot(411); plot(t,xn_lb,'b',t,xn_ub,'r');
axis([0 max(t) -20 20])
title(ttl,'Interpreter','none')
ylabel('AP LB(b), UB(r) disp (cm)')
subplot(412); plot(t,LBangle,'b',t,UBangle,'r');
axis([0 max(t) -20 20])
title('AP LB(b), UB(r) angles w.r.t. vertical'); ylabel('deg')
subplot(413); plot(t,CoP,'b',t,fitCoM,'r');
axis([0 max(t) -20 20])
title('AP CoP (b), AP CoM fit (r)'); ylabel('cm')
s='A1  = ';
s=[s,num2str(A1)];
place=0.7*max(t);
text(place,-8,s)
s='A2  = ';
s=[s,num2str(A2)];
text(place,-12,s)
s='OFF = ';
s=[s,num2str(OFF)];
text(place,-16,s)
subplot(414); plot(t,CoP-fitCoM); axis([0 max(t) -5 5])
title('Fit error: CoP - (A1*LBdisp + A2*UBdisp - OFF)'); ylabel('cm')
xlabel('Time (s)')
end

function xout=DeSpike_EquiTest(xin,thresh,rate)
%   DeSpike_EquiTest.m           15-May-16
%
%   Program to remove single sample noise spikes from sway traces
%   recorded on EquiTest platform. Infrequent spikes apparently due
%   to EquiTest instrumentation problems when digitizing signals on
%   auxilliary A/D inputs channels
%
%   xin = recorded pot angle time series (deg)
%   thres = velocity threshold for detecting spikes (deg/s)
%   rate = sampling rate of xin (samples/s)
%   xout = de-spiked time series (deg)
xout=xin;
xind=cdiff(xin)*rate;
for i=2:length(xin)-1
    if (abs(xind(i-1))>thresh)&&(abs(xind(i+1))>thresh)
        xout(i)=(xout(i-1)+xout(i+1))/2;
    end
end
end

function x = cdiff(x)
%
%CDIFF  Central Difference function.  If X is a vector
% [x(1) x(2) ... x(n)], then CDIFF(X) returns a vector
% of central differences between every second element
% [x(2)-x(1)  (x(3)-x(1))/2  (x(4)-x(2))/2  ...
%  (x(n)-x(n-2))/2  x(n)-x(n-1)].
%
```

```
% For time series, divide result x by deltat for proper
% velocity scaling where deltat is time interval between
% adjacent points x(i) and x(i+1)
%
% If X is a matrix, the differences are calculated down
% each column.
%
% The first and last elements in CDIFF are the simple
% differences between adjacent elements, and the returned
% vector or matrix has the same dimensions as the original.
    [m,n] = size(x);
    if m == 1
        y = x(3:n) - x(1:n-2);
  x = [x(2)-x(1) y./2 x(n)-x(n-1)];
    else
        y = x(3:m,:) - x(1:m-2,:);
  x = [x(2,:)-x(1,:); y./2; x(m,:)-x(m-1,:)];
    end
end

function [J,COM]= BodyCalc(Mass_kg, Len_L_m, Len_T_m, Len_HAT_m)
%
% [J,COM]= BodyCalc(Mass, Len_L, Len_T, Len_HAT)
%
% Program that calculates the Moment of Inertia (J) and
% Center of Mass at the ankle joint for platform subjects.
%
% Enter Mass in kg, and Length's in meters (e.g., Len_HAT = .545)
%
% Mass = total body mass (kg)
% Len_L_m = leg length (medial malleolus to femoral condyles in meters)
% Len_T_m = thigh length (femoral condyles to greater trochanter in meters)
% Len_HAT_m = head, arms, trunk length (greater trochanter to glenohumeral
%   joint in meters - Note that even though this length measure does not include the
%   head, the calculation of COM is based on this length measure)
%
% J = moment of inertia of the legs+thighs+HAT segments about the ankle
%       joint axis (kg-m^2)
% COM = center of mass height (meters) above the ankle joint axis of the
%       legs+thighs+HAT segments
%
% Calculation based on anthropometric relationships from:
% D.A. Winter, Biomechanics and Motor Control of Human Movement.
% New York: John Wiley & Sons, Inc., 2005.
%
Mass_HAT=0.678*Mass_kg;
Mass_T=2*0.100*Mass_kg;
Mass_L=2*0.0465*Mass_kg;
J_HAT=Mass_HAT*(Len_HAT_m*0.496)^2;
J_HAT_ankle=J_HAT +Mass_HAT*(Len_L_m+Len_T_m + 0.626*Len_HAT_m)^2;
J_T=Mass_T*(Len_T_m*0.323)^2;
J_T_ankle=J_T+Mass_T*(Len_L_m+0.567*Len_T_m)^2;
J_L=Mass_L*(Len_L_m*0.302)^2;
J_L_ankle=J_L+Mass_L*(Len_L_m*0.567)^2;
J=J_HAT_ankle + J_T_ankle + J_L_ankle;

% Center of Mass of Legs+Thighs+HAT above ankle joint
COM_HAT = 0.626*Len_HAT_m + Len_L_m + Len_T_m;
COM_T = 0.567*Len_T_m + Len_L_m;
COM_L = 0.567*Len_L_m;
COM = (Mass_HAT*COM_HAT + Mass_T*COM_T + Mass_L*COM_L) / (Mass_HAT + Mass_L + Mass_T);
end
```

Matlab program for analysis of responses to pseudorandom stimuli.

```
% AnaEquiTest_Pseudorandom_v1.m              08-Jul-2018
%
% Before running a pseudorandom analysis, a calibration test must have been
% previously analyzed and the calibraiton information in 'CaliData.mat'
% must be saved in the folder with this subject's data files.
%
% Requires access to Matlab Optimization Toolbox for curve fit to frequency
% response function for parameter estimation.
%
% Three model fits are performed with results saved in mat files:
%   Fit0: Model with PID neural controller
%   Fit1: Model with PD neural controller with Torque Feedback
%   Fit1_LP: Model with PD neural controller with Torque Feedback using CoM
%     sway data derived from lowpass filtering of CoP
%
%-----------------------------------------------------------------------
% Created by Robert J. Peterka
% Email: peterkar@ohsu.edu
% Please refer to the paper: "Implementation of a Central Sensorimotor
% Integration Test for Characterization of Human Balance Control
% During Stance", R.J. Peterka, C.F. Murchison, L. Parrington, P.C. Fino, L.A. King
%
% The authors take no responsibility for use of this Matlab code.
%-----------------------------------------------------------------------
%
clear
close all

[filename, pathname] = uigetfile('*.txt','Select EquiTest Pseudorandom file');

%
% For analysis of data collected using EquiTest software version 9.3,
% convert exported EquiTest unicode file to conventional text ascii text file
% and saved in 'temp.txt'. Then use import functions to read header data and
% test data, and later delete 'temp.txt' when done importing.
%
% fid1=fopen(filename,'rt');
% alldata=fread(fid1,'uint16');
% fclose(fid1);
% fid2=fopen('temp.txt','w');
% alldata2 = unicode2native(char(alldata), 'US-ASCII');
% fwrite(fid2, alldata2, 'uint8');
% fclose(fid2);
% [HeaderName,HeaderData] = ImportEquiTestHeader([pathname,'temp.txt']); % for EquiTest
software version 9.3

[HeaderName,HeaderData] = ImportEquiTestHeader([pathname,filename]); % for EquiTest
software version 8.6

TestDate=char(HeaderData{15});
TestTime=char(HeaderData{16});
SubjID=char(HeaderData{8});
Comment=char(HeaderData{13});
TestName=char(HeaderData{18});
ConditionName=char(HeaderData{19});
ConditionNum=str2double(char(HeaderData{20}));
TrialNum=str2double(char(HeaderData{21}));
samprate=str2double(char(HeaderData{22}));
NumPts=str2double(char(HeaderData{25}));
TestComment=char(HeaderData{26});
```

```
% Load calibration data file
load([pathname,'/CaliData.mat']);% read calibration info
mgh=(Mass-Mass*0.025)*COM*9.807; % subtract mass of feet

% Coefficients for phaseless LPF to estimate CoM displacement from CoP
[B,A]=butter(1,0.47/(samprate/2));   % 0.47 Hz LP

Eyes='EO';
Test=['Cond_',num2str(ConditionNum)];
if (ConditionNum<5)  % SS stim
    StimType='SS';
    if ConditionNum<3; Eyes='EC'; end
    ETData = ImportEquiTestData_SingleStim([pathname,filename],33,NumPts+32);
    %     ETData = ImportEquiTestData_SingleStim([pathname,'temp.txt'],33,NumPts+32);
    SS=ETData(:,2); % surface tilt angle (deg)
    VS=zeros(size(SS));
    CoP=ETData(:,20); % cm
    CoM_LP=filtfilt(B,A,CoP); % cm
    %
    % hip and shoulder potentiometer voltages in ETData(:,23) and ETData(:,24),
respectively
    %
    % potentiometer scale factor = 30deg/10volts, subtract first pt (assume to be zero
position)
    hip_pot_angle=DeSpike_EquiTest((ETData(:,23)-ETData(1,23))*30/10,10,samprate);
    shoulder_pot_angle=DeSpike_EquiTest((ETData(:,24)-ETData(1,24))*30/10,10,samprate);
elseif ((ConditionNum==5)||(ConditionNum==6))  % VS stim
    StimType='VS';
    ETData = ImportEquiTestData_SingleStim([pathname,filename],33,NumPts+32);
    %     ETData = ImportEquiTestData_SingleStim([pathname,'temp.txt'],33,NumPts+32);
    VS=ETData(:,2); % visual surround tilt angle (deg)
    SS=zeros(size(VS));
    CoP=ETData(:,20); % cm
    CoM_LP=filtfilt(B,A,CoP); % cm
    hip_pot_angle=DeSpike_EquiTest((ETData(:,23)-ETData(1,23))*30/10,10,100);
    shoulder_pot_angle=DeSpike_EquiTest((ETData(:,24)-ETData(1,24))*30/10,10,100);
elseif (ConditionNum>6)  % Dual SS and VS stim
    StimType='SS+VS';
    ETData = ImportEquiTestData_DualStim([pathname,filename],34,NumPts+33);
    %     ETData = ImportEquiTestData_DualStim([pathname,'temp.txt'],34,NumPts+33);
    SS=ETData(:,2); % surface tilt angle (deg)
    VS=ETData(:,3); % visual surround tilt angle (deg)
    CoP=ETData(:,21); % cm
    CoM_LP=filtfilt(B,A,CoP); % cm
    hip_pot_angle=DeSpike_EquiTest((ETData(:,24)-ETData(1,24))*30/10,10,100);
    shoulder_pot_angle=DeSpike_EquiTest((ETData(:,25)-ETData(1,25))*30/10,10,100);
end
%
% calculate hip and shoulder displacements,COM displacement and COM angle
%
Hip_Disp=HipSwayRod_length*tand(hip_pot_angle); % AP displacement at hip pot height
LB_angle=asind(Hip_Disp/(HipSwayRod_ht-Ankle_ht)); % lower body AP angle w.r.t. vertical
x_LB=Hip_Disp*(GT_ht-Ankle_ht)/(HipSwayRod_ht-Ankle_ht); % lower body AP displacement at
GT height

Shoulder_Disp=ShoulderSwayRod_length*tand(shoulder_pot_angle); % AP displacement at
shoulder pot height
xsp=ShoulderSwayRod_length*tand(shoulder_pot_angle); % AP displacement at shoulder pot
height
UB_angle=asind((Shoulder_Disp-x_LB)/(ShoulderSwayRod_ht-GT_ht)); % upper body AP angle
w.r.t. vertical
x_UB = (GH_ht-GT_ht).*sind(UB_angle) + x_LB; % UB x-displacement at GH height

COM_disp=A1*x_LB+A2*x_UB+OFF; % CoM displacement in cm
COM_angle=180/pi*atan((COM_disp/100)./COM); % CoM sway angle (deg)
COM_LPangle=180/pi*atan((CoM_LP/100)./COM); % COM angle from filtfilt LP of CoP
```

```
displacement

%delete('temp.txt');      % delete temporary file when analyzing data from EquiTest
software version 9.3

t=(1:NumPts)/samprate;
figure
% plot SS,VS tilt angles (deg), CoMangle (deg)
subplot(211); plot(t,SS-mean(SS(1:samprate)),'b',t,VS-
mean(VS(1:samprate)),'g',t,COM_angle,'r')
title('Surface tilt(b), Visual surround tilt(g), CoM angle(r) in degrees')
ylabel('deg')
% plot CoMdisp, CoM_LPdisp, CoP (cm)
subplot(212); plot(t,CoP-mean(CoP(1:samprate)),'r',t,COM_disp-
mean(COM_disp(1:samprate)),'b',t,CoM_LP-mean(CoM_LP(1:samprate)),'g')
title('CoM disp(b), Lowpass CoM disp(g), CoP disp(r)')
ylabel('cm'); xlabel('Time (s)')
%
% Calulate stim/resp results between stimulus and CoM angle
%
Amp_pp=2;      % ideal peak-to-peak amplitude of the stimulus
if (ConditionNum==2)||(ConditionNum==4)||(ConditionNum==6)||(ConditionNum==8)
    Amp_pp=4;
end
ppc=2000;      % points per stimulus cycle
startidx=201; % start index of first stimulus cycle
fmax=100;      % number of frequency points to calculate in the discrete Fourier transform
Maxf=12;       % number of smoothed frequency points to include in model fit procedure

ttl1=['EquiTest: ',SubjID,', ',TestDate,', ',TestTime];
ttl2=['Condition ',num2str(ConditionNum),': ',StimType,' ',num2str(Amp_pp),' deg, ',Eyes];

if ConditionNum<5  % SS stim
    SSstim=1; VSstim=0;
    % Analysis using CoM calculated from sway rod data
    [FD_SS,TD_SS] = FRFanalysis_v1(SS',COM_angle',startidx,ppc,fmax,samprate);
    [~]=FRFplotResults_v1(FD_SS,TD_SS,ppc,samprate,'SS ','CoM',ttl1,ttl2);
    StimRemnant=sqrt(FD_SS.f(1)*sum(FD_SS.yi_var));
    SwayRemnant=sqrt(FD_SS.f(1)*sum(FD_SS.yo_var));
    StimRMS=rms(TD_SS.avg_Stim-mean(TD_SS.avg_Stim));
    SwayRMS=rms(TD_SS.avg_Resp-mean(TD_SS.avg_Resp));
    MeanCoh=mean(FD_SS.Cohds(1:Maxf));
    % Fit0 is for PID fit
    [Fit0,mse0] = FRF_Fit0_and_plot_v1(FD_SS.FRFds(1:Maxf),FD_SS.fds(1:Maxf),...
        FD_SS.Cohds(1:Maxf),FD_SS.r(1:Maxf),TD_SS.avg_Stim,TD_SS.avg_Resp...
        ,TD_SS.cl95,ppc,ttl1,ttl2,J,Mass,COM,mgh,SSstim,VSstim,...
        StimRMS,SwayRMS,StimRemnant,SwayRemnant,MeanCoh,samprate);
    % Fit1 is for PD + Torque Feedback fit
    [Fit1,mse1] = FRF_Fit1_and_plot_v1(FD_SS.FRFds(1:Maxf),FD_SS.fds(1:Maxf),...
        FD_SS.Cohds(1:Maxf),FD_SS.r(1:Maxf),TD_SS.avg_Stim,TD_SS.avg_Resp...
        ,TD_SS.cl95,ppc,ttl1,ttl2,J,Mass,COM,mgh,SSstim,VSstim,...
        StimRMS,SwayRMS,StimRemnant,SwayRemnant,MeanCoh,samprate);
    % Analysis using CoM calculated from 0.047 Hz lowpass filtered CoP data
    ttl3=[ttl2,', LP CoM'];
    [FD_SS_LP,TD_SS_LP] = FRFanalysis_v1(SS',COM_LPangle',startidx,ppc,fmax,samprate);
    [~]=FRFplotResults_v1(FD_SS_LP,TD_SS_LP,ppc,samprate,'SS ','CoM_LP',ttl1,ttl3);
    StimRemnant_LP=sqrt(FD_SS_LP.f(1)*sum(FD_SS_LP.yi_var));
    SwayRemnant_LP=sqrt(FD_SS_LP.f(1)*sum(FD_SS_LP.yo_var));
    StimRMS_LP=rms(TD_SS_LP.avg_Stim-mean(TD_SS_LP.avg_Stim));
    SwayRMS_LP=rms(TD_SS_LP.avg_Resp-mean(TD_SS_LP.avg_Resp));
    MeanCoh_LP=mean(FD_SS_LP.Cohds(1:Maxf));
    % Fit1_LP is for PD + Torque Feedback fit using lowpass filtered CoP
    [Fit1_LP,mse1_LP] =
FRF_Fit1_and_plot_v1(FD_SS_LP.FRFds(1:Maxf),FD_SS_LP.fds(1:Maxf),...
        FD_SS_LP.Cohds(1:Maxf),FD_SS_LP.r(1:Maxf),TD_SS_LP.avg_Stim,TD_SS_LP.avg_Resp...
        ,TD_SS_LP.cl95,ppc,ttl1,ttl3,J,Mass,COM,mgh,SSstim,VSstim,...
```

```
        StimRMS_LP,SwayRMS_LP,StimRemnant_LP,SwayRemnant_LP,MeanCoh_LP,samprate);
elseif (ConditionNum==5)||(ConditionNum==6)  % VS stim
    SSstim=0; VSstim=1;
    % Analysis using CoM calculated from sway rod data
    [FD_VS,TD_VS] = FRFanalysis_v1(VS',COM_angle',startidx,ppc,fmax,samprate);
    [~]=FRFplotResults_v1(FD_VS,TD_VS,ppc,samprate,'VS ','CoM',ttl1,ttl2);
    StimRemnant=sqrt(FD_VS.f(1)*sum(FD_VS.yi_var));
    SwayRemnant=sqrt(FD_VS.f(1)*sum(FD_VS.yo_var));
    StimRMS=rms(TD_VS.avg_Stim-mean(TD_VS.avg_Stim));
    SwayRMS=rms(TD_VS.avg_Resp-mean(TD_VS.avg_Resp));
    MeanCoh=mean(FD_VS.Cohds(1:Maxf));
    % Fit0 is for PID fit
    [Fit0,mse0] = FRF_Fit0_and_plot_v1(FD_VS.FRFds(1:Maxf),FD_VS.fds(1:Maxf),...
        FD_VS.Cohds(1:Maxf),FD_VS.r(1:Maxf),TD_VS.avg_Stim,TD_VS.avg_Resp...
        ,TD_VS.cl95,ppc,ttl1,ttl2,J,Mass,COM,mgh,SSstim,VSstim,...
        StimRMS,SwayRMS,StimRemnant,SwayRemnant,MeanCoh,samprate);
    % Fit1 is for PD + Torque Feedback fit
    [Fit1,mse1] = FRF_Fit1_and_plot_v1(FD_VS.FRFds(1:Maxf),FD_VS.fds(1:Maxf),...
        FD_VS.Cohds(1:Maxf),FD_VS.r(1:Maxf),TD_VS.avg_Stim,TD_VS.avg_Resp...
        ,TD_VS.cl95,ppc,ttl1,ttl2,J,Mass,COM,mgh,SSstim,VSstim,...
        StimRMS,SwayRMS,StimRemnant,SwayRemnant,MeanCoh,samprate);
    % Analysis using CoM calculated from 0.047 Hz lowpass filtered CoP data
    ttl3=[ttl2,', LP CoM'];
    [FD_VS_LP,TD_VS_LP] = FRFanalysis_v1(VS',COM_LPangle',startidx,ppc,fmax,samprate);
    [~]=FRFplotResults_v1(FD_VS_LP,TD_VS_LP,ppc,samprate,'VS ','CoM_LP',ttl1,ttl3);
    StimRemnant_LP=sqrt(FD_VS_LP.f(1)*sum(FD_VS_LP.yi_var));
    SwayRemnant_LP=sqrt(FD_VS_LP.f(1)*sum(FD_VS_LP.yo_var));
    StimRMS_LP=rms(TD_VS_LP.avg_Stim-mean(TD_VS_LP.avg_Stim));
    SwayRMS_LP=rms(TD_VS_LP.avg_Resp-mean(TD_VS_LP.avg_Resp));
    MeanCoh_LP=mean(FD_VS_LP.Cohds(1:Maxf));
    % Fit1_LP is for PD + Torque Feedback fit using lowpass filtered CoP
    [Fit1_LP,mse1_LP] =
FRF_Fit1_and_plot_v1(FD_VS_LP.FRFds(1:Maxf),FD_VS_LP.fds(1:Maxf),...
        FD_VS_LP.Cohds(1:Maxf),FD_VS_LP.r(1:Maxf),TD_VS_LP.avg_Stim,TD_VS_LP.avg_Resp...
        ,TD_VS_LP.cl95,ppc,ttl1,ttl3,J,Mass,COM,mgh,SSstim,VSstim,...
        StimRMS_LP,SwayRMS_LP,StimRemnant_LP,SwayRemnant_LP,MeanCoh_LP,samprate);
elseif (ConditionNum==7)||(ConditionNum==8)  % Dual SS and VS stim
    SSstim=1; VSstim=1;
    % Surface analysis
    % Analysis using CoM calculated from sway rod data
    [FD_SS,TD_SS] = FRFanalysis_v1(SS',COM_angle',startidx,ppc,fmax,samprate);
    [~]=FRFplotResults_v1(FD_SS,TD_SS,ppc,samprate,'SS ','CoM',ttl1,ttl2);

    StimRemnant_SS=sqrt(FD_SS.f(1)*sum(FD_SS.yi_var));
    SwayRemnant_SS=sqrt(FD_SS.f(1)*sum(FD_SS.yo_var));
    StimRMS_SS=rms(TD_SS.avg_Stim-mean(TD_SS.avg_Stim));
    SwayRMS_SS=rms(TD_SS.avg_Resp-mean(TD_SS.avg_Resp));
    MeanCoh_SS=mean(FD_SS.Cohds(1:Maxf));
    % Fit0 is for PID fit
    ttl3=[ttl2,', SS analysis'];
    [Fit0_SS,mse0_SS] = FRF_Fit0_and_plot_v1(FD_SS.FRFds(1:Maxf),FD_SS.fds(1:Maxf),...
        FD_SS.Cohds(1:Maxf),FD_SS.r(1:Maxf),TD_SS.avg_Stim,TD_SS.avg_Resp...
        ,TD_SS.cl95,ppc,ttl1,ttl3,J,Mass,COM,mgh,SSstim,VSstim,...
        StimRMS_SS,SwayRMS_SS,StimRemnant_SS,SwayRemnant_SS,MeanCoh_SS,samprate);
    % Fit1 is for PD + Torque Feedback fit
    [Fit1_SS,mse1_SS] = FRF_Fit1_and_plot_v1(FD_SS.FRFds(1:Maxf),FD_SS.fds(1:Maxf),...
        FD_SS.Cohds(1:Maxf),FD_SS.r(1:Maxf),TD_SS.avg_Stim,TD_SS.avg_Resp...
        ,TD_SS.cl95,ppc,ttl1,ttl3,J,Mass,COM,mgh,SSstim,VSstim,...
        StimRMS_SS,SwayRMS_SS,StimRemnant_SS,SwayRemnant_SS,MeanCoh_SS,samprate);
    % Analysis using CoM calculated from 0.047 Hz lowpass filtered CoP data
    ttl3=[ttl2,', LP CoM, SS analysis'];
    [FD_SS_LP,TD_SS_LP] = FRFanalysis_v1(SS',COM_LPangle',startidx,ppc,fmax,samprate);
    [~]=FRFplotResults_v1(FD_SS_LP,TD_SS_LP,ppc,samprate,'SS ','CoM_LP',ttl1,ttl2);
    StimRemnant_LP_SS=sqrt(FD_SS_LP.f(1)*sum(FD_SS_LP.yi_var));
    SwayRemnant_LP_SS=sqrt(FD_SS_LP.f(1)*sum(FD_SS_LP.yo_var));
    StimRMS_LP_SS=rms(TD_SS_LP.avg_Stim-mean(TD_SS_LP.avg_Stim));
    SwayRMS_LP_SS=rms(TD_SS_LP.avg_Resp-mean(TD_SS_LP.avg_Resp));
```

```
    MeanCoh_LP_SS=mean(FD_SS_LP.Cohds(1:Maxf));
    % Fit1_LP is for PD + Torque Feedback fit using lowpass filtered CoP
    [Fit1_LP_SS,mse1_LP_SS] =
FRF_Fit1_and_plot_v1(FD_SS_LP.FRFds(1:Maxf),FD_SS_LP.fds(1:Maxf),...
        FD_SS_LP.Cohds(1:Maxf),FD_SS_LP.r(1:Maxf),TD_SS_LP.avg_Stim,TD_SS_LP.avg_Resp...
        ,TD_SS_LP.cl95,ppc,ttl1,ttl3,J,Mass,COM,mgh,SSstim,VSstim,...

StimRMS_LP_SS,SwayRMS_LP_SS,StimRemnant_LP_SS,SwayRemnant_LP_SS,MeanCoh_LP_SS,samprate);

    % Visual analysis
    % Analysis using CoM calculated from sway rod data
    [FD_VS,TD_VS] = FRFanalysis_v1(VS',COM_angle',startidx,ppc,fmax,samprate);
    [~]=FRFplotResults_v1(FD_VS,TD_VS,ppc,samprate,'VS ','CoM',ttl1,ttl2);
    StimRemnant_VS=sqrt(FD_VS.f(1)*sum(FD_VS.yi_var));
    SwayRemnant_VS=sqrt(FD_VS.f(1)*sum(FD_VS.yo_var));
    StimRMS_VS=rms(TD_VS.avg_Stim-mean(TD_VS.avg_Stim));
    SwayRMS_VS=rms(TD_VS.avg_Resp-mean(TD_VS.avg_Resp));
    MeanCoh_VS=mean(FD_VS.Cohds(1:Maxf));
    % Fit0 is for PID fit
    ttl3=[ttl2,', VS analysis'];
    [Fit0_VS,mse0_VS] = FRF_Fit0_and_plot_v1(FD_VS.FRFds(1:Maxf),FD_VS.fds(1:Maxf),...
        FD_VS.Cohds(1:Maxf),FD_VS.r(1:Maxf),TD_VS.avg_Stim,TD_VS.avg_Resp...
        ,TD_VS.cl95,ppc,ttl1,ttl3,J,Mass,COM,mgh,SSstim,VSstim,...
        StimRMS_VS,SwayRMS_VS,StimRemnant_VS,SwayRemnant_VS,MeanCoh_VS,samprate);
    % Fit1 is for PD + Torque Feedback fit
    [Fit1_VS,mse1_VS] = FRF_Fit1_and_plot_v1(FD_VS.FRFds(1:Maxf),FD_VS.fds(1:Maxf),...
        FD_VS.Cohds(1:Maxf),FD_VS.r(1:Maxf),TD_VS.avg_Stim,TD_VS.avg_Resp...
        ,TD_VS.cl95,ppc,ttl1,ttl3,J,Mass,COM,mgh,SSstim,VSstim,...
        StimRMS_VS,SwayRMS_VS,StimRemnant_VS,SwayRemnant_VS,MeanCoh_VS,samprate);
    % Analysis using CoM calculated from 0.047 Hz lowpass filtered CoP data
    ttl3=[ttl2,', LP CoM, VS analysis'];
    [FD_VS_LP,TD_VS_LP] = FRFanalysis_v1(VS',COM_LPangle',startidx,ppc,fmax,samprate);
    [~]=FRFplotResults_v1(FD_VS_LP,TD_VS_LP,ppc,samprate,'VS ','CoM_LP',ttl1,ttl3);
    StimRemnant_LP_VS=sqrt(FD_VS_LP.f(1)*sum(FD_VS_LP.yi_var));
    SwayRemnant_LP_VS=sqrt(FD_VS_LP.f(1)*sum(FD_VS_LP.yo_var));
    StimRMS_LP_VS=rms(TD_VS_LP.avg_Stim-mean(TD_VS_LP.avg_Stim));
    SwayRMS_LP_VS=rms(TD_VS_LP.avg_Resp-mean(TD_VS_LP.avg_Resp));
    MeanCoh_LP_VS=mean(FD_VS_LP.Cohds(1:Maxf));
    % Fit1_LP is for PD + Torque Feedback fit using lowpass filtered CoP
    [Fit1_LP_VS,mse1_LP_VS] =
FRF_Fit1_and_plot_v1(FD_VS_LP.FRFds(1:Maxf),FD_VS_LP.fds(1:Maxf),...
        FD_VS_LP.Cohds(1:Maxf),FD_VS_LP.r(1:Maxf),TD_VS_LP.avg_Stim,TD_VS_LP.avg_Resp...
        ,TD_VS_LP.cl95,ppc,ttl1,ttl3,J,Mass,COM,mgh,SSstim,VSstim,...

StimRMS_LP_VS,SwayRMS_LP_VS,StimRemnant_LP_VS,SwayRemnant_LP_VS,MeanCoh_LP_VS,samprate);

    % Average Fit0 results from SS and VS analyses for single final result to save in XL
file
    Fit0.gn=(Fit0_SS.gn+Fit0_VS.gn)/2;
    Fit0.kp=(Fit0_SS.kp+Fit0_VS.kp)/2;
    Fit0.kd=(Fit0_SS.kd+Fit0_VS.kd)/2;
    Fit0.ki=(Fit0_SS.ki+Fit0_VS.ki)/2;
    Fit0.td=(Fit0_SS.td+Fit0_VS.td)/2;
    mse0=(mse0_SS+mse0_VS)/2;
    % Average Fit1 results from SS and VS analyses for single final result to save in XL
file
    Fit1.gn=(Fit1_SS.gn+Fit1_VS.gn)/2;
    Fit1.kp=(Fit1_SS.kp+Fit1_VS.kp)/2;
    Fit1.kd=(Fit1_SS.kd+Fit1_VS.kd)/2;
    Fit1.kt=(Fit1_SS.kt+Fit1_VS.kt)/2;
    Fit1.td=(Fit1_SS.td+Fit1_VS.td)/2;
    mse1=(mse1_SS+mse1_VS)/2;
    MeanCoh=(MeanCoh_SS+MeanCoh_VS)/2; % Coherence, RMS, Remnant values are the same for
Fit0 and Fit1
    StimRMS=(StimRMS_SS+StimRMS_VS)/2;
    SwayRMS=(SwayRMS_SS+SwayRMS_VS)/2;
```

```
    StimRemnant=(StimRemnant_SS+StimRemnant_VS)/2;
    SwayRemnant=(SwayRemnant_SS+SwayRemnant_VS)/2;
    % Average Fit1_LP results from SS and VS analyses for single final result to save in
XL file
    Fit1_LP.gn=(Fit1_LP_SS.gn+Fit1_LP_VS.gn)/2;
    Fit1_LP.kp=(Fit1_LP_SS.kp+Fit1_LP_VS.kp)/2;
    Fit1_LP.kd=(Fit1_LP_SS.kd+Fit1_LP_VS.kd)/2;
    Fit1_LP.kt=(Fit1_LP_SS.kt+Fit1_LP_VS.kt)/2;
    Fit1_LP.td=(Fit1_LP_SS.td+Fit1_LP_VS.td)/2;
    mse1_LP=(mse1_LP_SS+mse1_LP_VS)/2;
    MeanCoh_LP=(MeanCoh_LP_SS+MeanCoh_LP_VS)/2;
    StimRMS_LP=(StimRMS_LP_SS+StimRMS_LP_VS)/2;
    SwayRMS_LP=(SwayRMS_LP_SS+SwayRMS_LP_VS)/2;
    StimRemnant_LP=(StimRemnant_LP_SS+StimRemnant_LP_VS)/2;
    SwayRemnant_LP=(SwayRemnant_LP_SS+SwayRemnant_LP_VS)/2;
end
% print -dwinc
%
% Save analyzed data to mat file
if (SSstim==1)&&(VSstim==0)
    wname=[SubjID,'_A',num2str(ConditionNum),'.mat'];
    save([pathname,wname],'FD_SS','TD_SS','Fit0','mse0','Fit1','mse1',...
        'Fit1_LP','mse1_LP','StimRMS','StimRemnant','SwayRMS','SwayRemnant',...
        'MeanCoh','Maxf','J','Mass','COM','mgh');
end
if (VSstim==1)&&(SSstim==0)
    wname=[SubjID,'_A',num2str(ConditionNum),'.mat'];
    save([pathname,wname],'FD_VS','TD_VS','Fit0','mse0','Fit1','mse1',...
        'Fit1_LP','mse1_LP','StimRMS','StimRemnant','SwayRMS','SwayRemnant',...
        'MeanCoh','Maxf','J','Mass','COM','mgh');
end
if (SSstim==1)&&(VSstim==1)
    wname=[SubjID,'_A',num2str(ConditionNum),'.mat'];
    save([pathname,wname],'FD_SS','TD_SS','Fit0_SS','mse0_SS','Fit1_SS','mse1_SS',...

'Fit1_LP','mse1_LP','StimRMS_SS','StimRemnant_SS','SwayRMS_SS','SwayRemnant_SS',...
        'FD_VS','TD_VS','Fit0_VS','mse0_VS','Fit1_VS','mse1_VS','Fit1_LP','mse1_LP',...
        'MeanCoh','StimRMS_VS','StimRemnant_VS','SwayRMS_VS','SwayRemnant_VS',...

'MeanCoh_LP','StimRMS_LP_VS','StimRemnant_LP_VS','SwayRMS_LP_VS','SwayRemnant_LP_VS',...
        'Maxf','J','Mass','COM','mgh');
end
%%
% Write Fit1 results from sway rod data to Excel file
%   - This only works on a PC since Mac does not support opening Excel.
idx=ConditionNum+1;
[fileID,errormsg]=fopen([pathname,SubjID,'.xlsx']);
if strcmp(errormsg,'No such file or directory') % XL file not yet created
    ColLabels={SubjID;...
        'W';...
        'Kp';...
        'Kd';...
        'Kt';...
        'Td';...
        'FitMSE';...
        'MeanCoh';...
        'StimRMS';...
        'StimRem';...
        'RespRMS';...
        'RespRem';...
        'J';...
        'Mass';...
        'Hcom';...
        'mgh'};
    xlswrite([pathname,SubjID,'.xlsx'],ColLabels);
    [ndata, text, alldata]=xlsread([pathname,SubjID,'.xlsx']);
    alldata{1,idx}=Test;
```

```
    alldata{2,idx}=Fit1.gn;
    alldata{3,idx}=Fit1.kp;
    alldata{4,idx}=Fit1.kd;
    alldata{5,idx}=Fit1.kt;
    alldata{6,idx}=Fit1.td;
    alldata{7,idx}=mse1;
    alldata{8,idx}=MeanCoh;
    alldata{9,idx}=StimRMS;
    alldata{10,idx}=StimRemnant;
    alldata{11,idx}=SwayRMS;
    alldata{12,idx}=SwayRemnant;
    alldata{13,2}=J;    % Body (not including feet) moment of inertia about ankle joint
(kg*m^2)
    alldata{14,2}=Mass; % Total body mass (kg)
    alldata{15,2}=COM;  % CoM height above ankle joint (m)
    alldata{16,2}=mgh;
    xlswrite([pathname,SubjID,'.xlsx'], alldata);
else    % Write into existing XL file
    fclose(fileID);
    [ndata, text, alldata]=xlsread([pathname,SubjID,'.xlsx']);
    alldata{1,idx}=Test;
    alldata{2,idx}=Fit1.gn;
    alldata{3,idx}=Fit1.kp;
    alldata{4,idx}=Fit1.kd;
    alldata{5,idx}=Fit1.kt;
    alldata{6,idx}=Fit1.td;
    alldata{7,idx}=mse1;
    alldata{8,idx}=MeanCoh;
    alldata{9,idx}=StimRMS;
    alldata{10,idx}=StimRemnant;
    alldata{11,idx}=SwayRMS;
    alldata{12,idx}=SwayRemnant;
    xlswrite([pathname,SubjID,'.xlsx'], alldata);
end
%%
%***************************************************************************
%
% Support functions for analysis of a pseudorandom test trial
%
%***************************************************************************
function [Source_File,ETHeader] = ImportEquiTestHeader(filename, startRow, endRow)
%
%   [SOURCE_FILE,NCHeader] =
%   ImportEquiTestHeader(FILENAME, STARTROW, ENDROW) Reads data from rows STARTROW
%   through ENDROW of text file FILENAME.
%
% Example:
%   [Source_File,NCHeader] =
%   ImportEquiTestHeader('FDf3e50178_Word_CR.txt',1, 30);
%
% Auto-generated by MATLAB on 2016/03/28 15:44:54
%
% Initialize variables.
delimiter = ' ';
if nargin<=2
    startRow = 1;
    endRow = 30;
end
% Format string for each line of text:
%   column1: text (%s)
%   column2: text (%s)
% For more information, see the TEXTSCAN documentation.
formatSpec = '%s%s%*s%*s%*s%*s%*s%*s%*s%*s%*s%*s%*s%*s%*s%*s%*s%*s%*s%*s%*s%[^\n\r]';
% Open the text file.
filename
fileID = fopen(filename,'r');
```

```
% Read columns of data according to format string.
% This call is based on the structure of the file used to generate this
% code. If an error occurs for a different file, try regenerating the code
% from the Import Tool.
textscan(fileID, '%[^\n\r]', startRow(1)-1, 'ReturnOnError', false);
dataArray = textscan(fileID, formatSpec, endRow(1)-startRow(1)+1, 'Delimiter', delimiter,
'MultipleDelimsAsOne', true, 'ReturnOnError', false);
for block=2:length(startRow)
    frewind(fileID);
    textscan(fileID, '%[^\n\r]', startRow(block)-1, 'ReturnOnError', false);
    dataArrayBlock = textscan(fileID, formatSpec, endRow(block)-startRow(block)+1,
'Delimiter', delimiter, 'MultipleDelimsAsOne', true, 'ReturnOnError', false);
    for col=1:length(dataArray)
        dataArray{col} = [dataArray{col};dataArrayBlock{col}];
    end
end
% Close the text file.
fclose(fileID);
% Allocate imported array to column variable names
Source_File = dataArray{:, 1};
ETHeader = dataArray{:, 2};
end

function ETData = ImportEquiTestData_SingleStim(filename, startRow, endRow)
%
%   ETData = ImportEquiTestData_SingleStim(FILENAME, STARTROW, ENDROW) Reads data
%   from rows STARTROW through ENDROW of text file FILENAME.
%
% Example:
%   ETData = ImportEquiTestData_SingleStim('FDf3e50178MacSave.txt', 33, 24632);
%
% Auto-generated by MATLAB on 2016/03/28 14:17:06

% Initialize variables.
delimiter = ' ';
if nargin<=2
    startRow = 33;
    endRow = inf;
end
% For more information, see the TEXTSCAN documentation.
formatSpec = '%f%f%f%f%f%f%f%f%f%f%f%f%f%f%f%f%f%f%f%f%f%f%f%f%f%[^\n\r]';
% Open the text file.
fileID = fopen(filename,'r');
% Read columns of data according to format string.
% This call is based on the structure of the file used to generate this
% code. If an error occurs for a different file, try regenerating the code
% from the Import Tool.
textscan(fileID, '%[^\n\r]', startRow(1)-1, 'ReturnOnError', false);
dataArray = textscan(fileID, formatSpec, endRow(1)-startRow(1)+1, 'Delimiter', delimiter,
'MultipleDelimsAsOne', true, 'EmptyValue' ,NaN,'ReturnOnError', false);
for block=2:length(startRow)
    frewind(fileID);
    textscan(fileID, '%[^\n\r]', startRow(block)-1, 'ReturnOnError', false);
    dataArrayBlock = textscan(fileID, formatSpec, endRow(block)-startRow(block)+1,
'Delimiter', delimiter, 'MultipleDelimsAsOne', true, 'EmptyValue' ,NaN,'ReturnOnError',
false);
    for col=1:length(dataArray)
        dataArray{col} = [dataArray{col};dataArrayBlock{col}];
    end
end
% Close the text file.
fclose(fileID);
% Create output variable
ETData = [dataArray{1:end-1}];
end

function ETData = ImportEquiTestData_DualStim(filename, startRow, endRow)
```

```
%
%   ETData = ImportEquiTestData_DualStim(FILENAME, STARTROW, ENDROW) Reads data
%   from rows STARTROW through ENDROW of text file FILENAME.
%
% Example:
%   ETData = ImportEquiTestData_DualStim('FDf3e50178MacSave.txt', 33, 24632);
%
% Auto-generated by MATLAB on 2016/03/28 14:17:06

% Initialize variables.
delimiter = ' ';
if nargin<=2
    startRow = 33;
    endRow = inf;
end
formatSpec = '%f%f%f%f%f%f%f%f%f%f%f%f%f%f%f%f%f%f%f%f%f%f%f%f%f%[^\n\r]';
% Open the text file.
fileID = fopen(filename,'r');
% Read columns of data according to format string.
% This call is based on the structure of the file used to generate this
% code. If an error occurs for a different file, try regenerating the code
% from the Import Tool.
textscan(fileID, '%[^\n\r]', startRow(1)-1, 'ReturnOnError', false);
dataArray = textscan(fileID, formatSpec, endRow(1)-startRow(1)+1, 'Delimiter', delimiter,
'MultipleDelimsAsOne', true, 'EmptyValue' ,NaN,'ReturnOnError', false);
for block=2:length(startRow)
    frewind(fileID);
    textscan(fileID, '%[^\n\r]', startRow(block)-1, 'ReturnOnError', false);
    dataArrayBlock = textscan(fileID, formatSpec, endRow(block)-startRow(block)+1,
'Delimiter', delimiter, 'MultipleDelimsAsOne', true, 'EmptyValue' ,NaN,'ReturnOnError',
false);
    for col=1:length(dataArray)
        dataArray{col} = [dataArray{col};dataArrayBlock{col}];
    end
end
% Close the text file.
fclose(fileID);
% Create output variable
ETData = [dataArray{1:end-1}];
end

function [FD,TD] = FRFanalysis_v1(SigIN,SigOUT,startidx,ppc,fmax,samprate)
%
% [FD,TD] = FRFanalysis_v1(SigIN,SigOUT,startidx,ppc,fmax,samprate)    19-Apr-2013
%   Inputs:
%       SigIN:  Input time series
%       SigOUT:  Output time series
%       startidx:   Start index of first stimulus cycle
%       ppc:        Points per stimulus cycle
%       fmax:       Maximum # of frequency points for dft calculation
%       samprate:   Sampling rate of time series
%   Outputs: FD = Frequency domain results, TD = time domain results
%       FD.fd:          FRF frequencies (decimated for PRTS components but not smoothed)
%       FD.FRFd:        FRF values (complex, decimated for PRTS components but not
smoothed)
%       FD.FRFd_var:    FRF variance (complex, decimated for PRTS components but not
smoothed)
%       FD.Cohd:        Coherence values (decimated for PRTS components but not smoothed)
%       FD.fds:         FRF frequencies (decimated for PRTS components and smoothed across
freqency)
%       FD.FRFds:       FRF values (complex, decimated for PRTS components and smoothed
across freqency)
%       FD.Cohds:       Coherence values (decimated for PRTS components and smoothed across
freqency)
%       FD.dof:         degrees of freedom of smoothed FRF
%       FD.Fdist:       F distribution statistic for smoothe FRF
```

```
%       FD.r:          error radius on smoothed FRF
%       FD.yiid_mean:  mean input power spectrum at PRTS component freqs - scaled for
df*sum=ms
%       FD.yood_mean:  mean output power spectrum at PRTS component freqs - scaled for
df*sum=ms
%       FD.yoid_mean:  mean cross power spectrum at PRTS component freqs - scaled for
df*sum=ms
%       FD.f:          FRF frequencies (not decimated for PRTS components)
%       FD.yii_mean:   mean input power spectrum at all freqs - scaled for df*sum=ms
%       FD.yoo_mean:   mean output power spectrum at all freqs - scaled for df*sum=ms
%       FD.yoi_mean:   mean cross power spectrum at all freqs - scaled for df*sum=ms
%       FD.yi_var:     variance of input stimulus spectrum - scaled for df*sum=ms
%       FD.yo_var:     variance of output stimulus spectrum - scaled for df*sum=ms
%
%       TD.avg_Stim:      % average SigIN
%       TD.avg_Resp:      % average SigOUT
%       TD.cl95=cl95;     % 95% conficence limits on mean SigOUT

% Frequency domain variables for accumulating results
f=(samprate/ppc)*(1:fmax);   % frequency vector for dft

yi=zeros(1,fmax);       % SigIN dft  - fmax freqs
yo=zeros(1,fmax);       % SigOUT dft - fmax freqs

yoi=zeros(1,fmax);      % used for coherence calculations
yii=zeros(1,fmax);
yoo=zeros(1,fmax);

%
% Time domain variables for accumulating results
avg_SigIN=zeros(1,ppc);
avg_SigOUT=zeros(1,ppc);    % average COM position
RESP_SigOUT=[];             % array to accumlate cycles of response position
resp_offset=[];         % mean angle of subject COM
RMS_cyc=[];             % rms values of CoM angle for each cycle
RMS_SigOUT=[];          % rms value of CoM angle of average cycle
RMS_SigIN=[];           % rms value of average stimulus cycle
PP_cyc=[];              % peak-to-peak of COM angle for each cycle
PP_SigOUT=[];               % peak-to-peak of COM angle of average cycle
PP_SigIN=[];                % peak-to-peak of stimulus average cycle

psfactor=1/(2*samprate*ppc);    % Factor for scaling power spectra such that integration
across
% all frequencies (deltaf*sum(power spectral values) gives the
% mean square value. The power spectra are calculated beginning
% with 2*fft (2*fft gives the one sided spectrum).

cycles=fix((length(SigIN)-startidx)/(ppc)); % # PRTS cycles
cyc=0;
for k=2:cycles          % skip 1st cycle
    cyc=cyc+1;
    yi_raw=fft(SigIN((startidx+(k-1)*ppc):(startidx+k*ppc-1))');        % SigIN dft
    yi_raw=conj(yi_raw);
    yo_raw=fft(SigOUT((startidx+(k-1)*ppc):(startidx+k*ppc-1))');       % SigOUT dft
    yo_raw=conj(yo_raw);

    yi_raw2=2*yi_raw(2:(fmax+1))';       % ignore DC values and multiply by 2 for one-
sided dft spectra
    yo_raw2=2*yo_raw(2:(fmax+1))';

    yi(cyc,:)=yi_raw2;                 % accumulate dft spectra - fmax points
    yo(cyc,:)=yo_raw2;

    yoi(cyc,:)=psfactor*yo_raw2.*conj(yi_raw2);     % accumulate scaled power spectra and
cross power spectra
    yii(cyc,:)=psfactor*abs(yi_raw2).*abs(yi_raw2);
    yoo(cyc,:)=psfactor*abs(yo_raw2).*abs(yo_raw2);
```

```
    % calc smoothed, decimated power spectral values to be used for FRF variance
calculation
    xois(cyc,:)=EquiTest_smooth_12pt(decimate2(psfactor*yo_raw2.*conj(yi_raw2),2));
    xiis(cyc,:)=EquiTest_smooth_12pt(decimate2(psfactor*abs(yi_raw2).*abs(yi_raw2),2));
    xoos(cyc,:)=EquiTest_smooth_12pt(decimate2(psfactor*abs(yo_raw2).*abs(yo_raw2),2));

    avg_SigIN=avg_SigIN+SigIN((startidx+(k-1)*ppc):(startidx+k*ppc-1));  % SigIN time
averages
    resp=SigOUT((startidx+(k-1)*ppc):(startidx+k*ppc-1));
    avg_SigOUT=avg_SigOUT+resp;                                          % SigOUT time
averages
    RESP_SigOUT(cyc,:)=resp;                               % accumulated response SigOUT
position cycles
    RMS_cyc(cyc)=sqrt(mean((resp-mean(resp)).^2));         % calculate rms value of SigOUT
angle for each cycle
    PP_cyc(cyc)=max(resp)-min(resp);                       % calc peak-to-peak of SigOUT
angle for each cycle
end %for k=2:cycles

avg_SigIN=avg_SigIN/cyc;    % average stimulus
avg_SigOUT=avg_SigOUT/cyc;  % average response
cl95=1.96*(std(RESP_SigOUT))/sqrt(cyc); % 95% confidence limits about mean response pos
resp_offset=mean(avg_SigOUT);                        % mean angle of subject SigOUT
RMS_SigOUT=mean(sqrt((avg_SigOUT-mean(avg_SigOUT)).^2)); % rms of average response (deg)
RMS_SigIN=mean(sqrt((avg_SigIN-mean(avg_SigIN)).^2)); % rms of average stimulus (deg)
PP_SigOUT=max(avg_SigOUT)-min(avg_SigOUT);  % peak-to-peak value of average response (deg)
PP_SigIN=max(avg_SigIN)-min(avg_SigIN); % peak-to-peak value of average stimulus (deg)

yi_mean=mean(yi,1);
yo_mean=mean(yo,1);

yoi_mean=mean(yoi,1);
yii_mean=mean(yii,1);
yoo_mean=mean(yoo,1);

yi_var=zeros(1,fmax);  % for calculation of variance of spectra
yo_var=zeros(1,fmax);
yoi_var=zeros(1,fmax);
for k=1:cyc
    yi_var=yi_var+abs(yi(cyc,:)-yi_mean).^2;
    yo_var=yo_var+abs(yo(cyc,:)-yo_mean).^2;
    yoi_var=yoi_var+(yo(cyc,:)-yo_mean).*conj(yi(cyc,:)-yi_mean);
end
yi_var=psfactor*yi_var/(cyc-1);
yo_var=psfactor*yo_var/(cyc-1);
yoi_var=psfactor*yoi_var/(cyc-1);

% Calculate variance of FRF - Pintelon & Schoukens, System Identification: A Frequency
Domain Approach,
%   Second Edition, John Wiley & Sons, 2012, eq 2-38
%   - calculate at all frequencies, then decimate to get final values at PRTS frequencies
with stimulus energy
%
FRF=yo_mean./yi_mean;   % FRF from position data - Pintelon & Schoukens eq 2-36

FRF_var=(abs(FRF).^2).*(yo_var./(psfactor*abs(yo_mean).^2)+yi_var./(psfactor*abs(yi_mean).
^2)+2*real(yoi_var./(psfactor*yo_mean.*conj(yi_mean))));
FRFd_var=decimate2(FRF_var,2);
FRFd=decimate2(FRF,2);

fd=decimate2(f,2);      % frequencies with SigIN PRTS energy
yid_mean=decimate2(yi_mean,2);
yod_mean=decimate2(yo_mean,2);

yoid_mean=decimate2(yoi_mean,2);
```

```
yiid_mean=decimate2(yii_mean,2);
yood_mean=decimate2(yoo_mean,2);

Cohd=(abs(yoid_mean).^2)./(yiid_mean.*yood_mean);

fdd=decimate2(f(2:fmax),2);     % frequencies without SigIN PRTS energy
yipdd=decimate2(yi_mean(2:fmax),2);
yopdd=decimate2(yo_mean(2:fmax),2);

fds=EquiTest_smooth_12pt(fd);
FRFds=EquiTest_smooth_12pt(FRFd);
yoids_mean=EquiTest_smooth_12pt(yoid_mean);
yiids_mean=EquiTest_smooth_12pt(yiid_mean);
yoods_mean=EquiTest_smooth_12pt(yood_mean);

Cohds=(abs(yoids_mean).^2)./(yiids_mean.*yoods_mean); % Coherence from smoothed power
spectra

[dof,Fdist]=EquiTest_Degrees_of_Freedom(cyc);

r=sqrt((((2*(1-Cohds)).*Fdist)./(dof-2)).*(yoods_mean./yiids_mean));

FD.fd=fd;                % FRF frequencies (decimated for PRTS components but not smoothed)
FD.FRFd=FRFd;            % FRF values (complex, decimated for PRTS components but not
smoothed)
FD.FRFd_var=FRFd_var;    % FRF variance (complex, decimated for PRTS components but not
smoothed)
FD.Cohd=Cohd;            % Coherence values (decimated for PRTS components but not
smoothed)
FD.fds=fds;              % FRF frequencies (decimated for PRTS components and smoothed
across freqency)
FD.FRFds=FRFds;          % FRF values (complex, decimated for PRTS components and smoothed
across freqency)
FD.Cohds=Cohds;          % Coherence values (decimated for PRTS components and smoothed
across freqency)
FD.dof=dof;              % degrees of freedom of smoothed FRF
FD.Fdist=Fdist;          % F distribution statistic for smoothed FRF
FD.r=r;                  % error radius on smoothed FRF
FD.yiid_mean=yiid_mean; % mean input power spectrum at PRTS component freqs - scaled for
df*sum=ms
FD.yood_mean=yood_mean; % mean output power spectrum at PRTS component freqs - scaled for
df*sum=ms
FD.yoid_mean=yoid_mean; % mean cross power spectrum at PRTS component freqs - scaled for
df*sum=ms
FD.f=f;                  % all FRF frequencies (not decimated for PRTS components)
FD.yii_mean=yii_mean;    % mean input power spectrum at all freqs - scaled for df*sum=ms
FD.yoo_mean=yoo_mean;    % mean output power spectrum at all freqs - scaled for df*sum=ms
FD.yoi_mean=yoi_mean;    % mean cross power spectrum at all freqs - scaled for df*sum=ms
FD.yi_var=yi_var;        % variance of input power spectrum at all freqs - scaled for
df*sum=ms
FD.yo_var=yo_var;        % variance of output power spectrum at all freqs - scaled for
df*sum=ms

TD.avg_Stim=avg_SigIN;
TD.avg_Resp=avg_SigOUT;
TD.cl95=cl95;                % 95% conficence limits on mean SigOUT

end

function [x] = EquiTest_smooth_12pt(y)
%EquiTest_smooth_12pt function - Spectrum smoothing by averaging adjacent frequency points
with
% an increasing number of points averaged with increasing frequency. Input is
% a vector of 50 values (not all are used). Return is a row vector with 12 values.
%
x=[y(1) mean(y(1:2)) y(2) mean(y(2:3)) mean(y(3:4)) mean(y(4:5)) mean(y(5:7)) mean(y(6:9))
mean(y(8:11)) mean(y(10:13)),...
```

```
    mean(y(12:16)) mean(y(16:20))]; % mean(y(20:25)) mean(y(25:32)) mean(y(32:40)
mean(y(40:50))];
end

function [dof,Fdist]=EquiTest_Degrees_of_Freedom(cyc)
%
%   Input:
%       cyc: number of data cycles averaged to produce FRF (must be 2 or
%       greater)
%   Output:
%       dof = degrees of freedom for smoothed FRF data
%       Fdist = F distribution values for p=0.95 (alpha=0.05)
%
% dof = 2 * number of complex Fourier components averaged (p 219 in Otnes &
% Enochson, Digital Time Series Analysis, John Wiley & Sons, 1972)
FRFpts_averaged=[1,2,1,2,2,2,3,4,4,4,5,5]; %,6,8,9,11]; % 12 (16) frequency points
dof_per_cycle=2*FRFpts_averaged;
dof=cyc*dof_per_cycle;
%
% Fdist value for use in setting confidence limits on FRFs is F(n1,n2;alpha)
%   with n1 = 2, n2 = dof-2, alpha = 1-p (Otnes & Enochson, page 357).
%
% If Matlab Statistics toolbox is available then Fdist is given by:
%
%       Fdist=finv(0.95,2*ones(size(dof_per_cycle)),cyc*dof_per_cycle-2);
%
% Otherwise (for the specific set of averaged FRF pts defined above:
switch cyc
    case 2
        Fdist=[19.0000 5.1433 19.0000 5.1433 5.1433 5.1433 4.1028 3.7389 3.7389 3.7389
3.5546 3.5546];
    case 3
        Fdist=[6.9443 4.1028 6.9443 4.1028 4.1028 4.1028 3.6337 3.4434 3.4434 3.4434
3.3404 3.3404];
    case 4
        Fdist=[5.1433 3.7389 5.1433 3.7389 3.7389 3.7389 3.4434 3.3158 3.3158 3.3158
3.2448 3.2448];
    case 5
        Fdist=[4.4590 3.5546 4.4590 3.5546 3.5546 3.5546 3.3404 3.2448 3.2448 3.2448
3.1907 3.1907];
    case 6
        Fdist=[4.1028 3.4434 4.1028 3.4434 3.4434 3.4434 3.2759 3.1996 3.1996 3.1996
3.1559 3.1559];
    case 7
        Fdist=[4.1028 3.4434 4.1028 3.4434 3.4434 3.4434 3.2759 3.1996 3.1996 3.1996
3.1559 3.1559];
    case 8
        Fdist=[3.7389 3.3158 3.7389 3.3158 3.3158 3.3158 3.1996 3.1453 3.1453 3.1453
3.1138 3.1138];
    case 9
        Fdist=[3.6337 3.2759 3.6337 3.2759 3.2759 3.2759 3.1751 3.1277 3.1277 3.1277
3.1001 3.1001];
    case 10
        Fdist=[3.5546 3.2448 3.5546 3.2448 3.2448 3.2448 3.1559 3.1138 3.1138 3.1138
3.0892 3.0892];
    case 11
        Fdist=[3.4928 3.2199 3.4928 3.2199 3.2199 3.2199 3.1404 3.1026 3.1026 3.1026
3.0804 3.0804];
    case 12
        Fdist=[3.4434 3.1996 3.4434 3.1996 3.1996 3.1996 3.1277 3.0933 3.0933 3.0933
3.0731 3.0731];
end
end

function [out] = FRFplotResults_v1(FD,TD,CyclePts,samprate,StimLabel,RespLabel,ttl1,ttl2)
%
```

```
% Plot results from FRF analysis
%
f=FD.fds;
FRF=FD.FRFds;
Coherence=FD.Cohds;
ErrorRadius=FD.r;
AvgStim=TD.avg_Stim;
AvgResp=TD.avg_Resp;
ConfLim95=TD.cl95;
fps=FD.fd;
yii=FD.yiid_mean;
yoo=FD.yood_mean;

GainLB=0.01;
GainUB=10;
if max(abs(FRF))>9
    GainLB=0.1;
    GainUB=100;
end

figure; SetPrintFullSize;
subplot(321);loglog(f,abs(FRF),'mo-','markersize',4)
axis([.01 4 GainLB GainUB]);
hold on
error_bars_log(f,abs(FRF),ErrorRadius,0.05);
hold off
title(ttl1,'Interpreter','none');
ylabel([RespLabel,'/',StimLabel,' magitude'],'Interpreter','none')

pp=180/pi*asin((ErrorRadius)./abs(FRF)); % calculate error bars on phase
subplot(323);semilogx(f,unwrap(angle(FRF))*180/pi,'mo-','markersize',4)
axis([.01 4 -400 100]);
hold on
error_bars_log(f,unwrap(angle(FRF))*180/pi,pp,0.05);
hold off
ylabel([RespLabel,'/',StimLabel,' phase'],'Interpreter','none')

subplot(325);semilogx(f,Coherence,'mo-','markersize',4);
axis([0.01 4 0 1]);
ylabel([RespLabel,'/',StimLabel,' Coherence'],'Interpreter','none')
xlabel('Freq (Hz)')

t=(0:(CyclePts-1))/samprate;
subplot(322);plot(t,AvgStim);
axis([0 CyclePts/samprate -4 4]);
title(ttl2)
ylabel([StimLabel 'Stim Average (deg)'])

AvgResp=AvgResp-mean(AvgResp);
subplot(324);plot(t,AvgResp+ConfLim95,'g',t,AvgResp-ConfLim95,'g',t,AvgResp,'b');
axis([0 CyclePts/samprate -4 4]);
ylabel([RespLabel ' Resp Average+/-95% c.l. (deg)'],'Interpreter','none')
xlabel('Time (s)')
subplot(326); loglog(fps,yii,'.b',fps,yoo,'.r');
axis([0.01 10 0.0001 1000]);
xlabel('Freq (Hz)')
ylabel('units^2/Hz')
title('Power Spectra stim (b), resp (r)')

out=0;
end

function [Fit0,mse0] =
FRF_Fit0_and_plot_v1(FRF,F,COH,ERROR,AvgStim,AvgResp,ConfLim95,pts,ttl1,ttl2,J,m,Hcom,mgh,
SSstim,VSstim,...
    StimRMS,SwayRMS,StimRemnant,SwayRemnant,MeanCoh,samprate)
%FRF_Fit0_and_plot_v1
```

```
% Function to perform optimization fit of balance control model with PID
% neural controller and no passive ankle torque.
%
Nfit=5; % Nfit separate fits performed with different initial parameter values
[Fit0,mse0]=FRF_Fit0_v1(F,FRF,COH,J,mgh,Nfit);

gn=Fit0.gn; % sensory weight
kp=Fit0.kp; % neural controller stiffness factor
kd=Fit0.kd; % neural controller damping factor
ki=Fit0.ki; % neural controller integration factor
td=Fit0.td; % time delay
[gn,kp,kd,ki,td,mse0]
%
% Calculate gain and phase curves of fit for display
%
f=logspace(-2,1,300);
f=f(50:250);
w=2*pi*f;
s=sqrt(-1)*w;
B=(ones(size(w)))./(J*(s.*s)-mgh*ones(size(w)));
NC=kd*s+kp+(ki*ones(size(s)))./s;
TD=(cos(w*td)-sqrt(-1)*sin(w*td));
N=NC.*TD;

tf=(gn*B.*N)./(ones(size(w)) + B.*N);

gfit1=abs(tf);              % fit gain and phase for display
pfit1=180/pi*unwrap(angle(tf));

figure
SetPrintFullSize;

subplot(321);loglog(F,abs(FRF),'bo',f,gfit1,'r','markersize',4)
axis([.01 6 .01 10])
title(ttl1,'Interpreter','none');
ylabel('CoM/Stim magitude')
hold on
error_bars_log(F,abs(FRF),ERROR,0.05);
v1=['J  ',num2str(J),' kg-m^2'];
v2=['m  ',num2str(m),' kg'];
v3=['h  ',num2str(Hcom),'m'];
v4=['mgh  ',num2str(mgh)];
text(.02,0.2,v1)
text(.02,0.1,v2)
text(.02,0.050,v3)
text(.02,0.025,v4)
hold off
PhaseError=180/pi*asin((ERROR)./abs(FRF)); % calculate error bars on phase
subplot(323);semilogx(F,180/pi*unwrap(angle(FRF)),'bo',f,pfit1,'r','markersize',4)
axis([.01 6 -400 100])
ylabel('CoM/Stim phase')
hold on
error_bars_log(F,180/pi*unwrap(angle(FRF)),PhaseError,0.05);
if (SSstim==1)&&(VSstim==0)
    v1=['Fit: Wp  ',num2str(gn)];      % Fit 1 parameters
end
if (SSstim==0)&&(VSstim==1)
    v1=['Fit: Wv  ',num2str(gn)];
end
if (SSstim==1)&&(VSstim==1)
    v1=['Fit: Wp+Wv  ',num2str(gn)];
end
v2=['     Kp  ',num2str(kp),' Nm/rad'];
v3=['     Kd  ',num2str(kd),' Nms/rad'];
v4=['     Ki  ',num2str(ki),' Nm/(rad s)'];
v5=['     Td  ',num2str(td),' s'];
```

```
v6=['     mse ',num2str(mse0)];
text(.01,-100,v1)
text(.01,-150,v2)
text(.01,-200,v3)
text(.01,-250,v4)
text(.01,-300,v5)
text(.01,-350,v6)
hold off
subplot(325);semilogx(F,COH,'bo-','markersize',4);
axis([0.01 6 0 1]);
ylabel('CoM/Stim Coherence')
xlabel('Freq (Hz)')
v1=['     mean Coherence  ',num2str(MeanCoh)];
text(.01,0.15,v1)
tc=(1:length(AvgStim))/samprate;
subplot(322);plot(tc,AvgStim);
axis([0 pts/samprate -4 4])
title(ttl2,'Interpreter','none');
ylabel('Stim Average (deg)')
v1=['     Stim RMS  ',num2str(StimRMS)];
v2=['     Stim Remnant  ',num2str(StimRemnant)];
text(1,-2,v1)
text(1,-2.5,v2)
subplot(324);plot(tc,AvgResp+ConfLim95-mean(AvgResp),'g',tc,AvgResp-ConfLim95-
mean(AvgResp),'g',tc,AvgResp-mean(AvgResp),'b');
axis([0 pts/samprate -4 4])
ylabel('Resp Average+/-95% c.l. (deg)')
xlabel('Time (s)')
v1=['     Resp RMS  ',num2str(SwayRMS)];
v2=['     Resp Remnant  ',num2str(SwayRemnant)];
text(1,-2,v1)
text(1,-2.5,v2)
end

function [Fit,fval]=FRF_Fit0_v1(F,FRF,COH,J,mgh,Nfit)
%   FRF_Fit0_v1.m
%
% Inputs:
%   F: Vector of frequencies
%   FRF: Experimental FRF values (complex numbers)
%   COH: Coherence
%   J: Moment of inertia about ankle joint axis
%   mgh: mass x gravity x CoM height
%   Nfit: number of fits to perform with different random initial parameter values
%
%   Calls fmincon from Optimization Toolbox
%
%   Outputs:
%       Fit.gn = FRF gain constant
%       Fit.kp = Neural controller active stiffness (units = N m/rad)
%       Fit.kd = Neural controller active damping (units = N m s/rad)
%       Fit.ki = Neural controller active integration (units = N m/(rad s))
%       Fit.td = Time delay (units = s)
%       fval = value of error function for optimal fit
%
ferr=@(P_fit_i)FRFfiterr_Fit0_v1(P_fit_i,F,FRF,COH,J,mgh); % Pasma error

random_lb=[0.1 1.2*mgh 0.3*mgh 0.01*mgh 0.1]; % range of initial parameter values
random_ub=[0.9 1.6*mgh 0.6*mgh 0.5*mgh  0.2];

fit_lb=[0.001 mgh   0.1*mgh 0.01*mgh  0.02];    % fit lower bounds
fit_ub=[1.1   4*mgh 2*mgh   3*mgh     0.35];    % fit upper bounds
A=[];
B=[];
Aeq=[];
Beq=[];
options=optimset('fmincon');
```

```
options=optimset(options,'MaxFunEvals',10000,'TolFun',1e-
10,'MaxIter',1000,'Display','off');

fval=100;   % initialize error value with extremely large value
for ii=1:Nfit
    P_fit_i=random_lb+rand(size(random_lb)).*(random_ub-random_lb); % initial values
    [P_fit_j,fval_j]=fmincon(ferr,P_fit_i,A,B,Aeq,Beq,fit_lb,fit_ub,[],options);
    if fval_j<fval % if current fit has lower fval, save that fit
        P_fit=P_fit_j;
        fval=fval_j;
    end
end

Fit.gn=P_fit(1);
Fit.kp=P_fit(2); % in radian units
Fit.kd=P_fit(3);
Fit.ki=P_fit(4);
Fit.td=P_fit(5);
end

function mse = FRFfiterr_Fit0_v1(P_fit,F,FRF,COH,J,mgh)
% FRFfiterr_Fit0_v1
%   Function to calculate error between experimental FRF data and
%   model FRF on each iteration performed by the fmincon function.
gn=P_fit(1); % sensory weight
kp=P_fit(2); % neural controller stiffness factor
kd=P_fit(3); % neural controller damping factor
ki=P_fit(4); % neural controller integration factor
td=P_fit(5); % time delay
%
%  Form model FRF estimate from current parameters
%
w=2*pi*F;
s=1i*w;
B=(ones(size(w)))./(J*(s.*s)-mgh*ones(size(w)));
NC=kd*s+kp+(ki*ones(size(s)))./s;
TD=(cos(w*td)-1i*sin(w*td));
N=NC.*TD;
frf=(gn*B.*N)./(ones(size(w)) + B.*N);
%
% fit error
%
d2=(FRF-frf)./abs(frf); % normalize individual vectors by magnitude of fit at each
frequency
mse=d2*d2';
end

function [Fit1,mse1] =
FRF_Fit1_and_plot_v1(FRF,F,COH,ERROR,AvgStim,AvgResp,ConfLim95,pts,ttl1,ttl2,J,m,Hcom,mgh,
SSstim,VSstim,...
    StimRMS,SwayRMS,StimRemnant,SwayRemnant,MeanCoh,samprate)
%FRFfit_and_plot_v1
% Function to perform optimization fit of balance control model with PD
% neural controller, no passive ankle torque, and integrated torque feedback.
%
Nfit=5; % Nfit separate fits performed with different initial parameter values
[Fit1,mse1]=FRF_Fit1_v1(F,FRF,COH,J,mgh,Nfit);

gn=Fit1.gn; % sensory weight
kp=Fit1.kp; % neural controller stiffness
kd=Fit1.kd; % neural controller damping
kt=Fit1.kt; % integrated torque feedback gain constant
td=Fit1.td; % time delay
% mse=mse1;
[gn,kp,kd,kt,td,mse1]
%
```

```
% Calculate gain and phase curves of fit for display
%
f=logspace(-2,1,300);
f=f(50:250);
w=2*pi*f;
s=sqrt(-1)*w;
B=(ones(size(w)))./(J*(s.*s)-mgh*ones(size(w)));
NC=kd*s+kp;
FF=(kt*ones(size(w)))./(s);
TD=(cos(w*td)-sqrt(-1)*sin(w*td));
N=NC.*TD;

tf=(gn*B.*N)./(ones(size(w)) - FF.*N + B.*N);

gfit1=abs(tf);              % fit gain and phase for display
pfit1=180/pi*unwrap(angle(tf));

figure
SetPrintFullSize;

subplot(321);loglog(F,abs(FRF),'bo',f,gfit1,'r','markersize',4)
axis([.01 6 .01 10])
title(ttl1,'Interpreter','none');
ylabel('CoM/Stim magitude')
hold on
error_bars_log(F,abs(FRF),ERROR,0.05);
v1=['J  ',num2str(J),' kg-m^2'];
v2=['m  ',num2str(m),' kg'];
v3=['h  ',num2str(Hcom),'m'];
v4=['mgh  ',num2str(mgh)];
text(.02,0.2,v1)
text(.02,0.1,v2)
text(.02,0.050,v3)
text(.02,0.025,v4)
hold off
PhaseError=180/pi*asin((ERROR)./abs(FRF)); % calculate error bars on phase
subplot(323);semilogx(F,180/pi*unwrap(angle(FRF)),'bo',f,pfit1,'r','markersize',4)
axis([.01 6 -400 100])
ylabel('CoM/Stim phase')
hold on
error_bars_log(F,180/pi*unwrap(angle(FRF)),PhaseError,0.05);
if (SSstim==1)&&(VSstim==0)
    v1=['Fit: Wp  ',num2str(gn)];      % Fit 1 parameters
end
if (SSstim==0)&&(VSstim==1)
    v1=['Fit: Wv  ',num2str(gn)];
end
if (SSstim==1)&&(VSstim==1)
    v1=['Fit: Wp+Wv  ',num2str(gn)];
end
v2=['     Kp  ',num2str(kp),' Nm/rad'];
v3=['     Kd  ',num2str(kd),' Nms/rad'];
v4=['     Kt  ',num2str(kt),' rad/Nm'];
v5=['     Td  ',num2str(td),' s'];
v6=['     mse ',num2str(mse1)];
text(.01,-100,v1)
text(.01,-150,v2)
text(.01,-200,v3)
text(.01,-250,v4)
text(.01,-300,v5)
text(.01,-350,v6)
hold off
subplot(325);semilogx(F,COH,'bo-','markersize',4);
axis([0.01 6 0 1]);
ylabel('CoM/Stim Coherence')
xlabel('Freq (Hz)')
v1=['     mean Coherence  ',num2str(MeanCoh)];
```

```
text(.01,0.15,v1)
tc=(1:length(AvgStim))/samprate;
subplot(322);plot(tc,AvgStim);
axis([0 pts/samprate -4 4])
title(ttl2,'Interpreter','none');
ylabel('Stim Average (deg)')
v1=['     Stim RMS   ',num2str(StimRMS)];
v2=['     Stim Remnant   ',num2str(StimRemnant)];
text(1,-2,v1)
text(1,-2.5,v2)
subplot(324);plot(tc,AvgResp+ConfLim95-mean(AvgResp),'g',tc,AvgResp-ConfLim95-
mean(AvgResp),'g',tc,AvgResp-mean(AvgResp),'b');
axis([0 pts/samprate -4 4])
ylabel('Resp Average+/-95% c.l. (deg)')
xlabel('Time (s)')
v1=['     Resp RMS   ',num2str(SwayRMS)];
v2=['     Resp Remnant   ',num2str(SwayRemnant)];
text(1,-2,v1)
text(1,-2.5,v2)
end

function [Fit,fval]=FRF_Fit1_v1(F,FRF,COH,J,mgh,Nfit)
%   FRF_Fit1_v1.m
%
% Inputs:
%   F: Vector of frequencies
%   FRF: Experimental FRF values (complex numbers)
%   COH: Coherence
%   J: Moment of inertia about ankle joint axis
%   mgh: mass x gravity x CoM height
%   Nfit: number of fits to perform with different random initial parameter values
%
%   Calls fmincon from Optimization Toolbox
%
%   Outputs:
%       Fit.gn = FRF gain constant
%       Fit.kp = Neural controller active stiffness (units = N m/rad)
%       Fit.kd = Neural controller active damping (units = N m s/rad)
%       Fit.kt = Torque feedback gain constant of integrator (units = rad/(N m s))
%       Fit.td = Time delay (units = s)
%       fval = value of error function for optimal fit
%
ferr=@(P_fit_i)FRFfiterr_Fit1_v1(P_fit_i,F,FRF,COH,J,mgh); % Pasma error

random_lb=[0.1 1.2*mgh 0.3*mgh 0.00005 0.1]; % range of initial parameter values
random_ub=[0.9 1.6*mgh 0.6*mgh 0.0002  0.2];

fit_lb=[0.001 mgh  0.1*mgh 0.00000001  0.02];   % fit lower bounds
fit_ub=[1.1  4*mgh 2*mgh 4*pi/180   0.35];  % fit upper bounds
A=[];
B=[];
Aeq=[];
Beq=[];
options=optimset('fmincon');
options=optimset(options,'MaxFunEvals',10000,'TolFun',1e-
10,'MaxIter',1000,'Display','off');

fval=100;   % initialize error value with extremely large value
for ii=1:Nfit
    P_fit_i=random_lb+rand(size(random_lb)).*(random_ub-random_lb); % initial values
    [P_fit_j,fval_j]=fmincon(ferr,P_fit_i,A,B,Aeq,Beq,fit_lb,fit_ub,[],options);
    if fval_j<fval % if current fit has lower fval, save that fit
        P_fit=P_fit_j;
        fval=fval_j;
    end
end
```

```
Fit.gn=P_fit(1);
Fit.kp=P_fit(2); % in radian units
Fit.kd=P_fit(3);
Fit.kt=P_fit(4);
Fit.td=P_fit(5);
end

function mse = FRFfiterr_Fit1_v1(P_fit,F,FRF,COH,J,mgh)
% FRFfiterr_Fit1_v1
%   Function to calculate error between experimental FRF data and
%   model FRF on each iteration performed by the fmincon function.
gn=P_fit(1); % sensory weight
kp=P_fit(2); % neural controller stiffness
kd=P_fit(3); % neural controller damping
kt=P_fit(4); % integrated torque feedback gain constant
td=P_fit(5); % time delay
%
%  Form model FRF estimate from current parameters
%
w=2*pi*F;
s=1i*w;
B=(ones(size(w)))./(J*(s.*s)-mgh*ones(size(w)));
NC=kd*s+kp;
FF=(kt*ones(size(w)))./(s);
TD=(cos(w*td)-1i*sin(w*td));
N=NC.*TD;
frf=(gn*B.*N)./(ones(size(w)) - FF.*N + B.*N);
%
% fit error
%
d2=(FRF-frf)./abs(frf); % normalize individual vectors by magnitude of fit at each
frequency
mse=d2*d2';
end

function y=decimate2(x,r)
% DECIMATE2 - performs a reduced sampling of the input vector x
%   by a factor of r.  Unlike the MATLAB function called decimate,
%   this function does not perform any pre-filtering on the
%   original waveform x.  The decimation is performed such that
%   y(1) = x(1).
%
if nargin < 2
    error('Not enough input arguments.')
end
if abs(r-fix(r)) > eps
    error('Resampling rate R must be a positive integer.')
end
if fix(r) == 1
    y = x;
    return
end
if r <= 0
    error('Resampling rate R must be a positive integer.')
end
list=1:r:max(size(x));
y=x(list);
end

function error_bars_log(x,y,yb,width)
%
%  Function to plot error bars at +/-yb around the data point y at x.  The
%  horizontal width of the ends of the error bar is given by width.  The
%  variable width is a logarithmic fraction so that width=0.1 would produce
%  an error bar with a width of 1/10 of the log axis.
%
```

```
w=width/2;
for n=1:max(size(x))
    plot([x(n) x(n)],[y(n)-yb(n) y(n)+yb(n)],'b-')
    xl=10^(log10(x(n))-w);
    xh=10^(w+log10(x(n)));
    plot([xl xh],[y(n)-yb(n) y(n)-yb(n)],'b-')
    plot([xl xh],[y(n)+yb(n) y(n)+yb(n)],'b-')
end
end

function SetPrintFullSize
%
% Set print area to full size and centered
%
% The paper size is 8.5x11 inches
% The lateral and bottom (or top) margins are both 0.25 inch
%
set(gcf,'PaperUnits','inches') % Start by setting PaperUnits to inches
papersize=[8.5 11]; %Set the PaperSize property
left = 0.25;  % Calculate a left margin that centers the figure horizontally on the paper
bottom = 0.25; % Calculate a bottom margin that centers the figure vertically on the paper
figure_width = papersize(1) - left*2;              % Initialize a variable for figure's
width.
figure_height = papersize(2) - bottom*2;          % Initialize a varible for figure's
height.
myfiguresize = [left, bottom, figure_width, figure_height];
set(gcf, 'PaperPosition', myfiguresize); % Set the figure size
end

function xout=DeSpike_EquiTest(xin,thresh,rate)
%   DeSpike_EquiTest.m           15-May-16
%
%   Program to remove single sample noise spikes from sway traces
%   recorded on EquiTest platform. Infrequent spikes apparently due
%   to EquiTest instrumentation problems when digitizing signals on
%   auxilliary A/D inputs channels
%
%   xin = recorded pot angle time series (deg)
%   thres = velocity threshold for detecting spikes (deg/s)
%   rate = sampling rate of xin (samples/s)
%   xout = de-spiked time series (deg)
xout=xin;
xind=cdiff(xin)*rate;
for i=2:length(xin)-1
    if (abs(xind(i-1))>thresh)&&(abs(xind(i+1))>thresh)
        xout(i)=(xout(i-1)+xout(i+1))/2;
    end
end
end

function x = cdiff(x)
%
%CDIFF  Central Difference function.  If X is a vector
% [x(1) x(2) ... x(n)], then CDIFF(X) returns a vector
% of central differences between every second element
% [x(2)-x(1)  (x(3)-x(1))/2  (x(4)-x(2))/2  ...
%  (x(n)-x(n-2))/2  x(n)-x(n-1)].
%
% For time series, divide result x by deltat for proper
% velocity scaling where deltat is time interval between
% adjacent points x(i) and x(i+1)
%
% If X is a matrix, the differences are calculated down
% each column.
%
% The first and last elements in CDIFF are the simple
```

```
% differences between adjacent elements, and the returned
% vector or matrix has the same dimensions as the original.
[m,n] = size(x);
if m == 1
    y = x(3:n) - x(1:n-2);
    x = [x(2)-x(1) y./2 x(n)-x(n-1)];
else
    y = x(3:m,:) - x(1:m-2,:);
    x = [x(2,:)-x(1,:); y./2; x(m,:)-x(m-1,:)];
end
end
```

Matlab program for creation of EquiTest stimuli.

```
% EquiTest_MakePRTS_v1.m      18-Oct-2011
%
% Script to generate 1) a PRTS stimulus and save it as an ASCII file
% that can be used on the EquiTest CRS research platform to drive visual
% or surface tilt stimuli and 2) a 120 s duration calibration trial.
%
% After creating the text file created by this program, open the text file
% in a text editor and enter 'DT=10' as the first line of the text file,
% and save the file. DT-10 tells the EquiTest software that the sampling
% interval is 10 ms (for 100/s sampling rate).
%
% For the PRTS stimulus:
%   Total samples = 80states * 25samples/state * 12cycles + 6*SampRate
%                 = 24600 samples
%                 = 246.0 s stimulus duration
% For the Calibration stimulus:
%   Total samples = 120*SampRate
%                 = 12000 samples
%                 = 120.0 s stimulus duration
%
%------------------------------------------------------------------------
% Created by Robert J. Peterka
% Email: peterkar@ohsu.edu
% Please refer to the paper: "Implementation of a Central Sensorimotor
% Integration Test for Characterization of Human Balance Control
% During Stance", R.J. Peterka, C.F. Murchison, L. Parrington, P.C. Fino, L.A. King
%
% The authors take no responsibility for use of this Matlab code.
%------------------------------------------------------------------------
%
clear
close all
Amp_pp=2;     % desired peak-to-peak amplitude of the PRTS

SampRate=100; % Max sample rate for EquiTest CRS system
cycles=12;       % desired number of PRTS cycles
[x,xi]=pseudogen3(4,[0 0 -1 1],[2 0 1 1],25); % 80 state PRTS, 2000 pts/cycle, asymmetric
xi=xi/(max(xi)-min(xi));    % normalize to 1 peak-to-peak
x2=Amp_pp*xi;
PRTS=x2;
for i=1:(cycles-1)
    PRTS=[PRTS x2];
end
PRTS=[zeros(1,2*SampRate) PRTS zeros(1,4*SampRate)];   % 2 s of zeros at start and end
(assuming 100/s sample rate)
PRTS=PRTS';     % change to single row format

[B,A]=butter(3,4.5/(SampRate/2));   % 4.5 Hz lowpass filter
PRTSf=filtfilt(B,A,PRTS);
```

```
t=(0:(length(PRTS)-1))/SampRate;
plot(t,PRTS,'b'); hold on; plot(t,PRTSf,'r'); hold off
title('PRTS stimulus, 80 state, 25 samples/state, unfiltered(b), 4.5 Hz filtered(r) ')

%save test PRTS -ASCII
[fid,message]=fopen(['PRTSfilter_4dot5Hz_',num2str(Amp_pp),'pp.txt'],'wt');
count=fprintf(fid,'%6.3f\r\n',PRTSf)
fclose(fid);

%%
% Make 120 s calibration trial
%
Cal=zeros(12000,1);
[fid,message]=fopen('Calib120.txt','wt');
count=fprintf(fid,'%6.3f\r\n',Cal)
fclose(fid);


%%
function [x,xi]=pseudogen3(n,fb,seed,ppdt)
%
% [x,xi]=pseudogen3(n,fb,seed,ppdt)
%
% Function to generate a pseudorandom ternary sequence.  The
% number of shift registers is n (1x1), the feedback is defined
% by fb (1xn), and starting values in the shift register are
% given by seed (1xn) where seed has values of 0,1,or 2.
%
% For fb = [-1 1], then x(1) = rem(fb*seed',3), and if x(1)
% is less than 0, the x(1) = x(1)+3.  For the final x output
% substitute -1 for all 2's in the sequence.
%
% The vector xi contains the integration of x with ppdt "points
% per delta t", i.e. each state of the shift register is divided
% into ppdt intervals and the integration is computed at each of the
% points.
%
% Valid feedback combinations are defined in Davies (1970) for shift
% register lengths from 2 to 7.  Davies gives a list of the "characteristic
% polynomials" that define all valid feedback configurations for maximal
% length sequences.  In order to translate these polynomials into the
% feedback array, fb, needed for this program, do the following:
%   1. The polynomial will always have one more digit than the number of
%      of feedback registers in the shift register.  The left-most digit
%      in the polynomial is always 1.  Ignore this digit.
%   2. The remaining digits will be a sequence of 0's, 1's, or 2's.  In order
%      to translate this sequence into the values included in the variable
%      fb, change all 2's to 1's, change all 1's to -1's, leave all 0's 0's.
%      So for example, one characteristic polynomial for a 5 stage shift
%      register is 100211.  Ignoring the left-most digit leaves 00211.  This
%      translates into the fb values fb=[0 0 1 -1 -1] used by this Matlab function.
%   3. The seed value can be any combination of 0's 1's or 2's.  The single
%      exception is that it cannot be all 0's.  Changing the seed value just
%      changes the starting point of the PRTS. So selecting the seed allows
%      you to change the symmetry of the integrated PRTS about zero.
%
% Examples of valid maximal length PRTS with 4, 5, 6, and 7 shift registers:
%   [x,xi]=pseudogen3(4,[0 0 -1 1],[2 0 1 1],ppdt); % 80 state sequence
%   [x,xi]=pseudogen3(5,[0 0 1 -1 -1],[2 0 2 0 2],ppdt);    % 242 state sequence
%   [x,xi]=pseudogen3(6,[1 0 1 1 1 1],[0 0 0 0 0 1],ppdt);  % 728 state sequence
%   [x,x1]=pseudogen3(7,[0 0 0 -1 -1 -1 -1],[2 2 0 2 2 2 2],ppdt); % 2186 state sequence
%   [x,x1]=pseudogen3(7,[0 0 0 -1 -1 -1 -1],[0 0 0 0 1 1 2],ppdt); % 2186 state sequence
%
% Reference: W.D.T. Davies, System Identification for Selt-Adaptive
% Control, Wiley-Interscience, London, 1970.
```

```
shiftreg=seed;
i=3^n-1;  % length of ternary sequence
x=zeros(1,i);
for j=1:i
    x(j)=rem(fb*shiftreg',3);
    if x(j)<0
        x(j)=x(j)+3;
    end
    shiftreg=[x(j) shiftreg(1:(n-1))];  % shift to right
end
for j=1:i       %assign -1 to 2
    if x(j)==2
        x(j)=-1;
    end
end
%
% integrate the sequence with ppdt points per sequence value (i.e., state)
%
xi=zeros(1,ppdt*i);
for j=1:i
    for jj=1:ppdt
        xi(jj+(j-1)*ppdt+1)=xi(jj+(j-1)*ppdt)+x(j);
    end
end
xi=xi(1:ppdt*i); % get rid of last point which repeats first point
end
```

Matlab program for creation of modified visual scene.

```
%  MakeEquiTestVisualScene_v1.m
%
%  Create poster for EquiTest CRS visual surround to provide a more
%  compelling visual scene. Scene created by combining horizontal and
%  vertical stipes base on a PRTS sequence.
%
%---------------------------------------------------------------------------
% Created by Robert J. Peterka
% Email: peterkar@ohsu.edu
% Please refer to the paper: "Implementation of a Central Sensorimotor
% Integration Test for Characterization of Human Balance Control
% During Stance", R.J. Peterka, C.F. Murchison, L. Parrington, P.C. Fino, L.A. King
%
% The authors take no responsibility for use of this Matlab code.
%---------------------------------------------------------------------------
%
order=4;
[x,xi]=pseudogen3(order,[0 0 -1 1],[1 1 1 1],2);
x=(x+1);
s=3^order-1;
factor=2;
I=zeros(s*factor,s*factor);
for i=1:s
  for j=1:factor
    I(:,(i-1)*factor+j)=ones(s*factor,1)*x(i);
  end
end
for i=1:s
  for j=1:factor
    I((i-1)*factor+j,:)=I((i-1)*factor+j,:)+ones(1,s*factor)*x(i);
  end
end

I=I*64;

colormap(gray(256))
```

```
image(I)
axis('square')
axis('off')
%%
function [x,xi]=pseudogen3(n,fb,seed,ppdt)
%
% [x,xi]=pseudogen3(n,fb,seed,ppdt)
%
% Function to generate a pseudorandom ternary sequence.  The
% number of shift registers is n (1x1), the feedback is defined
% by fb (1xn), and starting values in the shift register are
% given by seed (1xn) where seed has values of 0,1,or 2.
%
% For fb = [-1 1], then x(1) = rem(fb*seed',3), and if x(1)
% is less than 0, the x(1) = x(1)+3.  For the final x output
% substitute -1 for all 2's in the sequence.
%
% The vector xi contains the integration of x with ppdt "points
% per delta t", i.e. each state of the shift register is divided
% into ppdt intervals and the integration is computed at each of the
% points.
%
% Valid feedback combinations are defined in Davies (1970) for shift
% register lengths from 2 to 7.  Davies gives a list of the "characteristic
% polynomials" that define all valid feedback configurations for maximal
% length sequences.  In order to translate these polynomials into the
% feedback array, fb, needed for this program, do the following:
%   1. The polynomial will always have one more digit than the number of
%      of feedback registers in the shift register.  The left-most digit
%      in the polynomial is always 1.  Ignore this digit.
%   2. The remaining digits will be a sequence of 0's, 1's, or 2's.  In order
%      to translate this sequence into the values included in the variable
%      fb, change all 2's to 1's, change all 1's to -1's, leave all 0's 0's.
%      So for example, one characteristic polynomial for a 5 stage shift
%      register is 100211.  Ignoring the left-most digit leaves 00211.  This
%      translates into the fb values fb=[0 0 1 -1 -1] used by this Matlab function.
%   3. The seed value can be any combination of 0's 1's or 2's.  The single
%      exception is that it cannot be all 0's.  Changing the seed value just
%      changes the starting point of the PRTS. So selecting the seed allows
%      you to change the symmetry of the integrated PRTS about zero.
%
% Examples of valid maximal length PRTS with 4, 5, 6, and 7 shift registers:
%   [x,xi]=pseudogen3(4,[0 0 -1 1],[2 0 1 1],ppdt); % 80 state sequence
%   [x,xi]=pseudogen3(5,[0 0 1 -1 -1],[2 0 2 0 2],ppdt);    % 242 state sequence
%   [x,xi]=pseudogen3(6,[1 0 1 1 1 1],[0 0 0 0 0 1],ppdt);  % 728 state sequence
%   [x,x1]=pseudogen3(7,[0 0 0 -1 -1 -1 -1],[2 2 0 2 2 2 2],ppdt); % 2186 state sequence
%   [x,x1]=pseudogen3(7,[0 0 0 -1 -1 -1 -1],[0 0 0 0 1 1 2],ppdt); % 2186 state sequence
%
% Reference: W.D.T. Davies, System Identification for Selt-Adaptive
% Control, Wiley-Interscience, London, 1970.

shiftreg=seed;
i=3^n-1;  % length of ternary sequence
x=zeros(1,i);
for j=1:i
    x(j)=rem(fb*shiftreg',3);
    if x(j)<0
        x(j)=x(j)+3;
    end
    shiftreg=[x(j) shiftreg(1:(n-1))];  % shift to right
end
for j=1:i       %assign -1 to 2
    if x(j)==2
        x(j)=-1;
    end
end
```

```
%
% integrate the sequence with ppdt points per sequence value (i.e., state)
%
xi=zeros(1,ppdt*i);
for j=1:i
    for jj=1:ppdt
        xi(jj+(j-1)*ppdt+1)=xi(jj+(j-1)*ppdt)+x(j);
    end
end
xi=xi(1:ppdt*i); % get rid of last point which repeats first point
end
```
